# A bibliometric systematic review of extracellular vesicles in cutaneous malignant melanoma from 2005 to 2025

**DOI:** 10.3389/fonc.2026.1731250

**Published:** 2026-01-29

**Authors:** Natasha Christodoulides, Stephanie Bollard, Yashna Chabria, Lorraine O’Driscoll, Shirley Potter

**Affiliations:** 1 Department of Plastic and Reconstructive Surgery, St James’ Hospital, Dublin, Ireland; 2 School of Pharmacy and Pharmaceutical Sciences, Trinity College, Dublin, Ireland; 3 Trinity Biomedical Sciences Institute, Trinity College, Dublin, Ireland; 4 Trinity St. James's Cancer Institute, Department of Pharmacy and Pharmaceutical Sciences, Dublin, Ireland; 5 Royal College of Surgeons in Ireland, Dublin, Ireland

**Keywords:** bibliometric analysis (BA), biomarkers, cutaneous malignant melanoma (CMM), extracellular vescicles (EVs), liquid biopsy, pre-metastastic niche, tumour microenvirnonment

## Abstract

**Introduction:**

Cutaneous melanoma (CM) is among the most aggressive human malignancies characterised by its strong propensity for metastasis. Recent advances in melanoma research have reframed the disease as a systemic condition driven by dynamic interactions between tumour cells, the tumour microenvironment and the immune system. Increasing evidence indicates that these interactions are largely mediated by extracellular vesicles (EVs), small lipid-bilayer-enclosed particles that facilitate intercellular communication. EVs can be isolated from all biofluids, making them attractive minimally invasive biomarkers for diagnosis, staging, monitoring response to treatment and predicting relapse. Despite growing interest, a comprehensive overview of global research trends in this area is lacking.

**Methods:**

We performed a bibliometric systematic review of EVs-related CM research from 2005 to 2025 using Web of Science, Scopus, and EMBASE. Eligible studies focused on EVs characterisation, biomarker development, and functional roles in melanoma. Data were analysed using the *Bibliometrix* R-package to assess publication trends, citation metrics, author networks, institutional output and thematic evolution.

**Results:**

An analysis of the 288 included studies revealed that publication activity peaked in 2020, representing a 4000% increase in annual output compared to 2005. Additionally, a marked surge in citation frequency was observed beginning in 2018. The United States and China led in output, although international collaboration was limited. Chongqing Medical University and the University of Pittsburgh were among the most productive institutions. The *Journal of Extracellular Vesicles* published the highest number of articles in this field. Keyword and co-citation analysis identified major research themes, including immune evasion, biomarker discovery, and therapy resistance.

**Discussion:**

This first bibliometric analysis of EV research in CM reveals a rapidly expanding field with evolving research priorities. These findings offer a data-driven framework to guide future studies, promote collaboration, and inform strategic investment in EVs-based melanoma research.

## 1 Introduction

Cutaneous melanoma (CM) is one of the most aggressive human malignancies, characterised by its high metastatic potential. It is a malignant neoplasm of melanocytes, the pigment-producing cells which reside in the basal layer of the epidermis (1). Despite accounting for only approximately 1% of skin cancers, it is responsible for the majority of skin cancer-related deaths (2). Recent advances in melanoma research have reframed the disease as a systemic condition driven by dynamic interactions between tumour cells, the tumour microenvironment and the immune system (3, 4). Increasing evidence indicates that these interactions are largely mediated by extracellular vesicles (EVs) (5, 6).

EVs are a heterogeneous group of cell-derived membranous structures enclosed by a phospholipid bilayer. Based on their biogenesis, molecular composition, and size, EVs are typically classified into three subtypes: apoptotic bodies (50–2000 nm), microvesicles (50–1500 nm), and exosomes (50–120 nm) (7). These vesicles carry various bioactive molecules, including messenger RNA (mRNA), microRNA (miRNA), lipids, and proteins, and are capable of mediating intercellular communication over both short and long distances via bodily fluids (8). As such, EVs have gained considerable attention for their roles in disease progression, immune regulation, biomarker development, and emerging therapeutic strategies.

In recent years, EVs have been increasingly implicated in CM. They have been shown to facilitate immune escape by transporting immunosuppressive factors such as programmed death-ligand 1 (PD-L1) and transforming growth factor-beta (TGF-β), which interfere with antitumour immune responses (9, 10). Moreover, melanoma-derived EVs contribute to the remodelling of the tumour microenvironment and the establishment of pre-metastatic niches, thereby promoting disease progression and resistance to therapy. The molecular composition of EVs is highly dependent on the physiological state and origin of their parent cells, making them attractive minimally-invasive biomarkers for disease staging, monitoring treatment response, and predicting relapse (11, 12).

While the biological and clinical relevance of EVs in melanoma is progressing, a systematic understanding of how research in this area has evolved over time is lacking. A bibliometric analysis is a quantitative approach used to evaluate the scientific output and influence of publications within a defined field (13). It enables the identification of prolific authors, institutions, countries, and journals, as well as the detection of research hotspots and emerging trends through citation and keyword analysis (13, 14).

The aim of this study was to systematically map the global research landscape on extracellular vesicles in CM over the past two decades. Using established bibliometric tools, we sought to identify key contributors, visualise collaboration networks, assess citation impact, and uncover thematic evolution in the field. This analysis is intended to support researchers, funding agencies, and policy-makers by providing a structured overview of current trends and guiding future directions in this rapidly advancing area of cancer research.

## 2 Methods

### 2.1 Literature search and study selection

A comprehensive literature search was conducted using the Web of Science Core Collection (WoSCC), Scopus and EMBASE databases for peer-reviewed literature published between the 1^st^ of January 2005 and 31^st^ of March 2025 inclusive. The literature search was performed with the assistance of a trained librarian. The search terms used were ((“extracellular vesicle*” OR “exosome*” OR “microvesicle*” OR “cell-derived microparticle*” OR “small vesicle*” OR “ectosome**) AND (“cutaneous melanoma*” OR “melanoma*” OR “cutaneous malignant melanoma*” OR “cutaneous metastatic melanoma*”) AND (“biomarker*” OR “liquid biops*”) NOT (“uveal melanoma*” OR “oral melanoma*” OR “Merkel cell”)). The key words were chosen as they were broad ranging enough to include all relevant articles, while limiting output to the specific research question. The search was limited to the English language and to journal articles only as these represent the most up-to-date scientific repository. A dedicated search of the “grey literature” (unpublished trials, theses, reports and conference notes) was not undertaken as sufficient data was obtained through the conventional literature search. No restrictions on study design were set in the initial search. The abstracts identified were independently reviewed by two reviewers using the Rayyan software (15). Any disagreement between the reviewers was highlighted. The full-text articles were then reviewed by a third reviewer and a final consensus was reached.

### 2.2 Eligibility criteria

All studies that investigated the role of EVs in CM between 2005 and 2025 were included. This included studies: (a) characterising EVs cargo, (b) investigating the role of EVs as biomarkers, or (c) linking EVs with CM progression, metastasis, resistance or response to therapy (**Table 1**).

**Table 1:** Summary of inclusion and exclusion criteria.

Inclusion criteria	Exclusion criteria
Studies analysing EVs in cutaneous melanoma *in vitro* (*melanoma cells only)*, *in vivo* (*human and murine)* or clinical studies	Studies on non-melanoma cancers or other subtypes of melanoma e.g. mucosal/uveal
EV cargo characterisation (miRNAs, proteins, lipids, DNA)	Reviews, commentaries, case reports
Studies linking EVs with: • Disease progression • Metastasis • Therapy resistance or response	Studies lacking EVs-specific analysis
EVs as potential biomarkers (diagnostic, prognostic or predictive)	Studies without clear methodology for EVs isolation and characterisation
	Studies on EVs as transporters for therapeutic agents

Review articles and articles relating to the role of EVs in other biological or pathological processes not specific to CM were not deemed eligible for inclusion. Studies without a clear methodology for EVs isolation and characterisation, studies lacking EVs-specific analysis and studies looking into EVs as transporters for therapeutic agents were also excluded (**Table 1**). Finally, we excluded animal studies *except* murine models. This was to ensure data homogeneity and reduce inter-species variability in our data.

### 2.3 Analysis tool

Several software tools are available to analyse and visualise bibliometric data. *Bibliometrix*, a free R statistical package, developed by Aria and Cuccurullo (14), was employed in this study to analyse and map bibliographic data (16). *Bibliometrix* for RStudio incorporates *Biblioshiny*, a graphical interface that enables non-coders to perform comprehensive analyses with optimised visual representations. Compared to most free software (e.g. *CiteSpace* or *VOSviewer*), *Bibliometrix* does not only focus on the data visualisation but also on the correctness and statistical completeness of the results (16). It combines bibliometric techniques such as co-word analysis, co-citation network analysis and generation of collaboration networks to analyse a research field’s evolution (17). It was, therefore, felt that this software would most completely assess and depict publication trends, author networks, institutional productivity, international collaborators, keyword co-occurrence and citation metrics in the field of EVs in CM.

Thresholds for bibliometric network inclusion were chosen to optimise interpretability while preserving literature coverage. Minimum occurrence cut-offs limited the influence of sparsely connected nodes, and sensitivity analyses demonstrated that varying the threshold resulted in no substantive change in cluster composition or thematic interpretation.

### 2.4 Data analysis

First, the included articles were imported into the WoSCC database as it is compliant with the *Bibliometrix* software and allows comprehensive data analysis through its online analysis tool. The WoSCC analysis tool was used to yield bibliometric parameters including the number of annual publications, the output of countries/regions, total citations per publication (CPP) and the Hirsch index (H-index). The H-index is commonly applied to quantify and standardise the scientific impact of researchers, institutions, and countries. Higher H-indices generally correspond to greater academic influence (18). Journal Impact Factors (IF) and quartile rankings were retrieved from the 2025 edition of Journal Citation Reports (JCR).

The latest version 2025.05.0 + 496 of RStudio for mac was downloaded and a *bibliometrix* package was established within the R environment to analyse and map the bibliographic data. The included studies were exported in BibTex format, before being uploaded to *Biblioshiny* for analysis. The *bibliometrix* functions were then used to create descriptive and co-citation networks.

## 3 Results

### 3.1 Literature review

**Figure 1:**
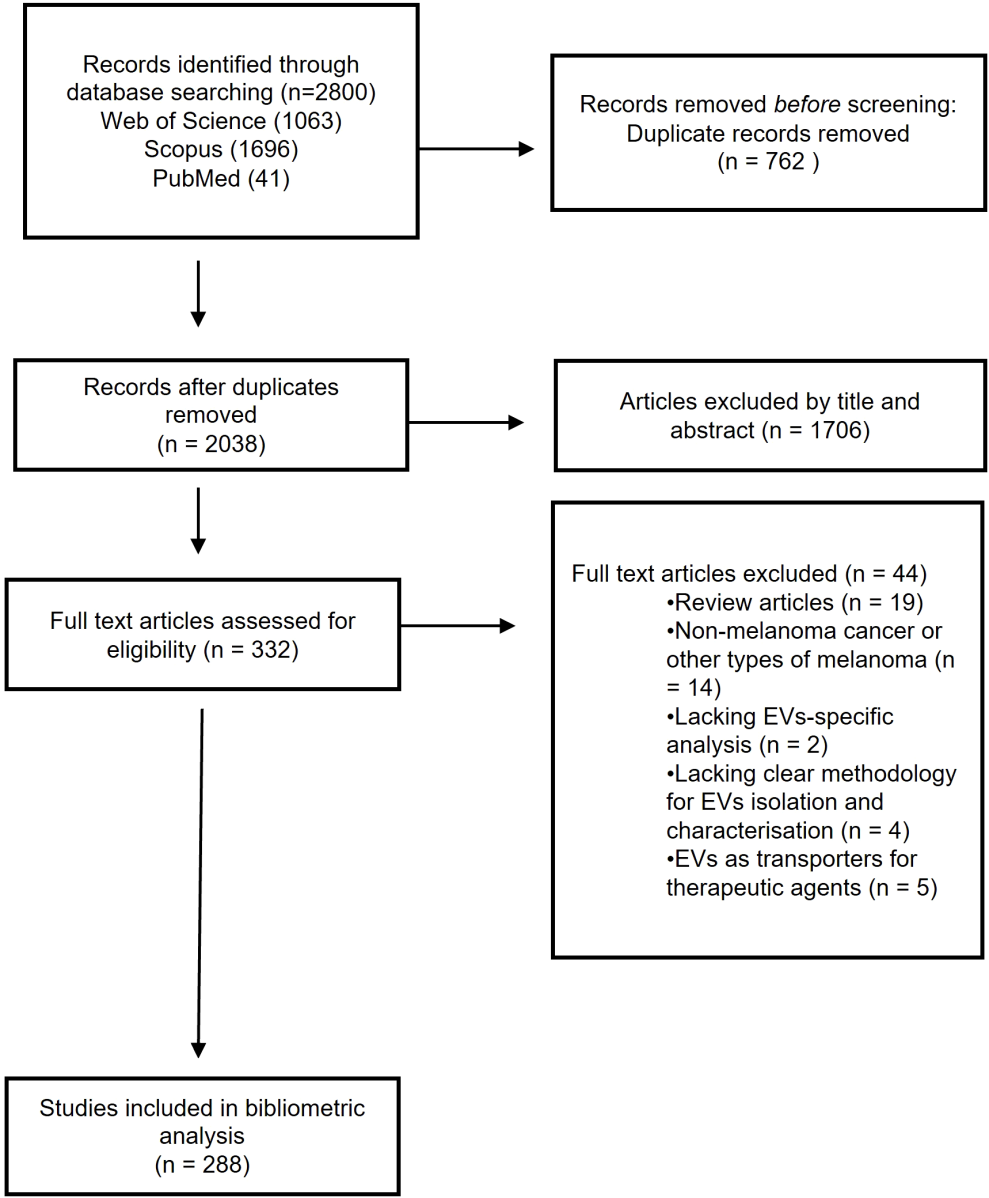
PRISMA 2020 flow diagram.

The “Preferred Reporting Items for Systematic Reviews and Meta-Analyses” (PRISMA) guidelines were used for study selection in this bibliometric analysis. The initial search yielded 2800 publications; which was reduced to 2038 after duplicates were removed. 1706 papers were excluded by title, abstract and keywords alone, leaving 332 manuscripts for full-text review. 44 articles were deemed ineligible after full-text review and the remaining 288 articles were deemed suitable for inclusion in the bibliometric analysis. The reasons as to why the articles were excluded are listed in the PRISMA flow diagram (**Figure 1**).

### 3.2 Annual number of publications and citations

**Figure 2:**
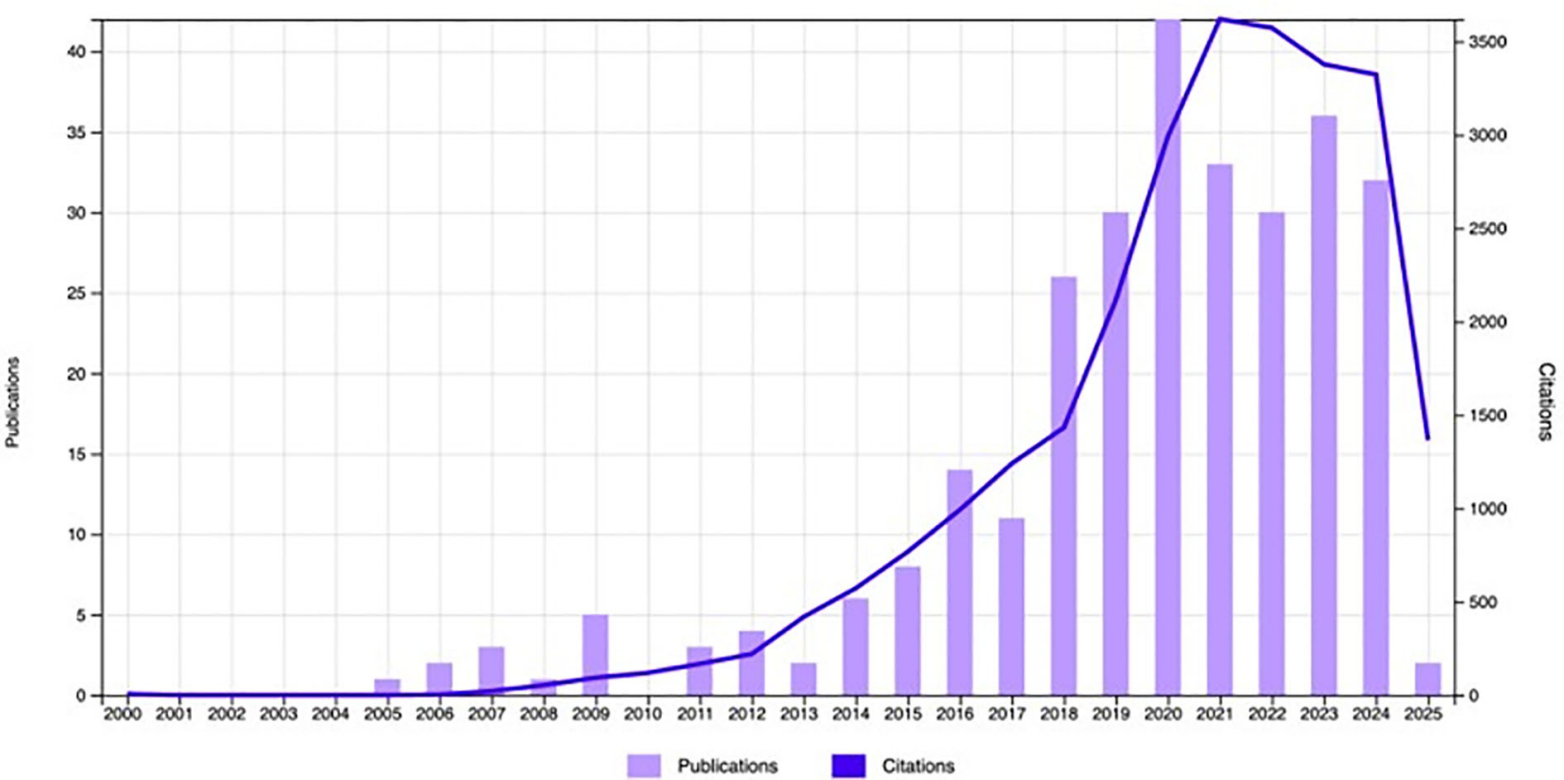
Annual number of publications worldwide from 2005 to 2025. Plotted against the yearly number of worldwide citations. Figure generated from WoSCC.

Between January 1^st^ 2005 and March 31^st^ 2025 a total of 288 publications met our inclusion criteria. The growth of the annual publication output and annual citations are shown in **Figure 2**. Compared to 2005 (n=1), the growth rate of publications in the field was 4100%, with 42 publications in 2020. There was subsequently a slight decrease in publication output between 2020 and 2023 possibly stemming from the overall reduction in original research output during the COVID-19 pandemic (19). Notably, the highest surge in citations was in 2021 (n=33, citations = 3620), and remained high up to and including 2024, reflecting the significance of this rapidly expanding research area. The top 10 most cited publications are shown in **Table 2**. The most frequently cited study was published in *Nature Medicine* in 2012 by Peinado et al. entitled “*Melanoma exosomes educate bone marrow progenitor cells toward a pro-metastatic phenotype through MET”*, with 3,238 citations (20). The annual citation rate (ACR), which was calculated as the ratio between the number of citations and the number of years since its publication, is 231.29 citations per year for this article. The second most cited article (citations = 2,326; ACR = 290.75) was published in *Nature* by Chen and colleagues in 2018 and is entitled *“Exosomal PD-L1 contributes to immunosuppression and is associated with the anti-PD-1 response” (*21).

**Table 2:** Top 10 most cited publications.

Rank	Title	First author	Journal	Country	IF (2023)	Category quartile	Publication year	Total citations^a^	ACR^a^
1	Melanoma exosomes educate bone marrow progenitor cells toward a pro-metastatic phenotype through MET	Peinado, H	Nature Medicine	USA	58.7	Q1	2012	3,238	231.29
2	Exosomal PD-L1 contributes to immunosuppression and is associated with the anti-PD-1 response	Chen, G	Nature	USA	50.5	Q1	2018	2,326	290.75
3	Microenvironmental pH is a Key Factor in Exosome Traffic in Tumour Cells	Parolini, I	Journal of Biological Chemistry	Italy	4	Q2	2009	1,362	80.12
4	Malignant effusions and immunogenic tumour-derived exosomes	Andre, F	Lancet	France	98.4	Q1	2002	937	39.04
5	Exosomes released by melanoma cells prepare sentinel lymph nodes for tumour metastasis	Hood, JL	Cancer Research	USA	12.5	Q1	2011	917	61.13
6	High levels of exosomes expressing CD63 and caveolin-1 in plasma of melanoma patients	Logozzi, M	PLOS ONE	Italy	2.9	Q1	2009	867	51
7	Induction of lymphocyte apoptosis by tumour cell secretion of FasL-bearing microvesicles	Andreola, G	Journal of Experimental Medicine	Italy	12.8	Q1	2002	677	28.21
8	Visualisation and *in vivo* tracking of the exosomes of murine melanoma cells B16-BL6 cells in mice after intravenous injection	Takahashi, Y	Journal of Biotechnology	Japan	4.1	Q1	2013	633	48.69
9	Tumour-derived microvesicles promote regulatory T cell expansion and induce apoptosis in tumour-reactive activated CD8+ T lymphocytes	Wieckowski, EU	Journal of Immunology	USA	3.6	Q2	2009	500	29.41
10	Human tumour-released microvesicles promote differentiation of myeloid cells with transforming growth factor-β-mediated suppressive activity on T lymphocytes	Valenti, R	Cancer Research	Italy	12.5	Q1	2006	490	24.5

a Citation rates are according to WoSCC; ACR: annual citation rate, citations/year.

### 3.3 Distribution of countries/regions and institutions

**Figure 3:**
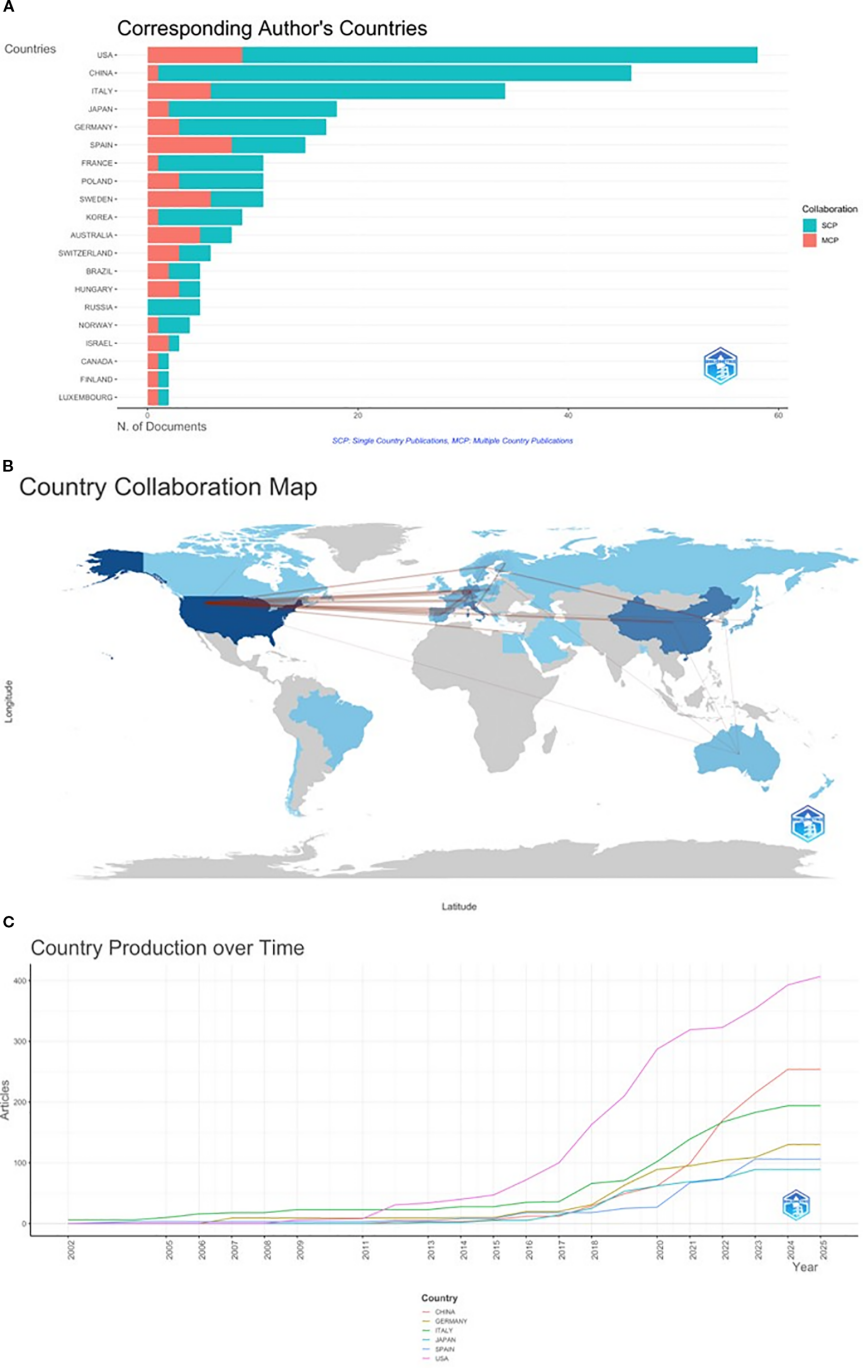
**(a)** Chart demonstrating corresponding author’s countries. Collaborative studies are depicted in orange. *SCP: Single Country Publications, MCP: Multiple Country Publications***(b)** Country collaboration map depicting collaborations in multiple country publications. All countries coloured in a shade of blue are actively involved in CM EVs research. The darker the shade of blue, the higher the productivity from that country. Connecting lines indicate co-authorship between countries, with thicker lines representing stronger collaborative relationships. **(c)** Top 6 most productive countries’ production over time. x-axis: year; y-axis: total number of articles published per year.

The distribution of all 288 articles covered 40 countries. The 10 countries with the highest publication productivity are illustrated in **Table 3**. The United States ranked first in the number of publications (27.8% of 288 articles; n=82), followed by China (16.61%, n=49), Italy (13.9%, n=41), Germany (11.53%, n=34) and Japan (6.78%, n=20). Notably, collaborations between countries were included in the counts, resulting in a higher number of counts than included studies i.e. some studies had more than one country/region of origin. The collaboration between countries is further reflected in **Figure 3a**. Based on the distribution of single-country (SCP) versus multiple -country publications (MCP) shown, international collaboration in the field of EVs in CM appears limited, with relatively few publications involving cross-country collaboration. Interestingly, China which is the second most productive country, has the lowest collaboration rate (2.2%), while the United States has a 15.5% collaboration rate (**Figure 3a**). This shows that there is still great scope for collaboration in the field of EVs in CM.

**Table 3:** Top 10 most productive countries/regions.

Rank	Country	Counts	% of 288	Citations WoS	Ave. Citations per paper	H-index
1	USA	82	27.80	12,898	157.29	44
2	China	49	16.61	3,950	80.61	21
3	Italy	41	13.90	6,047	147.49	26
4	Germany	34	11.53	1,735	51.03	19
5	Japan	20	6.78	1,845	92.25	15
6	Spain	19	6.44	4,234	222.84	13
7	France	15	5.09	1,743	116.2	8
8	Poland	14	4.75	454	32.43	9
9	Sweden	13	4.41	1,146	88.15	10
10	South Korea	12	4.07	700	58.33	10

International cooperation network analysis among different countries/regions in EVs melanoma research is shown in **Figure 3b**. Based on this collaboration map, link strength represents the degree of international research collaboration, measured by the number of co-authored publications between countries. Countries depicted in a darker shade of blue have demonstrated higher productivity in the field. Further analysis revealed that the closest collaborative relationship with the United States was Germany (n=13), followed by Switzerland (n=6), Spain (n=6), Italy (n=5) and France (n=4). Within Europe, collaboration was observed primarily among Western and Southern European research hubs, including Germany, France, Spain and Italy, while broader inter-European collaboration was limited (**Figure 3b**).

The annual publication output of the six most productive countries was identified. As demonstrated in **Figure 3c**, the most prolific country for annual publications was the United States. This leading position was maintained by the United States between 2012 and 2025 and demonstrates the impact this country has had on the current research landscape in the field. Interestingly, while China only began to stand out in the field of EVs research in melanoma in 2020, the country has demonstrated a very rapid increase in scientific production between 2020 and 2025 (**Figure 3c**).

Among the top 10 countries with regards to citations, the United States was the most cited (12,898), followed by Italy (6,047) and Spain (4,234). Spain however had the highest average citations per paper (222.84), largely attributed to the landmark paper by Peinado H. et al. (20) (**Table 3**).

**Figure 4:**
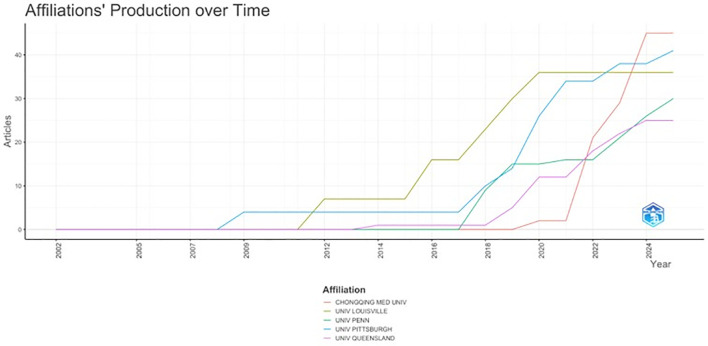
Top 5 affiliations’ production over time. x-axis: year; y-axis: total number of articles published per year.

The distribution of institutional affiliations consists of 612 entries. The 10 most relevant affiliations are shown in **Table 4**, with Chongqing Medical University having the highest output (n=45), followed by the University of Pittsburgh (n=41) and University of Louisville (n=36). Interestingly, Chongqing Medical University entered the field of EV research in CM in 2017, 9 years after the University of Pittsburgh, yet has shown a remarkable surge in research output in the past 5 years (**Figure 4**).

**Table 4:** Top 10 institutional affiliations.

Affiliation	Articles
Chongqing Medical University	45
University of Pittsburgh	41
University of Louisville	36
University of Pennsylvania	30
University of Queensland	25
Spanish National Cancer Research Centre CNIO	23
University of Gothenburg	23
Wuhan University	20
Mayo Clinic	18
Jagiellonian University	17

### 3.4 Distribution of journals

The articles included in this study were published in 151 journals. The top 10 most productive journals are shown in **Table 5**. The CiteScore represents the number of citations a journal receives relative to the number of articles it publishes.

**Table 5:** The top 10 scholarly journals that published research on EVs in CMM.

Rank	Journal	Count	IF 2023*	CiteScore	H-index
1	JOURNAL OF EXTRACELLULAR VESICLES	15	15.5	27.4	126
2	INTERNATIONAL JOURNAL OF MOLECULAR SCIENCES	14	4.9	6.9	244
3	SCIENTIFIC REPORTS	10	3.8	6.9	149
4	CANCERS	9	4.5	8.0	133
5	PLOS ONE	8	2.9	5.6	467
6	FRONTIERS IN ONCOLOGY	7	3.5	5.2	156
7	CANCER RESEARCH	6	12.5	16.3	483
8	FRONTIERS IN IMMUNOLOGY	5	5.7	9.8	190
9	PIGMENT CELL & MELANOMA RESEARCH	5	3.9	7.1	120
10	CANCER LETTERS	4	9.1	16.1	203

*The 2023 impact factor was used for all journals to ensure consistency and comparability across the dataset within the same citation environment.

The *Journal of Extracellular Vesicles* was the most productive journal for publishing research related to the role of EVs in CM diagnosis, pathogenesis, progression and monitoring, with a total of 15 publications (IF 15.5), followed by the *International Journal of Molecular Sciences* (n=14), *Scientific Reports* (n=10), *Cancers* (n=9) and *PLOS ONE* (n=8), respectively. The *Journal of Extracellular Vesicles* had the highest overall CiteScore and impact factor, while Cancer Research has the highest H-index (**Table 5**).

### 3.5 Analysis of authors and cited authors

**Figure 5:**
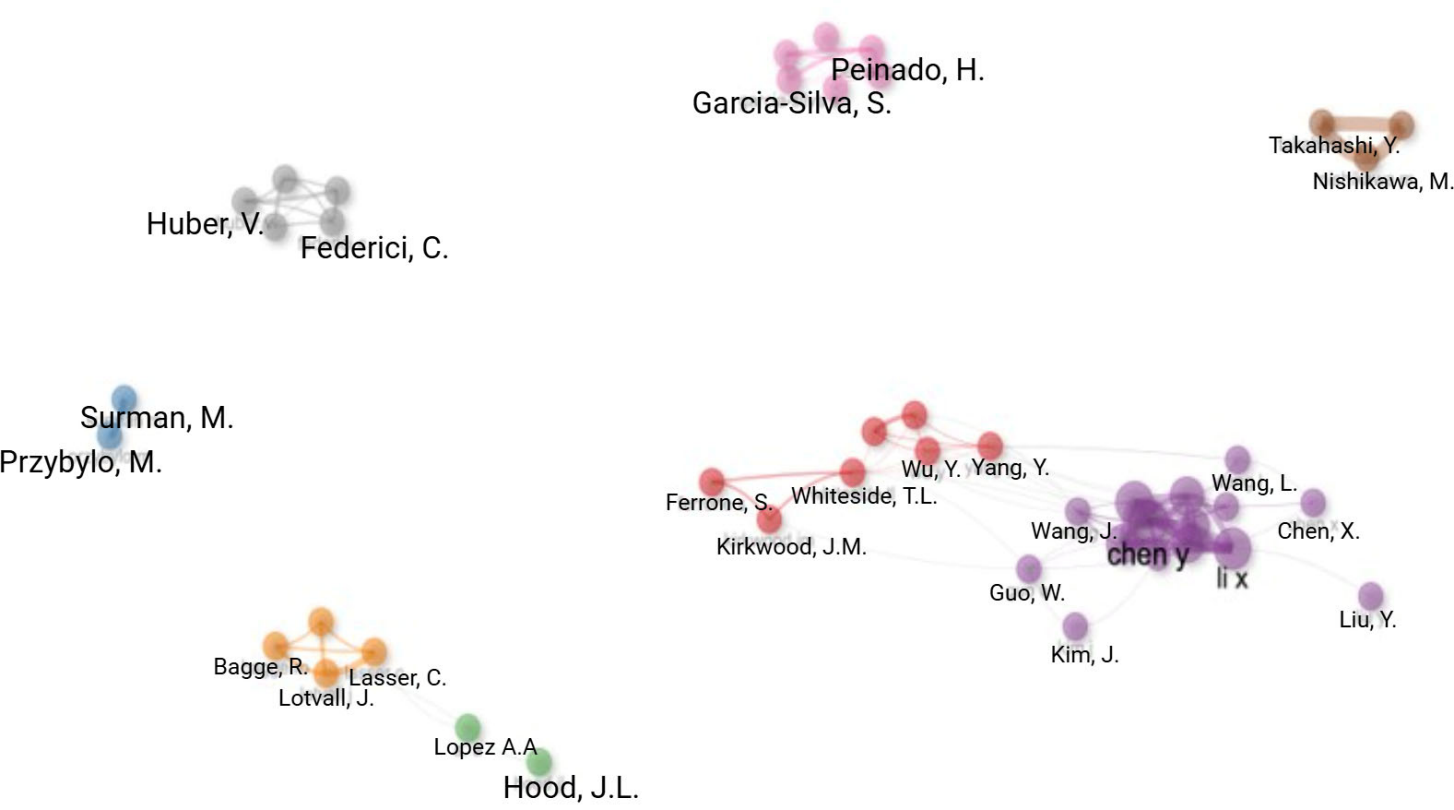
Author collaboration map. Each node represents an individual author, with node size proportional to publication output within the analysed literature. Lines between nodes indicate co-authorship relationships, with line thickness reflecting the strength of collaboration based on the number of shared publications. Spatial proximity denotes the degree of collaborative relatedness between authors. Colours identify clusters of closely collaborating authors, representing research groups or collaborative communities within the field. Authors were included if they met the minimum publication threshold of two, and collaborations were defined by co-authorship within the same publication.

The authors of the 288 publications were analysed. When considering publication outputs, Chen Y, was the most productive author with 14 publications, followed by Wang J (n=12), Li X (n=11), Hood JL (n=10) and Zhang Y (n=10). Based on H-index, Hood JL had the highest local impact with an H-index of 10, followed by Chen Y and Wang J, both with an H index of 9 (**Table 6**). **Figure 5** illustrates the author collaboration network of EVs research in CM. To reflect the relatively small size of the field while maintaining network interpretability, authors were included if they had contributed to at least two publications; inclusion of single-publication authors resulted in a highly fragmented network, whereas higher thresholds excluded a substantial proportion of active contributors. Collaborations were defined by co-authorship within the same publication. Node size is proportional to the number of publications per author, while links represent co-authorship relationships, with link thickness corresponding to collaboration strength (number of shared publications). Colours indicate clusters of authors identified algorithmically based on the density of collaborative links.

**Table 6:** Top 10 most relevant authors (2005 – 2025).

Authors	Author affiliation (city, country)	Articles	Articles fractionalised^a^	H-index
CHEN Y	Chongqing, China	14	1.14	9
WANG J	Chongqing, China	12	1.16	9
LI X	Chongqing, China	11	1.11	8
HOOD JL	Louisville, USA	10	3.97	10
ZHANG Y	Chongqing, China	10	0.93	8
WHITESIDE TL	Pittsburgh, USA	9	2.75	7
LI J	Chongqing, China	8	0.58	7
LIU D	Chongqing, China	8	0.74	6
PEINADO H	Madrid, Spain	8	0.72	6
HUBER V	Rome, Italy	7	0.49	7

a based on the number of authors per publication.

The network demonstrates a fragmented collaboration structure, characterised by several small, discrete clusters with limited inter-cluster connectivity. A dominant collaborative cluster is centred around a small number of highly productive authors, including Chen Y and Li X, who show multiple co-authorship links within this group. An important publication by Chen Y and colleagues published in *Gene Cancer Therapy* in 2024 demonstrates that transfer of exosomal lncRNA from highly metastatic melanoma stem cells to low-metastatic non-stem cells leads to activation of the *Wnt* signalling pathway and contributes to low-metastatic non-stem cells acquiring metastatic competency (22). In contrast, most other authors form smaller, isolated clusters or dyads, with minimal connections to the wider network. Overall, the limited number of links between clusters suggests that author-level collaboration in this field remains largely confined to individual research groups, with relatively few cross-group or cross-network partnerships.

**Figure 6:**
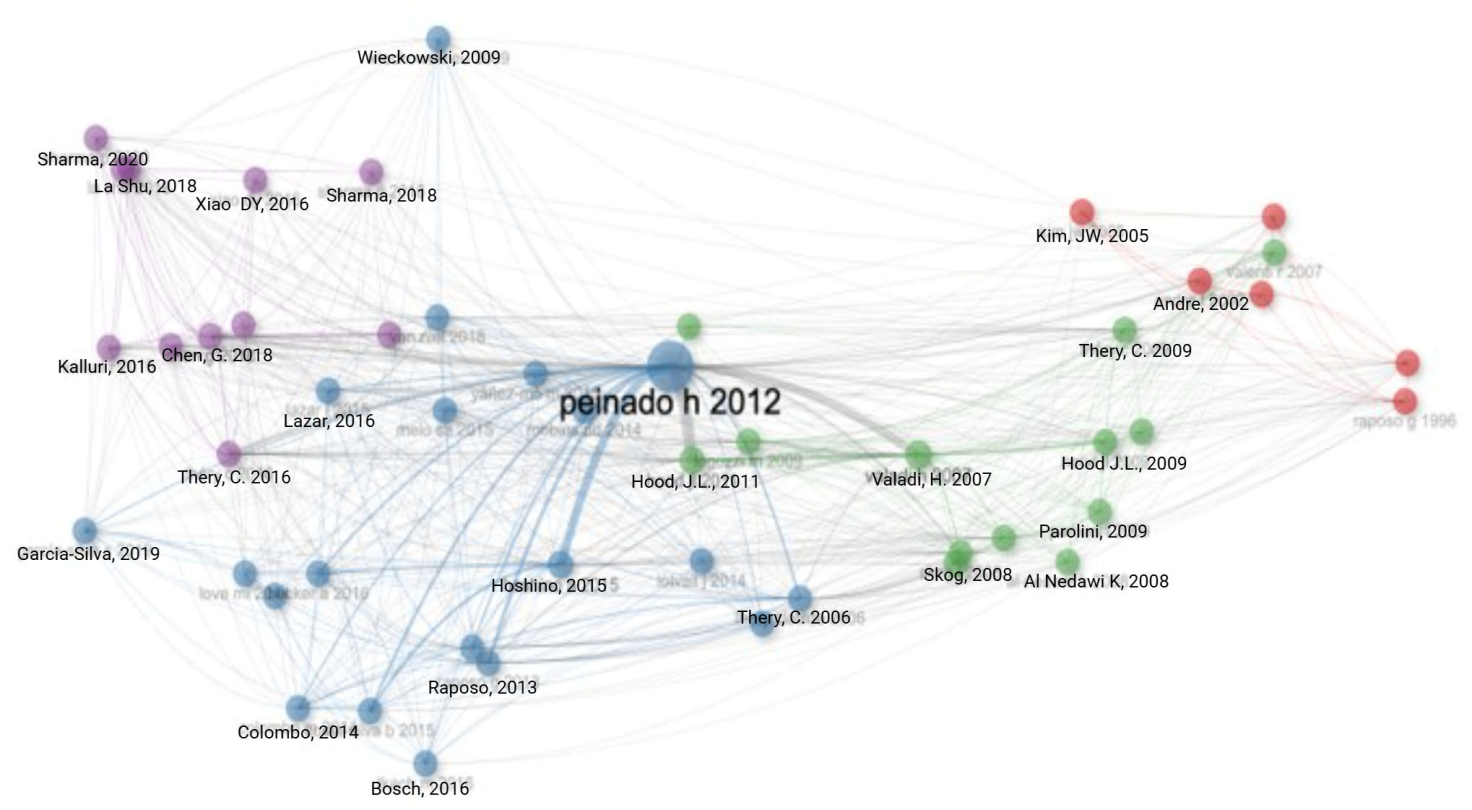
Author co-citation network. Multi-dimensional analysis illustrates stronger associations with wider lines connecting the relevant nodes. The colour distribution of authors in the network suggests the presence of distinct collaborative subfields within the broader academic domain.

The most cited author was Peinado H, for the paper published in *Nature Medicine* entitled *“Melanoma exosomes educate bone marrow progenitor cells toward a pro-metastatic phenotype through MET”* (20). An author co-citation network, illustrated through multidimensional scaling is shown in **Figure 6**. Each node represents an individual publication, with node size proportional to citation frequency within the dataset. Links between nodes indicate citation relationships, with thicker links reflecting stronger citation connections. Colours denote clusters of publications identified algorithmically based on citation patterns, representing groups of studies that are more closely related in terms of referenced literature. The network demonstrates a structured citation landscape with several interconnected clusters spanning different publication periods. A highly central and influential node is represented by the publication by Peinado et al. (2012), which occupies a prominent position within the network and shows extensive citation links across multiple clusters, indicating its role as a foundational reference in the field. Earlier studies form more peripheral clusters, while later publications appear more densely interconnected.

### 3.6 Analysis of keywords and trend topics

**Figure 7:**
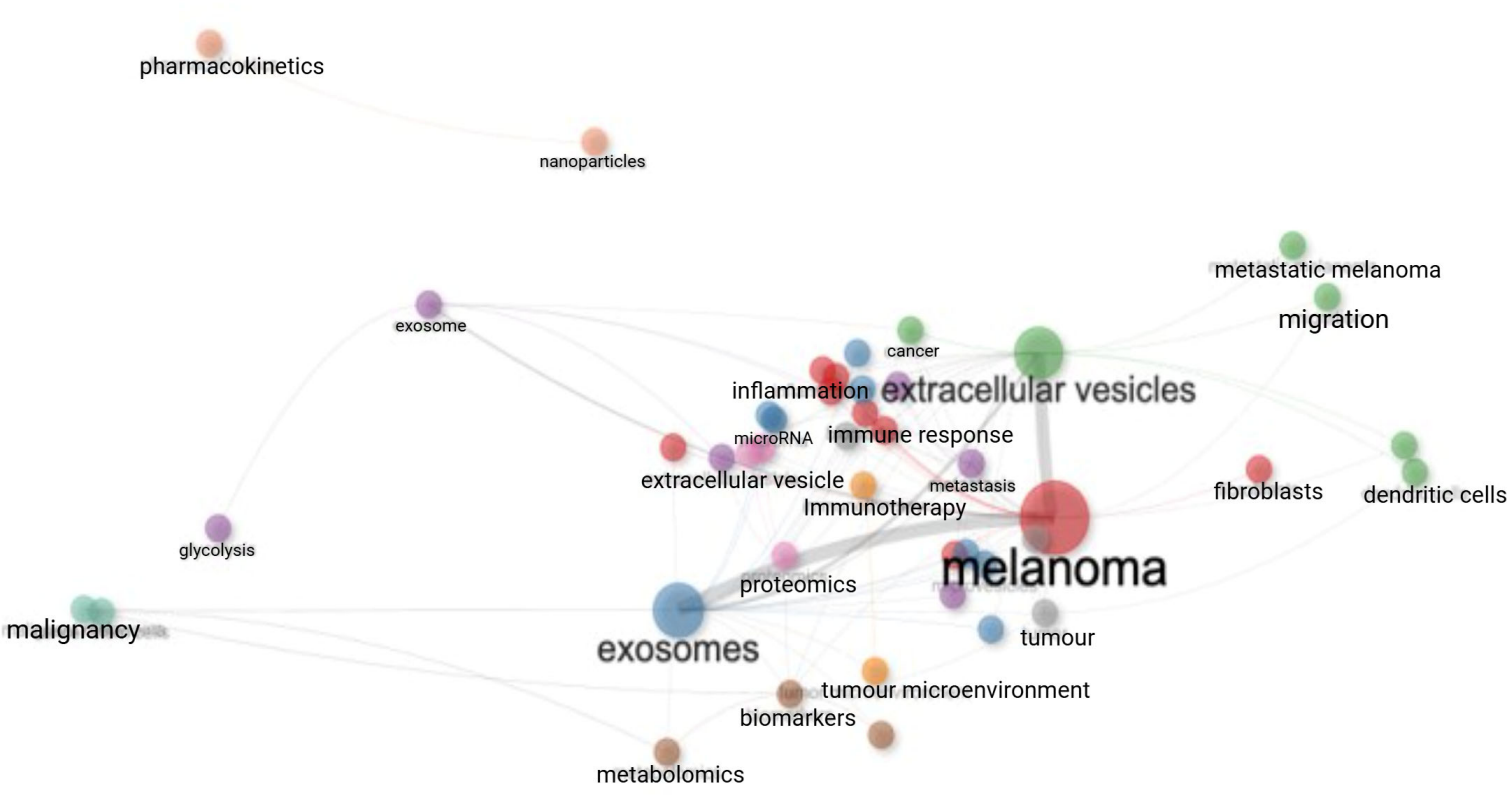
Map of keyword associations. Nodes represent individual keywords, with node size proportional to the frequency with which each keyword appears across the included publications. Links indicate co-occurrence of keywords within the same articles, with link thickness reflecting the strength of association. Spatial proximity denotes the degree of relatedness between keywords. Colours identify clusters of frequently co-occurring keywords, representing distinct but overlapping thematic areas within the literature.

A total of 251 keywords were identified, divided into 16 clusters as depicted in **Figure 7**. Each node represents an individual keyword, with node size proportional to its frequency of occurrence across the included publications. Links between nodes indicate co-occurrence of keywords within the same articles, with thicker links reflecting stronger associations. Colours denote clusters of keywords identified algorithmically based on co-occurrence patterns, representing related thematic areas within the literature. The map is dominated by the central keywords “melanoma” and “extracellular vesicles,” which exhibit the highest frequencies and the strongest associations with multiple surrounding terms. Closely linked keywords include “exosomes,” “tumour microenvironment,” “metastasis,” “biomarkers,” and “immune response,” reflecting key biological and translational themes explored in EV research in CM.

**Figure 8:**
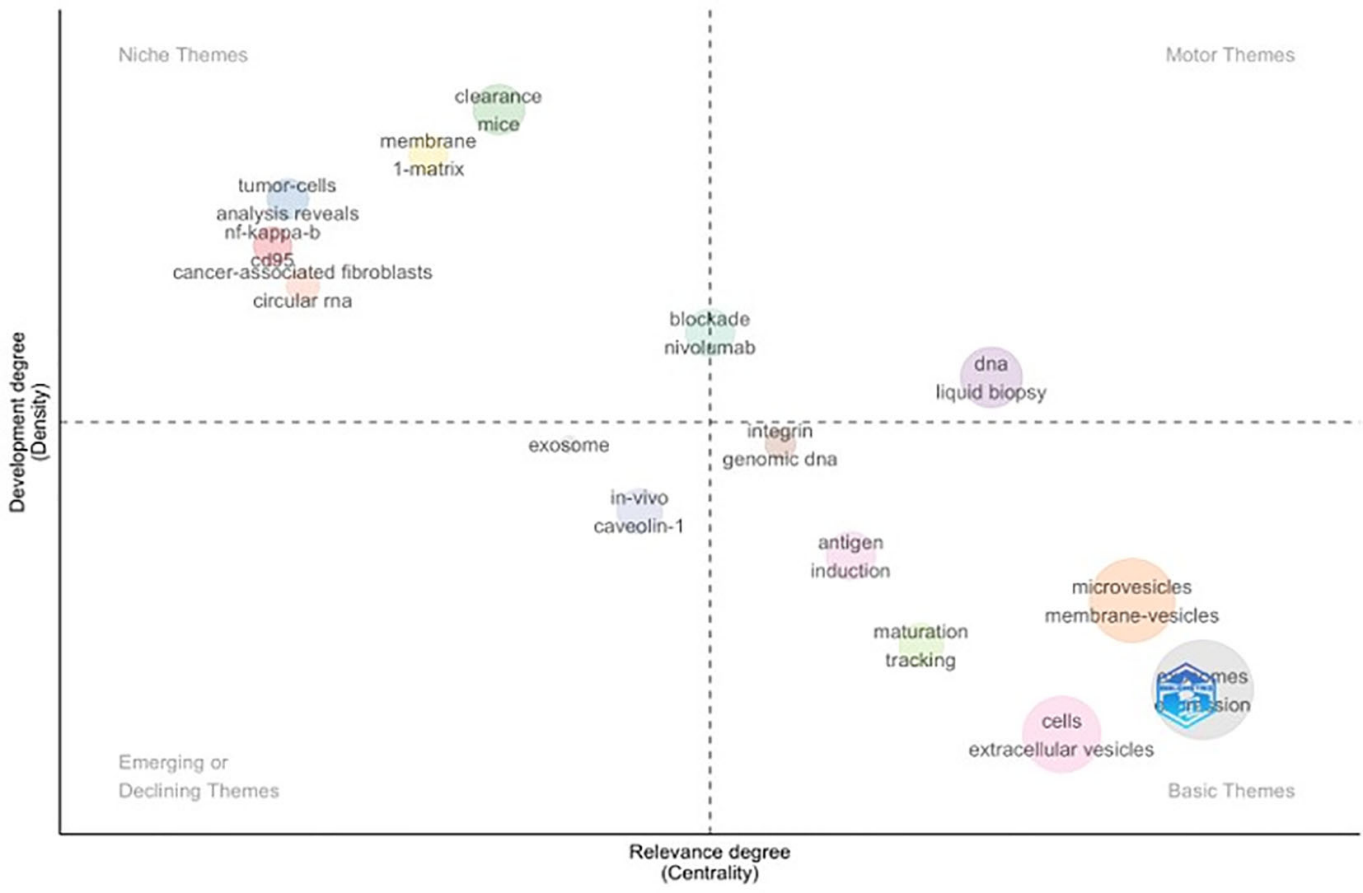
Thematic map using keywords plus. The map displays themes derived from Keywords Plus, with each bubble representing a thematic cluster of related keywords. Bubble size is proportional to the frequency of Keywords Plus within each theme. The horizontal axis (centrality) reflects the degree of interaction of a theme with other themes, indicating its relevance within the overall research field. The vertical axis (density) represents the internal coherence of each theme, reflecting the level of development and specialisation. Colours differentiate thematic categories, including motor, niche, emerging or declining, and basic themes.

**Figure 8** presents a thematic map derived from the Keywords Plus analysis, illustrating the structure and development of research themes in extracellular vesicle studies in cutaneous melanoma. Themes were generated based on co-occurrence patterns of Keywords Plus terms and positioned according to their relevance (centrality) on the x-axis and degree of development (density) on the y-axis. Bubble size reflects the frequency of Keywords Plus within each theme. Basic themes, located in the lower right quadrant, are characterised by high relevance but lower internal development and include broadly defined topics such as “extracellular vesicles,” “cells,” and “membrane vesicles,” reflecting foundational concepts that underpin much of the literature. Motor themes, situated in the upper right quadrant, demonstrate both high relevance and high development, with Keywords Plus such as “DNA,” “liquid biopsy,” and immune checkpoint–related terms (e.g. “nivolumab”), indicating well-established and actively investigated areas.

Niche themes in the upper left quadrant, including terms related to cancer-associated fibroblasts, circular RNA, and matrix interactions, represent more specialised and internally cohesive research areas with limited connectivity to the broader field. Emerging or declining themes, located in the lower left quadrant, include Keywords Plus such as “*in vivo*,” and “caveolin-1,” suggesting areas of evolving research focus.

**Figure 9:**
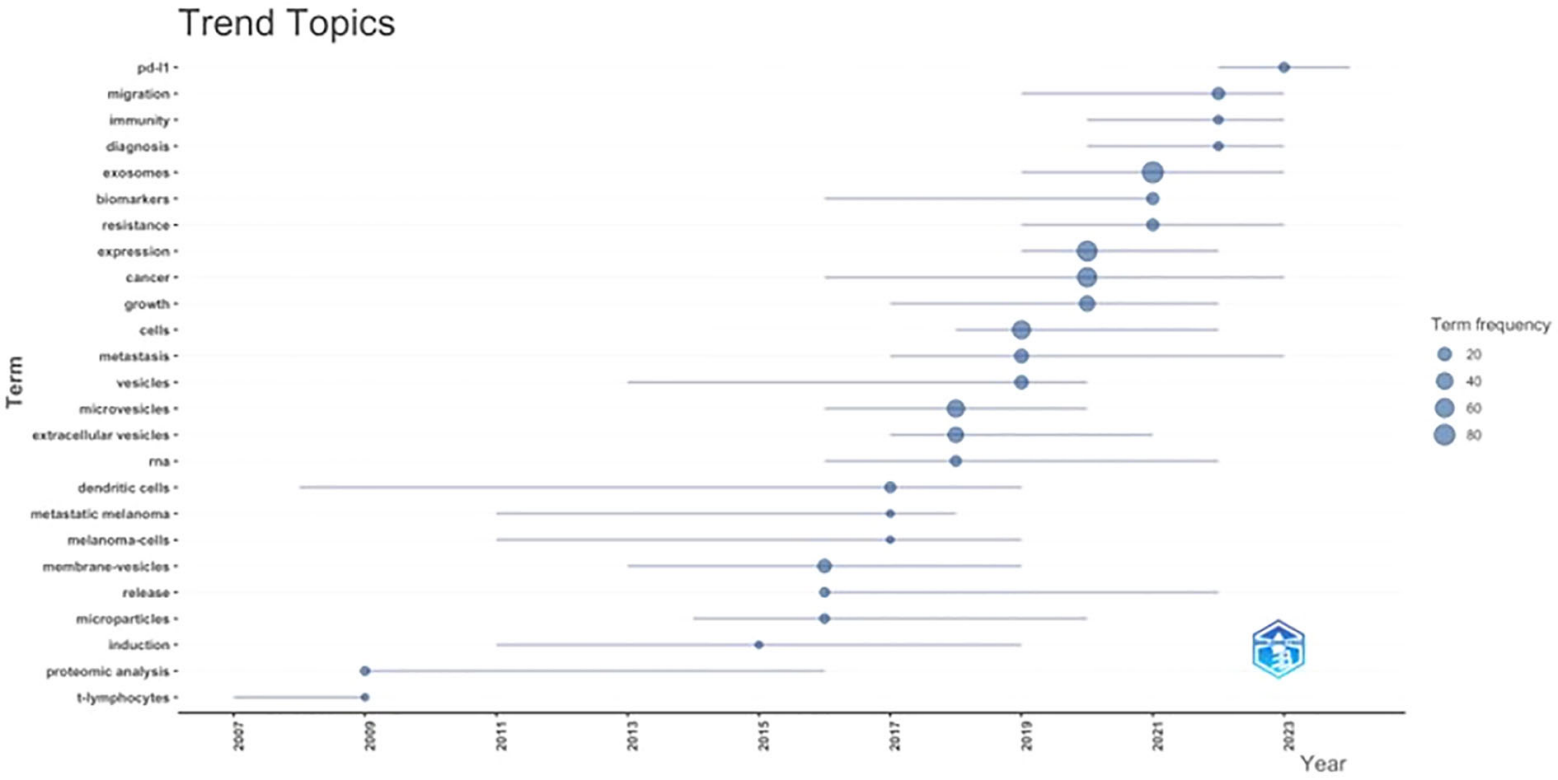
Evolution of trend topics over time. This figure illustrates temporal changes in prominent research topics based on keyword frequency across the study period. Topics are displayed according to the time interval in which they were most active, with bubble size proportional to the number of publications in which each topic appears. The horizontal axis represents time, allowing visualisation of the emergence, persistence, and decline of research themes.

To determine recent trend topics, we assessed the change in keyword frequency over time. Earlier research activity was characterised by a focus on general vesicle-related terms and methodological concepts, including “microparticles,” “release,” and “proteomic analysis.” From approximately 2016 onwards, there is a marked increase in the frequency of biologically and clinically oriented keywords such as “extracellular vesicles,” “metastasis,” “immune response,” “diagnosis,” and “biomarkers.” More recent years show the emergence of translational and immunologically focused topics, including “PD-1,” “migration,” and “immunity,” reflecting a shift towards clinical relevance and therapeutic application within the field. (**Figure 9**).

### 3.7 Analysis of cited references

The most cited reference is the 2012 paper published in *Nature Medicine* by Peinado H (n=111) (20), followed by the paper entitled *“Exosome-mediated transfer of mRNAs and microRNAs is a novel mechanism of genetic exchange between cells”* by Valadi H published in 2007 in *Nature Cell Biology* (n=55) (23). **Figure 6** demonstrates a strong link between the two nodes, representative of a high co-citation frequency. **Table 7** provides a detailed breakdown of the top 10 most cited references in the field of EVs in CM. The most frequently cited references across the included studies were from the *Journal of Extracellular Vesicles* (n = 414) and *Cancer Research* (n = 506).

**Table 7:** Top 10 most cited references.

Cited references	Citations
PEINADO H, 2012, NAT MED, V18, P883, DOI 10.1038/NM.2753	111
VALADI H, 2007, NAT CELL BIOL, V9, P654, DOI 10.1038/NCB1596	55
CHEN G, 2018, NATURE, V560, P382, DOI 10.1038/S41586-018-0392-8	48
HOSHINO A, 2015, NATURE, V527, P329, DOI 10.1038/NATURE15756	47
HOOD JL, 2011, CANCER RES, V71, P3792, DOI 10.1158/0008-5472.CAN-10-4455	42
THÉRY C, 2018, J EXTRACELL VESICLES, V7, DOI 10.1080/20013078.2018.1535750	42
RAPOSO G, 2013, J CELL BIOL, V200, P373, DOI 10.1083/JCB.201211138	41
THERY CLOTILDE, 2006, CURR PROTOC CELL BIOL, VCHAPTER 3, DOI 10.1002/0471143030.CB0322S30	37
VAN NIEL G, 2018, NAT REV MOL CELL BIO, V19, P213, DOI 10.1038/NRM.2017.125	33
KOWAL J, 2016, P NATL ACAD SCI USA, V113, PE968, DOI 10.1073/PNAS.1521230113	32

## 4 Discussion

### 4.1 General overview

In this study, we performed a bibliometric systematic review of research on EVs in CM from 2005 to 2025. Our objective was to present a comprehensive overview of the current research landscape and emerging trends in the field, offering scholars a structured reference framework to understand key findings and identify cutting-edge research frontiers. Our findings indicate a slow, yet steady, rise in citations and publications in the field between 2005 to 2017 with an average of 5 articles published per year. The number of published articles increased significantly between 2018 and 2024 to an average of 32.4 articles published per year. Notably, the United States was the main driving force, with the highest number of annual publication outputs and highest H-index (**Table 3**). It is important to note the geographical imbalance in the field, with China being the only developing country in the top 10 prolific countries in the past 20 years. China also has the lowest number of collaborative studies when compared to the top 5 most productive countries (**Figure 3a**). Greater international collaboration may facilitate closer alignment with activities and consensus recommendations of the International Society for Extracellular Vesicles (ISEV), including Minimal Information for Studies of Extracellular Vesicles (MISEV), thereby supporting methodological consistency and reproducibility within the field. Overall, the predominance of relatively limited link strengths on the map in **Figure 3b** highlights that EV research in cutaneous melanoma remains geographically concentrated and collaborative within select hubs, rather than being supported by broad, global research networks. This pattern may have implications for reproducibility, standardisation, and the translational progression of EV-based biomarkers in melanoma.

When addressing the most prolific institutional affiliations, Chongqing Medical University takes the lead, followed closely by the University of Pittsburgh and the University of Louisville (**Table 4**). Interestingly, when assessing institutional outputs over time, production from Chongqing Medical University increased dramatically between 2021 and 2024, while other prolific institutions showed a more gradual rise in production (**Figure 4**). Notably, the most prolific author group led by Chen Y, is affiliated with Chongqing Medical University, China (**Figure 5**). The fragmented author collaboration structure observed in **Figure 5** suggests that EVs research in CM is frequently conducted within individual research groups, with relatively limited cross-network collaboration. While this pattern does not preclude methodological rigour, broader collaboration may support efforts promoted by the ISEV. Increased multi-institutional engagement could help facilitate consistency in methodology, validation across independent cohorts, and the translational development of EVs-based biomarkers in melanoma.

### 4.2 Reference analysis

By analysing co-cited references, it is possible to identify foundational literature underpinning research in the field. As outlined in **Table 7**, the most frequently cited paper was the landmark original research study by Peinado et al., published in *Nature Medicine*, which described the role of the MET receptor in establishing a pro-metastatic niche through education of bone marrow progenitor cells (20). In keeping with this finding, the citation network analysis in **Figure 6** demonstrates that this publication occupies a central position within the network, acting as a key node linking multiple citation clusters. As review articles were excluded from the analysis, the co-citation patterns shown in **Table 7** and **Figure 6** predominantly reflect the influence of original research studies, highlighting the core experimental work that has shaped the development of EVs research in CM.

### 4.3 Evolution of research hotspots

Keyword analysis is a powerful tool for identifying research hotspots within a field. Analysis of keyword associations and temporal trend topics demonstrates a progressive evolution in extracellular vesicle research in cutaneous melanoma, from early emphasis on vesicle characterisation and methodological concepts towards biologically and clinically oriented themes. Core keywords such as “extracellular vesicles,” “melanoma,” and “exosomes” underpin the literature, while more recent trend topics highlight increasing focus on metastasis, immune modulation, biomarkers, and therapeutic relevance. In this context, Chen Y and colleagues, ranked as the most prolific author group in the field have contributed extensively to work demonstrating how EVs act as vehicles through which melanoma cells acquire metastatic competency (22) The Keywords Plus–based thematic map further supports this progression, identifying foundational EV concepts as basic themes, alongside more developed motor themes related to liquid biopsy, DNA, and immunotherapy. Collectively, these patterns suggest a maturing research landscape increasingly oriented towards translational application, while also highlighting areas where further methodological harmonisation and validation may be required to support clinical integration. These include variability in extracellular vesicle isolation and characterisation practices, reflected by overlapping use of terms such as “exosomes,” “microvesicles,” and “extracellular vesicles,” as well as heterogeneity in analytical approaches applied to biomarker discovery, including proteomic and nucleic acid–based methods. In addition, the emergence of clinically oriented themes such as “liquid biopsy”, “immune modulation”, and immunotherapy-related markers emphasises the importance of validation across independent cohorts and consistent reporting of experimental and clinical outcomes to support reliable translational application in cutaneous melanoma (**Figures 7**–**9**). Integration of the keyword co-occurrence network, thematic map, and temporal trend analyses (**Figures 8**, **9**) indicates that current research frontiers and emerging hotspots in EVs research in CM are concentrated within these interconnected biological and translational domains:

#### 4.3.1 Biomarkers for cutaneous malignant melanoma

EVs have been increasingly recognised as evolutionarily conserved mediators of intercellular communication, playing critical roles in regulating cellular proliferation, migration, organisation, and phenotype across various biological contexts, including development, tissue homeostasis, response to injury, disease progression, and aging (24). Owing to their ability to carry tumour-specific molecular cargo, EVs are emerging as promising non-invasive biomarkers, commonly referred to as “liquid biopsies,” for the detection and monitoring of melanoma (24).

In a notable study, Bollard et al. conducted proteomic and metabolomic profiling of plasma-derived EVs from patients with various melanoma stages and compared them to healthy controls. This group revealed distinct alterations in protein and metabolite content associated with disease stage (25). These findings support the potential of circulating EVs as dynamic biomarkers for melanoma progression. Similarly, Paolino et al. analysed the fatty acid and protein composition of CD81-expressing small EVs (sEVs) across various melanoma stages (0–I, II, III–IV) (26). Their data suggest stage-specific molecular changes occur in circulating CD81^+^ sEVs early in disease progression, underscoring their diagnostic and prognostic value, particularly for early-stage detection, the prediction of metastatic behaviour and screening for recurrence.

#### 4.3.2 EVs as regulators of the pathophysiology and therapeutic landscape of cutaneous malignant melanoma

EVs have emerged as critical regulators in the pathophysiology and therapeutic landscape of CM. These vesicles actively mediate tumour progression by remodelling the tumour microenvironment, promoting angiogenesis, and establishing pre-metastatic niches at distant sites (27). Melanoma-derived EVs facilitate immune evasion by transporting immunosuppressive molecules such as PD-L1, TGF-β, and FasL, which suppress T cell activation and reduce the efficacy of immune checkpoint blockade (9, 21). Moreover, EVs contribute to therapeutic resistance by transferring resistance-associated proteins and regulatory RNAs between tumour and stromal cells, enabling adaptation to targeted and immunotherapies (28). As therapeutic targets, strategies to inhibit EV biogenesis, release, or uptake have shown potential to disrupt these pro-tumorigenic pathways and enhance treatment response (29). EVs are also under investigation as immunotherapeutic tools as dendritic cell-derived EVs carrying melanoma antigens have demonstrated the capacity to stimulate anti-tumour immunity and may serve as cancer vaccine platforms (30). Despite these advances, technical challenges in EVs isolation and characterisation continue to limit widespread clinical application. Nonetheless, the growing body of literature reflects the expanding interest in EVs as both functional mediators and clinical tools in the management of melanoma.

## 5 Limitations

This bibliometric study is subject to several limitations that may have influenced the comprehensiveness of the results. Although a structured and systematic search strategy was applied across the Scopus, WoSCC and EMBASE databases, other widely used sources such as MEDLINE and PubMed were not included. These databases were excluded because they do not provide complete or standardised citation and reference metadata required for bibliometric mapping and network analyses. Consequently, some relevant publications indexed exclusively in MEDLINE or PubMed may not have been captured. Additionally, the analysis was restricted to articles published in English. While English dominates the scientific publishing landscape, this language constraint may have excluded important studies published in other languages. Furthermore, given that citation accumulation typically peaks between one and three years post-publication, recently published high-impact studies or emerging research trends may not yet be adequately represented in the citation metrics. Finally, as a bibliometric study, this analysis is inherently limited in its ability to assess the methodological quality of included studies or to draw causal inferences. Measures such as citation counts, publication volume, and network metrics primarily reflect patterns of research activity and academic influence, rather than the validity or strength of the underlying evidence. Despite these limitations, the current analysis offers valuable insights into the prevailing patterns, influential contributors, and thematic evolution within the field.

## 6 Conclusion

This study represents the first comprehensive bibliometric and visual analysis of research on EVs in CM. Utilising the *Bibliometrix* R-package for quantitative and visual analytics, we demonstrate that scholarly output in this field has grown substantially over the past two decades, with the term “exosomes” emerging as the most extensively studied subtype of EVs. Research output has increased over time, driven by contributions from a limited number of highly productive countries, institutions and authors. However, collaboration analyses at the country, institutional, and author levels reveal a predominantly fragmented research structure, with activity concentrated within select hubs and relatively limited cross-network integration.

Citation and co-citation analyses identify a small number of influential original research studies that have shaped the field, reflecting consolidation around key biological concepts related to tumour progression, metastasis, and microenvironmental interactions. Keyword co-occurrence, Keywords Plus thematic mapping, and trend-topic analyses demonstrate a clear shift from early methodological and descriptive studies towards biologically and clinically oriented themes, including immunotherapy, biomarker development, immune modulation, and liquid biopsy applications.

Collectively, these findings suggest that extracellular vesicle research in cutaneous melanoma is a maturing field with increasing translational intent. Continued efforts to enhance collaboration, methodological consistency, and validation across independent cohorts may support reproducibility and facilitate the clinical integration of EVs-based biomarkers and therapeutic strategies in melanoma.

Overall, this study offers a data-driven perspective on the research dynamics in this field and provides valuable insights for researchers, funding bodies, and policy makers aiming to support and advance innovation in melanoma research.

## Data Availability

The original contributions presented in the study are included in the article/**Supplementary Material**. Further inquiries can be directed to the corresponding authors.

## References

[1] Cancer research UK . What is melanoma skin cancer (2025). Available online at: https://www.cancerresearchuk.org/about-cancer/melanoma/about (Accessed September 13, 2025).

[2] Society AAC . About Melanoma Skin Cancer. Available online at: https://www.cancer.org/cancer/acs-medical-content-and-news-staff.html2024 (Accessed August 25, 2025).

[3] Villanueva J Herlyn M . Melanoma and the tumor microenvironment. Curr Oncol Rep. (2008) 10:439–46. doi: 10.1007/s11912-008-0067-y, PMID: 18706274 PMC5662003

[4] Passarelli A Mannavola F Stucci LS Tucci M Silvestris F . Immune system and melanoma biology: a balance between immunosurveillance and immune escape. Oncotarget. (2017) 8:106132–42. doi: 10.18632/oncotarget.22190, PMID: 29285320 PMC5739707

[5] Suman S Markovic SN . Melanoma-derived mediators can foster the premetastatic niche: crossroad to lymphatic metastasis. Trends Immunol. (2023) 44:724–43. doi: 10.1016/j.it.2023.07.002, PMID: 37573226 PMC10528107

[6] Cheng YC Chang YA Chen YJ Sung HM Bogeski I Su HL . The roles of extracellular vesicles in Malignant melanoma. Cells. (2021) 10. doi: 10.3390/cells10102740, PMID: 34685720 PMC8535053

[7] Welsh JA Goberdhan DCI O’Driscoll L Buzas EI Blenkiron C Bussolati B . Minimal information for studies of extracellular vesicles (MISEV2023): From basic to advanced approaches. J Extracellular Vesicles. (2024) 13. doi: 10.1002/jev2.12404, PMID: 38326288 PMC10850029

[8] Benito-Martin A Galvonas Jasiulionis M Garcia-Silva S . Extracellular vesicles and melanoma: New perspectives on tumour microenvironment and metastasis. Lausanne, Switzerland: Frontiers in Cell and Developmental Biology (2023). 10.3389/fcell.2022.1061982 PMC9871288 36704194

[9] Poggio M Hu T Pai C Fu Q Fong L Blelloch R . Suppression of exosomal PD-L1 induces systemic anti-tumour immunity and memory. Cell. (2019) 144:414–27. doi: 10.1016/j.cell.2019.02.016, PMID: 30951669 PMC6499401

[10] Chen J Song Y Miao F Chen G Zhu YJ Wu N . PDL1-positive exosomes suppress antitumor immunity by inducing tumor-specific CD8^+^ T cell exhaustion during metastasis. Cancer Sci. (2021) 112:3437–54. doi: 10.1111/cas.15033, PMID: 34152672 PMC8409314

[11] García-Silva S Benito-Martín A Sánchez-Redondo S Hernández-Barranco A Ximénez-Embún P Nogués L . Use of extracellular vesicles from lymphatic drainage as surrogate markers of melanoma progression and *BRAF^V600E^* mutation. J Of Exp Med. (2019) 216:1061–70. doi: 10.1084/jem.20181522, PMID: 30975894 PMC6504207

[12] Lunavat TR Cheng L Einarsdottir BO Bagge RO Muralidharan SV Sharples RA . BRAF V600 inhibition alters the microRNA cargo in the vesicular secretome of Malignant melanoma cells. PNAS. (2017). doi: 10.1073/pnas.1705206114, PMID: 28684402 PMC5530690

[13] Donthu N Kumar S Mukherjee D Pandey N Lim WM . How to conduct a bibliometric analysis: An overview and guidelines. J Business Res. (2021) 133:285–96. doi: 10.1016/j.jbusres.2021.04.070

[14] Aria M Cuccurullo C . bibliometrix: An R-tool for comprehensive science mapping analysis. J Informetrics. (2017) 11:959–75. doi: 10.1016/j.joi.2017.08.007

[15] Ouzzani M Hammady H Fedorowicz Z Elmagarmid A . Rayyan: a web and mobile app for systematic reviews. Systematic Rev. (2016). doi: 10.1186/s13643-016-0384-4, PMID: 27919275 PMC5139140

[16] Derviş H . Bibliometric analysis using bibliometrix an R package. J Scientometric Res. (2020) 8:156–60. doi: 10.5530/jscires.8.3.32

[17] Guleria D Gurvinder K . Bibliometric analysis of ecopreneurship using VOSviewer and RStudio Bibliometrix, 1989-2019. Library Hi Tecj. (2021) 39:1001–24. doi: 10.1108/LHT-09-2020-0218

[18] Li R Hu H Luo N Fang J . Bibliometric analysis of publication trends and research hotspots in vagus nerve stimulation: A 20-year panorama. Front Neurol. (2022) 13. doi: 10.3389/fneur.2022.1045763, PMID: 36619909 PMC9811144

[19] Sohrabi C Mathew G Franchi T Kerwan A Griffin M Soleil CDMJ . Impact of the coronavirus (COVID-19) pandemic on scientific research and implications for clinical academic training - A review. Int J Surg. (2021) 86:57–63. doi: 10.1016/j.ijsu.2020.12.008, PMID: 33444873 PMC7833269

[20] Peinado H Aleckovic M Lavotshkin S Matei I Costa-Silva B Moreno-Bueno G . Melanoma exosomes educate bone marrow progenitor cells toward a pro-metastatic phenotype through MET. Nat Med. (2012) 18:883. doi: 10.1038/nm.2753, PMID: 22635005 PMC3645291

[21] Chen G Huang AC Zhang W Zhang G Wu M Xu W . Exosomal PD-L1 contributes to immunosuppression and is associated with anti-PD-1 response. Nature. (2018) 560:382. doi: 10.1038/s41586-018-0392-8, PMID: 30089911 PMC6095740

[22] Chen Y Zhang YH Li J Shi L Xie JC Han X . Novel lncRNA Gm33149 modulates metastatic heterogeneity in melanoma by regulating the miR-5623-3p/Wnt axis via exosomal transfer. Cancer Gene Ther. (2024) 31:364–75. doi: 10.1038/s41417-023-00707-x, PMID: 38072970

[23] Valadi H Ekström K Bossios A Sjöstrand M Lee JJ Lötvall JO . Exosome-mediated transfer of mRNAs and microRNAs is a novel mechanism of genetic exchange between cells. Nat Cell Biol. (2007) 9:654–9. doi: 10.1038/ncb1596, PMID: 17486113

[24] Riazifar M Pone EJ Lotvall J Zhao W . Stem cell extracellular vesicles: extended messages of regeneration. Annu Rev Pharmacol Toxicol. (2017) 57:125–54. doi: 10.1146/annurev-pharmtox-061616-030146, PMID: 27814025 PMC5360275

[25] Bollard SM Howard J Casalou C Kelly BS O’Donnell K Fenn G . Proteomic and metabolomic profiles of plasma-derived Extracellular Vesicles Differentiate melanoma patients from healthy contorls. Trans Oncol. (2024) 50:1936–5233. doi: 10.1016/j.tranon.2024.102152, PMID: 39405606 PMC11736400

[26] Paolino G Huber V Camerini S Casella M Macone A Bertuccini L . The fatty acid and protein profiles of circulating CD81-positive small extracellular vesicles are associated with disease stage in melanoma patients. Cancers. (2021) 13. doi: 10.3390/cancers13164157, PMID: 34439311 PMC8392159

[27] Hood JL Roman SS Wickline SA Hood JL Roman SS Wickline SA . Exosomes released by melanoma cells prepare sentinel lymph nodes for tumor metastasis. Cancer Res. (2011) 71:3792–801. doi: 10.1158/0008-5472.CAN-10-4455, PMID: 21478294

[28] Luan WK Lu X Peng HY Shen XL Rao M Ruan HR . Exosomal miR-19a derived from melanoma cell promotes the vemurafenib resistance of Malignant melanoma through directly targeting LRIG1 to reactivate AKT and MAPK pathway. Pathol Res Pract. (2024) 260. doi: 10.1016/j.prp.2024.155410, PMID: 38955119

[29] Wortzel I Dror S Kenific CM Lyden D . Exosome-mediated metastasis: communication from a distance. Dev Cell. (2019) 49:347–60. doi: 10.1016/j.devcel.2019.04.011, PMID: 31063754

[30] Escudier B Dorval T Chaput N Andre F Caby MP Novault S . Vaccination of metastatic melanoma patients with autologous dendritic cell (DC) derived-exosomes: results of thefirst phase I clinical trial. J Transl Med. (2005) 3:10. doi: 10.1186/1479-5876-3-10, PMID: 15740633 PMC554765

