# Supplementary material for: A bibliometric systematic review of extracellular vesicles in cutaneous malignant melanoma from 2005 to 2025
Source: Front Oncol. 2026 Jan 29;16:1731250. doi: 10.3389/fonc.2026.1731250 (PMC12894022; doi:10.3389/fonc.2026.1731250)
Supplement: Supplementary file 1 [file Supplementaryfile1.docx]

| Title | Journal; Year | Authors | doi | Keywords |
| --- | --- | --- | --- | --- |
| Postlymphadenectomy Analysis of Exosomes from Lymphatic Exudate/Exudative Seroma of Melanoma Patients | MELANOMA: Methods and Protocols; 2021 | GarcÃ­a-Silva, S and XimÃ©nez-EmbÃºn, P and MuÃ±oz, J and Peinado, H and Garcia-Silva, Susana and Ximenez-Embun, Pilar and Munoz, Javier and Peinado, Hector | 10.1007/978-1-0716-1205-7_25 D2 10.1007/978-1-0716-1205-7 | CHAIN-REACTION ASSAY;EXTRACELLULAR VESICLES;NODE DISSECTION;CANCER EXOSOMES;MUTANT KRAS;DNA;CELLS;ULTRAFILTRATION;DRAINAGE;BLOOD |
| RIG-I activation induces the release of extracellular vesicles with antitumor activity | ONCOIMMUNOLOGY; 2016 | Dassler-Plenker, J and Reiners, KS and van den Boorn, JG and Hansen, HP and Putschli, B and Barnert, S and Schuberth-Wagner, C and Schubert, R and TÃ¼ting, T and Hallek, M and Schlee, M and Hartmann, G and von Strandmann, EP and Coch, C and Dassler-Plenker, Juliane and Reiners, Katrin S. and van den Boorn, Jasper G. and Hansen, Hinrich P. and Putschli, Bastian and Barnert, Sabine and Schuberth-Wagner, Christine and Schubert, Rolf and Tueting, Thomas and Hallek, Michael and Schlee, Martin and Hartmann, Gunther and von Strandmann, Elke Pogge and Coch, Christoph | 10.1080/2162402X.2016.1219827 | CELL-DERIVED EXOSOMES;PROMOTE TUMOR-GROWTH;CANCER CELLS;MEMBRANE-VESICLES;DENDRITIC CELLS;NKP30 RECEPTOR;RNA;MICROVESICLES;INNATE;HSP70 |
| Mass Spectrometry-Based Proteomic Characterization of Cutaneous Melanoma Ectosomes Reveals the Presence of Cancer-Related Molecules | INTERNATIONAL JOURNAL OF MOLECULAR SCIENCES; 2020 | Surman, M and Kedracka-Krok, S and Hoja-Lukowicz, D and Jankowska, U and Drozdz, A and Stepien, EL and Przybylo, M and Surman, Magdalena and Kedracka-Krok, Sylwia and Hoja-Lukowicz, Dorota and Jankowska, Urszula and Drozdz, Anna and Stepien, Ewa L. and Przybylo, Malgorzata | 10.3390/ijms21082934 | TUMOR-DERIVED MICROVESICLES;VERTICAL GROWTH-PHASE;EXTRACELLULAR VESICLES;CELLS;INTEGRIN;PROMOTE;EXPRESSION;MICROPARTICLES;FIBROBLASTS;MELANOCYTES |
| The capture of extracellular vesicles endogenously released by xenotransplanted tumours induces an inflammatory reaction in the premetastatic niche | JOURNAL OF EXTRACELLULAR VESICLES; 2023 | Blavier, L and Nakata, R and Neviani, P and Sharma, K and Shimada, H and Benedicto, A and Matei, I and Lyden, D and DeClerck, YA and Blavier, Laurence and Nakata, Rie and Neviani, Paolo and Sharma, Khounish and Shimada, Hiroyuki and Benedicto, Aitor and Matei, Irina and Lyden, David and DeClerck, Yves A. | 10.1002/jev2.12326 | CANCER METASTASIS;EXOSOMES;CELLS;MECHANISMS;INITIATE;REVEALS;ROLES;LUNG |
| Cancer cells copy migratory behavior and exchange signaling networks via extracellular vesicles | EMBO JOURNAL; 2018 | Steenbeek, SC and Pham, TV and de Ligt, J and Zomer, A and Knol, JC and Piersma, SR and Schelfhorst, T and Huisjes, R and Schiffelers, RM and Cuppen, E and Jimenez, CR and van Rheenen, J and Steenbeek, Sander C. and Pham, Thang V. and de Ligt, Joep and Zomer, Anoek and Knol, Jaco C. and Piersma, Sander R. and Schelfhorst, Tim and Huisjes, Rick and Schiffelers, Raymond M. and Cuppen, Edwin and Jimenez, Connie R. and van Rheenen, Jacco | 10.15252/embj.201798357 | METASTATIC NICHE FORMATION;INTERCELLULAR TRANSFER;TUMOR HETEROGENEITY;EXOSOMES;MICROENVIRONMENT;MODEL;IDENTIFICATION;MICROVESICLES;VISUALIZATION;COMMUNICATION |
| Targeting CSPG4 for isolation of melanoma cell-derived exosomes from body fluids | HNO; 2020 | Ferrone, S and Whiteside, TL and Ferrone, S. and Whiteside, T. L. | 10.1007/s00106-019-00811-1 | ANTIGEN HMW-MAA;T-LYMPHOCYTES;CANCER;IMMUNOTHERAPY;ANTIBODY;PROLONGATION;EXPRESSION;APOPTOSIS;PERICYTES;SURVIVAL |
| A new ALK isoform transported by extracellular vesicles confers drug resistance to melanoma cells | MOLECULAR CANCER; 2018 | Cesi, G and Philippidou, D and Kozar, I and Kim, YJ and Bernardin, F and Van Niel, G and Wienecke-Baldacchino, A and Felten, P and Letellier, E and Dengler, S and Nashan, D and Haan, C and Kreis, S and Cesi, Giulia and Philippidou, Demetra and Kozar, Ines and Kim, Yeoun Jin and Bernardin, Francois and Van Niel, Guillaume and Wienecke-Baldacchino, Anke and Felten, Paul and Letellier, Elisabeth and Dengler, Sonja and Nashan, Dorothee and Haan, Claude and Kreis, Stephanie | 10.1186/s12943-018-0886-x | METASTATIC MELANOMA;INTERCELLULAR TRANSFER;CANCER-IMMUNOTHERAPY;PROGENITOR CELLS;RAF INHIBITORS;BRAF;MUTATIONS;RECEPTOR;THERAPY;KINASE |
| Extracellular vesicles released from ganglioside GD2-expressing melanoma cells enhance the malignant properties of GD2-negative melanomas | SCIENTIFIC REPORTS; 2023 | Yesmin, F and Furukawa, K and Kambe, M and Ohmi, Y and Bhuiyan, RH and Hasnat, MA and Mizutani, M and Tajima, O and Hashimoto, N and Tsuchida, A and Kaneko, K and Furukawa, K and Yesmin, Farhana and Furukawa, Keiko and Kambe, Mariko and Ohmi, Yuhsuke and Bhuiyan, Robiul Hasan and Hasnat, Mohammad Abul and Mizutani, Momoka and Tajima, Orie and Hashimoto, Noboru and Tsuchida, Akiko and Kaneko, Kei and Furukawa, Koichi | 10.1038/s41598-023-31216-4 | G(D3) SYNTHASE GENE;MONOCLONAL-ANTIBODIES;EXPRESSION CLONING;GD2 GANGLIOSIDE;STEM-CELLS;EXOSOMES;ACTIVATION;GROWTH;LINES;GLYCOSPHINGOLIPIDS |
| Application of high-mannose-type glycan-specific lectin from <i>Oscillatoria Agardhii</i> for affinity isolation of tumor-derived extracellular vesicles | ANALYTICAL BIOCHEMISTRY; 2019 | Yamamoto, M and Harada, Y and Suzuki, T and Fukushige, T and Yamakuchi, M and Kanekura, T and Dohmae, N and Hori, K and Maruyama, I and Yamamoto, Mika and Harada, Yoichiro and Suzuki, Takehiro and Fukushige, Tomoko and Yamakuchi, Munekazu and Kanekura, Takuro and Dohmae, Naoshi and Hori, Kanji and Maruyama, Ikuro | 10.1016/j.ab.2019.06.001 | CANCER EXOSOMES;GLYCOSYLATION;GLYCOME;CYANOBACTERIUM;IDENTIFICATION;DIAGNOSIS;REVEALS;CELLS;CD109 |
| Extracellular vesicles from cancer cell lines of different origins drive the phenotype of normal oral fibroblasts in a CAF-like direction | FRONTIERS IN ONCOLOGY; 2024 | Soland, TM and Lipka, A and Ruus, AK and MolvÃ¦rsmyr, AK and Galtung, HK and Haug, TM and Soland, Tine M. and Lipka, Aleksandra and Ruus, Ann-Kristin and Molvaersmyr, Ann-Kristin and Galtung, Hilde K. and Haug, Trude M. | 10.3389/fonc.2024.1456346 | ION CHANNELS;ANO1 |
| Subpopulations of extracellular vesicles from human metastatic melanoma tissue identified by quantitative proteomics after optimized isolation | JOURNAL OF EXTRACELLULAR VESICLES; 2020 | Crescitelli, R and LÃ¤sser, C and Jang, SC and Cvjetkovic, A and MalmhÃ¤ll, C and Karimi, N and Haag, JL and Johansson, I and Fuchs, J and Thorsell, A and Gho, YS and Bagge, RO and Latvall, J and Crescitelli, Rossella and Lasser, Cecilia and Jang, Su Chul and Cvjetkovic, Aleksander and Malmhall, Carina and Karimi, Nasibeh and Hoog, Johanna L. and Johansson, Iva and Fuchs, Johannes and Thorsell, Annika and Gho, Yong Song and Bagge, R. Olofsson and Lotvall, Jan | 10.1080/20013078.2020.1722433 | TRANSFERRIN RECEPTOR;MALIGNANT-MELANOMA;MEMBRANE-VESICLES;EXOSOMES;CELLS;MARKERS;RME-8;VISUALIZATION;ENDOCYTOSIS;MICRORNAS |
| Melanoma-Derived Extracellular Vesicles Induce CD36-Mediated Pre-Metastatic Niche | BIOMOLECULES; 2024 | Suman, S and Nevala, WK and Leontovich, AA and Ward, C and Jakub, JW and Kim, Y and Geng, LY and Stueven, NA and Atherton, CL and Moore, RM and Schimke, JM and Lucien-Matteoni, F and McLaughlin, SA and Markovic, SN and Suman, Shankar and Nevala, Wendy K. and Leontovich, Alexey A. and Ward, Caitlin and Jakub, James W. and Kim, Yohan and Geng, Liyi and Stueven, Noah A. and Atherton, Chathu L. and Moore, Raymond M. and Schimke, Jill M. and Lucien-Matteoni, Fabrice and McLaughlin, Sarah A. and Markovic, Svetomir N. | 10.3390/biom14070837 | OXIDIZED LDL;CD36;MACROPHAGES;CELLS |
| Anti-Metastatic Function of Extracellular Vesicles Derived from <i>Nanog</i>-Overexpressing Melanoma | CURRENT ONCOLOGY; 2022 | Hatakenaka, T and Matsuki, N and Minagawa, S and Khoo, CSM and Saito, M and Hatakenaka, Tomohiro and Matsuki, Nahoko and Minagawa, Seiya and Khoo, Celine Swee May and Saito, Mikako | 10.3390/curroncol29020088 | EXOSOMES |
| Reprogramming of T cell-derived small extracellular vesicles using IL2 surface engineering induces potent anti-cancer effects through miRNA delivery | JOURNAL OF EXTRACELLULAR VESICLES; 2022 | Jung, DKY and Shin, S and Kang, SM and Jung, IS and Ryu, S and Noh, S and Choi, SJ and Jeong, J and Lee, BY and Kim, KS and Kim, CS and Yoon, JH and Lee, CH and Bucher, F and Kim, YN and Im, SH and Song, BJ and Yea, K and Baek, MC and Jung, Dokyung and Shin, Sanghee and Kang, Sung-Min and Jung, Inseong and Ryu, Suyeon and Noh, Soojeong and Choi, Sung-Jin and Jeong, Jongwon and Lee, Beom Yong and Kim, Kwang-Soo and Kim, Christine Seulki and Yoon, Jong Hyuk and Lee, Chan-Hyeong and Bucher, Felicitas and Kim, Yong-Nyun and Im, Sin-Hyeog and Song, Byoung-Joon and Yea, Kyungmoo and Baek, Moon-Chang | 10.1002/jev2.12287 | EXOSOMES;MELANOMA;INTERLEUKIN-2;DACARBAZINE;BIOGENESIS;ACTIVATION;RECEPTORS;IMMUNITY;GROWTH |
| Circulating miRNAs in Small Extracellular Vesicles Secreted by a Human Melanoma Xenograft in Mouse Brains | CANCERS; 2020 | Guglielmi, L and Nardella, M and Musa, C and Cifola, I and Porru, M and Cardinali, B and Iannetti, I and Di Pietro, C and Bolasco, G and Palmieri, V and Vilardo, L and Panini, N and Bonaventura, F and Papi, M and Scavizzi, F and Raspa, M and Leonetti, C and Falcone, G and Felsani, A and D'Agnano, I and Guglielmi, Loredana and Nardella, Marta and Musa, Carla and Cifola, Ingrid and Porru, Manuela and Cardinali, Beatrice and Iannetti, Ilaria and Di Pietro, Chiara and Bolasco, Giulia and Palmieri, Valentina and Vilardo, Laura and Panini, Nicolo and Bonaventura, Fabrizio and Papi, Massimiliano and Scavizzi, Ferdinando and Raspa, Marcello and Leonetti, Carlo and Falcone, Germana and Felsani, Armando and D'Agnano, Igea | 10.3390/cancers12061635 | INTERCELLULAR-COMMUNICATION;MALIGNANT-MELANOMA;TUMOR-GROWTH;CELL;MICRORNAS;CANCER;EXOSOMES;PROLIFERATION;INHIBITION;EXPRESSION |
| Tumour-associated antigenic peptides are present in the HLA class I ligandome of cancer cell line derived extracellular vesicles | IMMUNOLOGY; 2022 | Kumar, P and Boyne, C and Brown, S and Qureshi, A and Thorpe, P and Synowsky, SA and Shirran, S and Powis, SJ and Kumar, Pankaj and Boyne, Caitlin and Brown, Sydney and Qureshi, Ayesha and Thorpe, Peter and Synowsky, Silvia A. and Shirran, Sally and Powis, Simon J. | 10.1111/imm.13471 EA APR 2022 | CLINICAL-TRIAL;EXOSOMES;IDENTIFICATION;VACCINE;MUC1 |
| Impact of Experimental Conditions on Extracellular Vesicles' Proteome: A Comparative Study | LIFE-BASEL; 2023 | BÃ¶rÃ¶czky, T and Dobra, G and Bukva, M and Gyukity-SebestyÃ©n, E and Hunyadi-GulyÃ¡s, E and Darula, Z and HorvÃ¡th, P and BuzÃ¡s, K and Harmati, M and Boroczky, Timea and Dobra, Gabriella and Bukva, Matyas and Gyukity-Sebestyen, Edina and Hunyadi-Gulyas, Eva and Darula, Zsuzsanna and Horvath, Peter and Buzas, Krisztina and Harmati, Maria | 10.3390/life13010206 | |
| Human Melanoma-Derived Extracellular Vesicles Regulate Dendritic Cell Maturation | FRONTIERS IN IMMUNOLOGY; 2017 | Maus, RLG and Jakub, JW and Nevala, WK and Christensen, TA and Noble-Orcutt, K and Sachs, Z and Hieken, TJ and Markovic, SN and Maus, Rachel L. G. and Jakub, James W. and Nevala, Wendy K. and Christensen, Trace A. and Noble-Orcutt, Klara and Sachs, Zohar and Hieken, Tina J. and Markovic, Svetomir N. | 10.3389/fimmu.2017.00358 | ANTIGEN PRESENTATION;STATISTICAL-MODEL;LYMPH-NODES;IN-VITRO;EXOSOMES;DIFFERENTIATION;PROTEIN;S100A9;LYMPHOCYTES;METASTASIS |
| An Immunocapture-Based Assay for Detecting Multiple Antigens in Melanoma-Derived Extracellular Vesicles | MELANOMA: Methods and Protocols; 2021 | Campos-Silva, C and CÃ¡ceres-Martell, Y and LÃ³pez-Cobo, S and Rodriguez, MJ and Jara, R and YÃ¡Ã±ez-MÃ³, M and Vales-Gomez, M and Campos-Silva, Carmen and Caceres-Martell, Yaiza and Lopez-Cobo, Sheila and Rodriguez, Maria Josefa and Jara, Ricardo and Yanez-Mo, Maria and Vales-Gomez, Mar | 10.1007/978-1-0716-1205-7_24 D2 10.1007/978-1-0716-1205-7 | CELLS |
| Extracellular lipidosomes containing lipid droplets and mitochondria are released during melanoma cell division | CELL COMMUNICATION AND SIGNALING; 2024 | KarbanovÃ¡, J and Deniz, IA and Wilsch-BrÃ¤uninger, M and Couto, RAD and Fargeas, CA and Santos, MF and Lorico, A and Corbeil, D and Karbanova, Jana and Deniz, Ilker A. and Wilsch-Braeuninger, Michaela and Couto, Rita Alexandra de Sousa and Fargeas, Christine A. and Santos, Mark F. and Lorico, Aurelio and Corbeil, Denis | 10.1186/s12964-024-01471-7 | CANCER STEM-CELLS;MALIGNANT-MELANOMA;MEMBRANE-VESICLES;NEURAL PROGENITORS;CD133 PROMININ-1;MARKER;PROTEIN;EXPRESSION;NESTIN;TUMORIGENICITY |
| Isolation and detection of extracellular vesicles from melanoma cells and liquid biopsies using size-exclusion chromatography and nano-flow cytometry | STAR PROTOCOLS; 2023 | Lattmann, E and Lapaire, V and Levesque, MP and Lattmann, Evelyn and Lapaire, Valerie and Levesque, Mitchell P. | 10.1016/j.xpro.2023.102365 EA JUL 2023 | |
| Exosome-dependent immune surveillance at the metastatic niche requires BAG6 and CBP/p300-dependent acetylation of p53 | THERANOSTICS; 2019 | Schuldner, M and DÃ¶rsam, B and Shatnyeva, O and Reiners, KS and Kubarenko, A and Hansen, HP and Finkernagel, F and Roth, K and Theurich, S and Nist, A and Stiewe, T and Paschen, A and Knittel, G and Reinhardt, HC and MÃ¼ller, R and Hallek, M and von Strandmann, EP and Schuldner, Maximiliane and Doersam, Bastian and Shatnyeva, Olga and Reiners, Katrin S. and Kubarenko, Andriy and Hansen, Hinrich P. and Finkernagel, Florian and Roth, Katrin and Theurich, Sebastian and Nist, Andrea and Stiewe, Thorsten and Paschen, Annette and Knittel, Gero and Reinhardt, Hans C. and Mueller, Rolf and Hallek, Michael and von Strandmann, Elke Pogge | 10.7150/thno.36378 | B-ASSOCIATED TRANSCRIPT-3;MELANOMA PROGRESSION;EXTRACELLULAR VESICLES;NKP30 RECEPTOR;CELLS;EXPRESSION;RESPONSES;COMMUNICATION;BIOGENESIS;AUTOPHAGY |
| Fourier-Transform InfraRed (FT-IR) spectroscopy to show alterations in molecular composition of EV subpopulations from melanoma cell lines in different malignancy | BIOCHEMISTRY AND BIOPHYSICS REPORTS | Stepien, EL and Kaminska, A and Surman, M and Karbowska, D and WrÃ³bel, A and Przybylo, M and Stepien, Ewa L. and Kaminska, Agnieszka and Surman, Magdalena and Karbowska, Dagmara and Wrobel, Andrzej and Przybylo, Malgorzata | 10.1016/j.bbrep.2020.100888 EA JAN 2021 | EXPRESSION;PROTEINS;DISORDER;UVEAL;P53 |
| An Integrated Microfluidic-SERS Platform Enables Sensitive Phenotyping of Serum Extracellular Vesicles in Early Stage Melanomas | ADVANCED FUNCTIONAL MATERIALS; 2022 | Wang, J and Kao, YC and Zhou, Q and Wuethrich, A and Stark, MS and Schaider, H and Soyer, HP and Lin, LLL and Trau, M and Wang, Jing and Kao, Yung-Ching and Zhou, Quan and Wuethrich, Alain and Stark, Mitchell S. and Schaider, Helmut and Soyer, H. Peter and Lin, Lynlee L. and Trau, Matt | 10.1002/adfm.202010296 EA JUL 2021 | EXPRESSION;DIAGNOSIS;EXOSOMES;IMMUNOASSAY;PROGRESSION;MCAM/MUC18;DERMOSCOPY;LESIONS |
| Melanoma-derived extracellular vesicles mediate lymphatic remodelling and impair tumour immunity in draining lymph nodes | JOURNAL OF EXTRACELLULAR VESICLES; 2022 | Leary, N and Walser, S and He, YL and Cousin, N and Pereira, P and Gallo, A and Collado-Diaz, V and Halin, C and Garcia-Silva, S and Peinado, H and Dieterich, LC and Leary, Noelle and Walser, Sarina and He, Yuliang and Cousin, Nikola and Pereira, Paulo and Gallo, Alessandro and Collado-Diaz, Victor and Halin, Cornelia and Garcia-Silva, Susana and Peinado, Hector and Dieterich, Lothar C. | 10.1002/jev2.12197 | RNA-SEQ;NICHE FORMATION;CELLS;LYMPHANGIOGENESIS;EXPRESSION;EXOSOMES;MACROPHAGES;MECHANISMS;TOLERANCE;PD-L1 |
| Circulating extracellular vesicles are monitoring biomarkers of anti-PD1 response and enhancer of tumor progression and immunosuppression in metastatic melanoma | JOURNAL OF EXPERIMENTAL & CLINICAL CANCER RESEARCH; 2023 | Serrati, S and Di Fonte, R and Porcelli, L and De Summa, S and De Risi, I and Fucci, L and Ruggieri, E and Marvulli, TM and Strippoli, S and Fasano, R and Rafaschieri, T and Guida, G and Guida, M and Azzariti, A and Serrati, Simona and Di Fonte, Roberta and Porcelli, Letizia and De Summa, Simona and De Risi, Ivana and Fucci, Livia and Ruggieri, Eustachio and Marvulli, Tommaso Maria and Strippoli, Sabino and Fasano, Rossella and Rafaschieri, Tania and Guida, Gabriella and Guida, Michele and Azzariti, Amalia | 10.1186/s13046-023-02808-9 | NICHE FORMATION |
| Tumor-derived extracellular vesicles regulate tumor-infiltrating regulatory T cells via the inhibitory immunoreceptor CD300a | ELIFE; 2021 | Nakazawa, Y and Nishiyama, N and Koizumi, H and Kanemaru, K and Nakahashi-Oda, C and Shibuya, A and Nakazawa, Yuta and Nishiyama, Nanako and Koizumi, Hitoshi and Kanemaru, Kazumasa and Nakahashi-Oda, Chigusa and Shibuya, Akira | 10.7554/eLife.61999; 10.7554/eLife.61999.sa0; 10.7554/eLife.61999.sa1; 10.7554/eLife.61999.sa2 | I INTERFERONS;MAST-CELL;CANCER;RESPONSES;PHOSPHATIDYLSERINE;MICRORNAS;APOPTOSIS;EXOSOMES;GROWTH;TLR3 |
| BRAF<SUP>V600</SUP> inhibition alters the microRNA cargo in the vesicular secretome of malignant melanoma cells | PROCEEDINGS OF THE NATIONAL ACADEMY OF SCIENCES OF THE UNITED STATES OF AMERICA; 2017 | Lunavat, TR and Cheng, L and Einarsdottir, BO and Bagge, RO and Muralidharan, SV and Sharples, RA and LÃ¤sser, C and Gho, YS and Hill, AF and Nilsson, JA and LÃ¶tvall, J and Lunavat, Taral R. and Cheng, Lesley and Einarsdottir, Berglind O. and Bagge, Roger Olofsson and Muralidharan, Somsundar Veppil and Sharples, Robyn A. and Lasser, Cecilia and Gho, Yong Song and Hill, Andrew F. and Nilsson, Jonas A. and Lotvall, Jan | 10.1073/pnas.1705206114 | TRANSCRIPTION FACTOR;SMALL RNAS;EXOSOMES;SUBSETS;REVEALS;KINASE;POTENT;MITF;GENE;BRAF |
| Inhibition of cholesterol transport impairs Cav-1 trafficking and small extracellular vesicles secretion, promoting amphisome formation in melanoma cells | TRAFFIC; 2023 | Peruzzu, D and Boussadia, Z and Fratini, F and Spadaro, F and Bertuccini, L and Sanchez, M and Carollo, M and Matarrese, P and Falchi, M and Iosi, F and Raggi, C and Parolini, I and CarÃ¨, A and Sargiacomo, M and Gagliardi, MC and Fecchi, K and Peruzzu, Daniela and Boussadia, Zaira and Fratini, Federica and Spadaro, Francesca and Bertuccini, Lucia and Sanchez, Massimo and Carollo, Maria and Matarrese, Paola and Falchi, Mario and Iosi, Francesca and Raggi, Carla and Parolini, Isabella and Care, Alessandra and Sargiacomo, Massimo and Gagliardi, Maria Cristina and Fecchi, Katia | 10.1111/tra.12878 EA JAN 2023 | IN-VIVO;CAVEOLIN-1;DYNAMICS;EXOSOMES;MITOCHONDRIA;BIOGENESIS;MIGRATION;DISEASE |
| Melanoma exosomes deliver a complex biological payload that upregulates PTPN11 to suppress T lymphocyte function | PIGMENT CELL & MELANOMA RESEARCH; 2017 | Wu, YT and Deng, WT and McGinley, EC and Klinke, DJ and Wu, Yueting and Deng, Wentao and McGinley, Emily Chambers and Klinke, David J., II | 10.1111/pcmr.12564 | EXTRACELLULAR VESICLES;INTERFERON-GAMMA;MESSENGER-RNA;HUMAN-CELLS;MICROVESICLES;INTERLEUKIN-12;SHP-2;B16;COMMUNICATION;MECHANISMS |
| Inactivation of EMILIN-1 by Proteolysis and Secretion in Small Extracellular Vesicles Favors Melanoma Progression and Metastasis | INTERNATIONAL JOURNAL OF MOLECULAR SCIENCES; 2021 | LÃ³pez, AA and Mazariegos, MS and Capuano, A and XimÃ©nez-EmbÃºn, P and Hergueta-Redondo, M and Recio, JA and MuÃ±oz, E and Al-Shahrour, F and MuÃ±oz, J and MegÃ­as, D and Doliana, R and Spessotto, P and Peinado, H and Amor Lopez, Ana and Mazariegos, Marina S. and Capuano, Alessandra and Ximenez-Embun, Pilar and Hergueta-Redondo, Marta and Angel Recio, Juan and Munoz, Eva and Al-Shahrour, Fatima and Munoz, Javier and Megias, Diego and Doliana, Roberto and Spessotto, Paola and Peinado, Hector | 10.3390/ijms22147406 | BREAST-CANCER;TUMOR;EXOSOMES;GENE;EXPRESSION;CELLS;NODE;METALLOPROTEINASES;BIOGENESIS |
| Adipocyte Extracellular Vesicles Decrease p16<SUP>INK4A</SUP> in Melanoma: An Additional Link between Obesity and Cancer | JOURNAL OF INVESTIGATIVE DERMATOLOGY; 2022 | Lazar, I and Clement, E and CarriÃ©, L and Esteve, D and Dauvillier, S and Moutahir, M and Dalle, S and Delmas, V and Andrieu-Abadie, N and Larue, L and Muller, C and Nieto, L and Lazar, Ikrame and Clement, Emily and Carrie, Lorry and Esteve, David and Dauvillier, Stephanie and Moutahir, Mohamed and Dalle, Stephane and Delmas, Veronique and Andrieu-Abadie, Nathalie and Larue, Lionel and Muller, Catherine and Nieto, Laurence | 10.1016/j.jid.2022.01.026 EA AUG 2022 | FATTY-ACID OXIDATION;MALIGNANT-MELANOMA;METASTATIC MELANOMA;MELANOCYTIC LESIONS;TUMOR PROGRESSION;CELL;EXPRESSION;SUSCEPTIBILITY;METAANALYSIS;MUTATIONS |
| Extracellular vesicles derived from melanoma cells induce carcinoma-associated fibroblasts via miR-92b-3p mediated downregulation of PTEN | JOURNAL OF EXTRACELLULAR VESICLES; 2024 | Kewitz-Hempel, S and Windisch, N and Hause, G and Mueller, L and Sunderkoetter, C and Gerloff, D and Kewitz-Hempel, Stefanie and Windisch, Nicola and Hause, Gerd and Mueller, Lutz and Sunderkoetter, Cord and Gerloff, Dennis | 10.1002/jev2.12509 | CANCER-ASSOCIATED FIBROBLASTS;EXOSOMES;MACROPHAGE;GROWTH;CONTRIBUTES;GENE |
| Proteomic and metabolomic profiles of plasma-derived Extracellular Vesicles differentiate melanoma patients from healthy controls | TRANSLATIONAL ONCOLOGY; 2024 | Bollard, SM and Howard, J and Casalou, C and Kelly, BS and O'Donnell, K and Fenn, G and O'Reilly, J and Milling, R and Shields, M and Wilson, M and Ajaykumar, A and Triana, K and Wynne, K and Tobin, DJ and Kelly, PA and Mccann, A and Potter, SM and Bollard, S. M. and Howard, J. and Casalou, C. and Kelly, B. S. and O'Donnell, K. and Fenn, G. and O'Reilly, J. and Milling, R. and Shields, M. and Wilson, M. and Ajaykumar, A. and Triana, K. and Wynne, K. and Tobin, D. J. and Kelly, P. A. and Mccann, A. and Potter, S. M. | 10.1016/j.tranon.2024.102152 EA OCT 2024 | THIN MELANOMAS;TENASCIN-C;CANCER;EXOSOMES;PROGNOSIS;REVEALS |
| Glycan Node Analysis Detects Varying Glycosaminoglycan Levels in Melanoma-Derived Extracellular Vesicles | INTERNATIONAL JOURNAL OF MOLECULAR SCIENCES; 2023 | Goncalves, JP and Walker, SA and de LeÃ³n, JSAD and Yang, YB and Davidovich, I and Busatto, S and Sarkaria, J and Talmon, Y and Borges, CR and Wolfram, J and Goncalves, Jenifer Pendiuk and Walker, Sierra A. and de Leon, Jesus S. Aguilar Diaz and Yang, Yubo and Davidovich, Irina and Busatto, Sara and Sarkaria, Jann and Talmon, Yeshayahu and Borges, Chad R. and Wolfram, Joy | 10.3390/ijms24108506 | HYALURONAN |
| An Interferon-Driven Oxysterol-Based Defense against Tumor-Derived Extracellular Vesicles | CANCER CELL; 2019 | Ortiz, A and Gui, J and Zahedi, F and Yu, PF and Cho, C and Bhattacharya, S and Carbone, CJ and Yu, QJ and Katlinski, KV and Katlinskaya, YV and Handa, S and Haas, V and Volk, SW and Brice, AK and Wals, K and Matheson, NJ and Antrobus, R and Ludwig, S and Whiteside, TL and Sander, C and Tarhini, AA and Kirkwood, JM and Lehner, PJ and Guo, W and Rui, H and Minn, AJ and Koumenis, C and Diehl, JA and Fuchs, SY and Ortiz, Angelica and Gui, Jun and Zahedi, Farima and Yu, Pengfei and Cho, Christina and Bhattacharya, Sabyasachi and Carbone, Christopher J. and Yu, Qiujing and Katlinski, Kanstantsin, V and Katlinskaya, Yuliya, V and Handa, Simran and Haas, Victor and Volk, Susan W. and Brice, Angela K. and Wals, Kim and Matheson, Nicholas J. and Antrobus, Robin and Ludwig, Sonja and Whiteside, Theresa L. and Sander, Cindy and Tarhini, Ahmad A. and Kirkwood, John M. and Lehner, Paul J. and Guo, Wei and Rui, Hallgeir and Minn, Andy J. and Koumenis, Constantinos and Diehl, J. Alan and Fuchs, Serge Y. | 10.1016/j.ccell.2018.12.001 | METASTATIC NICHE FORMATION;IFNAR1 CHAIN;EXOSOMES;EXPRESSION;MELANOMA;RECEPTOR;UBIQUITINATION;DEGRADATION;RESERPINE;INITIATE |
| Investigating the In Vivo Biodistribution of Extracellular Vesicles Isolated from Various Human Cell Sources Using Positron Emission Tomography | MOLECULAR PHARMACEUTICS; 2024 | Rosenkrans, ZT and Thickens, AS and Kink, JA and Aluicio-Sarduy, E and Engle, JW and Hematti, P and Hernandez, R and Rosenkrans, Zachary T. and Thickens, Anna S. and Kink, John A. and Aluicio-Sarduy, Eduardo and Engle, Jonathan W. and Hematti, Peiman and Hernandez, Reinier | 10.1021/acs.molpharmaceut.4c00298 EA AUG 2024 | TARGETED DELIVERY;EXOSOMES;MICE |
| miR-214-Enriched Extracellular Vesicles Released by Acid-Adapted Melanoma Cells Promote Inflammatory Macrophage-Dependent Tumor Trans-Endothelial Migration | CANCERS; 2022 | Andreucci, E and Ruzzolini, J and Bianchini, F and Versienti, G and Biagioni, A and Lulli, M and Guasti, D and Nardini, P and SerratÃ¬, S and Margheri, F and Laurenzana, A and Nediani, C and Peppicelli, S and Calorini, L and Andreucci, Elena and Ruzzolini, Jessica and Bianchini, Francesca and Versienti, Giampaolo and Biagioni, Alessio and Lulli, Matteo and Guasti, Daniele and Nardini, Patrizia and Serrati, Simona and Margheri, Francesca and Laurenzana, Anna and Nediani, Chiara and Peppicelli, Silvia and Calorini, Lido | 10.3390/cancers14205090 | CANCER;PROGRESSION;MIR-214;AGGRESSIVENESS;EXPRESSION |
| Proteomic and Biochemical Analysis of Extracellular Vesicles Isolated from Blood Serum of Patients with Melanoma | SEPARATIONS; 2022 | Kurg, K and Planken, A and Kurg, R and Kurg, Kristiina and Planken, Anu and Kurg, Reet | 10.3390/separations9040086 | TUMOR-MARKERS;TRANSFERRIN;GALECTIN-3;EXOSOMES;RECEPTOR;PROTEIN |
| Adipocyte extracellular vesicles carry enzymes and fatty acids that stimulate mitochondrial metabolism and remodeling in tumor cells | EMBO JOURNAL; 2020 | Clement, E and Lazar, I and AttanÃ©, C and CarriÃ©, L and Dauvillier, S and Ducoux-Petit, M and Esteve, D and Menneteau, T and Moutahir, M and Le Gonidec, S and Dalle, S and Valet, P and Burlet-Schiltz, O and Muller, C and Nieto, L and Clement, Emily and Lazar, Ikrame and Attane, Camille and Carrie, Lorry and Dauvillier, Stephanie and Ducoux-Petit, Manuelle and Esteve, David and Menneteau, Thomas and Moutahir, Mohamed and Le Gonidec, Sophie and Dalle, Stephane and Valet, Philippe and Burlet-Schiltz, Odile and Muller, Catherine and Nieto, Laurence | 10.15252/embj.2019102525 EA JAN 2020 | BODY-MASS INDEX;LYMPH-NODE METASTASIS;MALIGNANT-MELANOMA;ADIPOSE-TISSUE;INCREASED RISK;CANCER;OBESITY;LIPOLYSIS;TRAFFICKING;OXIDATION |
| Cancer cell line-specific protein profiles in extracellular vesicles identified by proteomics | PLOS ONE; 2020 | Guerreiro, EM and Ovstebo, R and Thiede, B and Costea, DE and Soland, TM and Galtung, HK and Guerreiro, Eduarda M. and Ovstebo, Reidun and Thiede, Bernd and Costea, Daniela Elena and Soland, Tine M. and Galtung, Hilde Kanli | 10.1371/journal.pone.0238591 | LYMPH-NODE METASTASIS;PANCREATIC-CANCER;SIALOMUCIN COMPLEX;TUMOR PROGRESSION;MUC4 EXPRESSION;POOR-PROGNOSIS;GENE ONTOLOGY;EXOSOMES;MICROENVIRONMENT;PATHOGENESIS |
| Hypoxia-Induced Adaptations of miRNomes and Proteomes in Melanoma Cells and Their Secreted Extracellular Vesicles | CANCERS; 2020 | Walbrecq, G and Lecha, O and Gaigneaux, A and Fougeras, MR and Philippidou, D and Margue, C and Nomigni, MT and Bernardin, F and Dittmar, G and Behrmann, I and Kreis, S and Walbrecq, Geoffroy and Lecha, Odile and Gaigneaux, Anthoula and Fougeras, Miriam R. and Philippidou, Demetra and Margue, Christiane and Nomigni, Milene Tetsi and Bernardin, Francois and Dittmar, Gunnar and Behrmann, Iris and Kreis, Stephanie | 10.3390/cancers12030692 | ARGININE METHYLTRANSFERASE;CANCER-CELLS;EXOSOMES;MELANOCYTES;METABOLISM;EXPRESSION;INVASION |
| Comparative Study of Metastasis Suppression Effects of Extracellular Vesicles Derived from Anaplastic Cell Lines, Nanog-Overexpressing Melanoma, and Induced Pluripotent Stem Cells | INTERNATIONAL JOURNAL OF MOLECULAR SCIENCES; 2023 | Khoo, CSM and Henmi, T and Saito, M and Khoo, Celine Swee May and Henmi, Takuya and Saito, Mikako | 10.3390/ijms242417206 | |
| Lactate increases tumor malignancy by promoting tumor small extracellular vesicles production <i>via</i> the GPR81-cAMP-PKA-HIF-1Î± axis | FRONTIERS IN ONCOLOGY; 2022 | Luo, M and Zhu, JQ and Ren, J and Tong, YX and Wang, LM and Ma, SL and Wang, JL and Luo, Man and Zhu, Junqi and Ren, Jie and Tong, Yuxiao and Wang, Limin and Ma, Shenglin and Wang, Jiaoli | 10.3389/fonc.2022.1036543 | CANCER;EXPRESSION;MIGRATION;EXOSOMES |
| Melanoma-derived small extracellular vesicles remodel the systemic onco-immunity via disrupting hematopoietic stem cell proliferation and differentiation | CANCER LETTERS; 2022 | Du, ZM and Feng, YY and Zhang, H and Liu, JB and Wang, JH and Du, Zhimin and Feng, Yueyuan and Zhang, Hui and Liu, Jinbao and Wang, Jinheng | 10.1016/j.canlet.2022.215841 EA AUG 2022 | BONE-MARROW MICROENVIRONMENT;CHECKPOINT BLOCKADE;PROGENITOR CELLS;MYELOID CELLS;T-CELLS;CANCER;EXOSOMES;MECHANISM;MICROVESICLES;RESISTANCE |
| A Rapid Exosome Isolation Using Ultrafiltration and Size Exclusion Chromatography (REIUS) Method for Exosome Isolation from Melanoma Cell Lines | MELANOMA: Methods and Protocols; 2021 | Shu, SL and Allen, CL and Benjamin-Davalos, S and Koroleva, M and MacFarland, D and Minderman, H and Ernstoff, MS and Shu, Shin La and Allen, Cheryl L. and Benjamin-Davalos, Shawna and Koroleva, Marina and MacFarland, Don and Minderman, Hans and Ernstoff, Marc S. | 10.1007/978-1-0716-1205-7_22 D2 10.1007/978-1-0716-1205-7 | TUMOR-DERIVED EXOSOMES;BIOLOGY |
| Tumor-derived microRNAs induce myeloid suppressor cells and predict immunotherapy resistance in melanoma | JOURNAL OF CLINICAL INVESTIGATION; 2018 | Huber, V and Vallacchi, V and Fleming, V and Hu, XY and Cova, A and Dugo, M and Shahaj, E and Sulsenti, R and Vergani, E and Filipazzi, P and De Laurentiis, A and Lalli, L and Di Guardo, L and Patuzzo, R and Vergani, B and Casiraghi, E and Cossa, M and Gualeni, A and Bollati, V and Arienti, F and De Braud, F and Mariani, L and Villa, A and Altevogt, P and Umansky, V and Rodolfo, M and Rivoltini, L and Huber, Veronica and Vallacchi, Viviana and Fleming, Viktor and Hu, Xiaoying and Cova, Agata and Dugo, Matteo and Shahaj, Eriomina and Sulsenti, Roberta and Vergani, Elisabetta and Filipazzi, Paola and De Laurentiis, Angela and Lalli, Luca and Di Guardo, Lorenza and Patuzzo, Roberto and Vergani, Barbara and Casiraghi, Elena and Cossa, Mara and Gualeni, Ambra and Bollati, Valentina and Arienti, Flavio and De Braud, Filippo and Mariani, Luigi and Villa, Antonello and Altevogt, Peter and Umansky, Viktor and Rodolfo, Monica and Rivoltini, Licia | 10.1172/JCI98060 | EXTRACELLULAR VESICLES;LIQUID BIOPSY;EXOSOMES;ACTIVATION;GENE;RESPONSES;PLASTICITY;PHENOTYPE;MONOCYTES;EXPANSION |
| Tumor-associated factors are enriched in lymphatic exudate compared to plasma in metastatic melanoma patients | JOURNAL OF EXPERIMENTAL MEDICINE; 2019 | Broggi, MAS and Maillat, L and Clement, CC and Bordry, N and CorthÃ©sy, P and Auger, A and Matter, M and Hamelin, R and Potin, L and Demurtas, D and Romano, E and Harari, A and Speiser, DE and Santambrogio, L and Swartz, MA and Broggi, Maria A. S. and Maillat, Lea and Clement, Cristina C. and Bordry, Natacha and Corthesy, Patricia and Auger, Aymeric and Matter, Maurice and Hamelin, Romain and Potin, Lambert and Demurtas, Davide and Romano, Emanuela and Harari, Alexandre and Speiser, Daniel E. and Santambrogio, Laura and Swartz, Melody A. | 10.1084/jem.20181618 | CANCER PROGRESSION;PROTEOMIC ANALYSIS;EXOSOMES;CELLS;PATHWAYS;MACROPHAGES;PEPTIDES;PROTEINS;INVASION |
| Therapeutic plasma exchange clears circulating soluble PD-L1 and PD-L1-positive extracellular vesicles | JOURNAL FOR IMMUNOTHERAPY OF CANCER; 2020 | Orme, JJ and Enninga, EAL and Lucien-Matteoni, F and Dale, H and Burgstaler, E and Harrington, SM and Ball, MK and Mansfield, AS and Park, SS and Block, MS and Markovic, SN and Yan, YY and Dong, HD and Dronca, RS and Winters, JL and Orme, Jacob J. and Enninga, Elizabeth Ann L. and Lucien-Matteoni, Fabrice and Dale, Heather and Burgstaler, Edwin and Harrington, Susan M. and Ball, Matthew K. and Mansfield, Aaron S. and Park, Sean S. and Block, Mathew S. and Markovic, Svetomir N. and Yan, Yiyi and Dong, Haidong and Dronca, Roxana S. and Winters, Jeffrey L. | 10.1136/jitc-2020-001113 | RANDOMIZED PHASE-2;BEVACIZUMAB;CARBOPLATIN;PACLITAXEL;MELANOMA |
| N-glycome inheritance from cells to extracellular vesicles in B16 melanomas | FEBS LETTERS; 2019 | Harada, Y and Kizuka, Y and Tokoro, Y and Kondo, K and Yagi, H and Kato, K and Inoue, H and Taniguchi, N and Maruyama, I and Harada, Yoichiro and Kizuka, Yasuhiko and Tokoro, Yuko and Kondo, Kiyotaka and Yagi, Hirokazu and Kato, Koichi and Inoue, Hiromasa and Taniguchi, Naoyuki and Maruyama, Ikuro | 10.1002/1873-3468.13377 | CDNA CLONING;METASTASIS;GLYCOSYLATION;GLYCOPROTEINS;PURIFICATION;SUPPRESSION;PROGRESSION;INTEGRINS;SURVIVAL;ARREST |
| Generation of the heterogeneity of extracellular vesicles by membrane organization and sorting machineries | BIOCHIMICA ET BIOPHYSICA ACTA-GENERAL SUBJECTS; 2019 | Harada, Y and Suzuki, T and Fukushige, T and Kizuka, Y and Yagi, H and Yamamoto, M and Kondo, K and Inoue, H and Kato, K and Taniguchi, N and Kanekura, T and Dohmae, N and Maruyama, I and Harada, Yoichiro and Suzuki, Takehiro and Fukushige, Tomoko and Kizuka, Yasuhiko and Yagi, Hirokazu and Yamamoto, Mika and Kondo, Kiyotaka and Inoue, Hiromasa and Kato, Koichi and Taniguchi, Naoyuki and Kanekura, Takuro and Dohmae, Naoshi and Maruyama, Ikuro | 10.1016/j.bbagen.2019.01.015 | DETERGENT-RESISTANT MEMBRANES;PROTEINS;EXOSOMES;CELLS;UBIQUITIN;ADAM10;MET;GLYCOSYLATION;MICROVESICLES;LOCALIZATION |
| Small extracellular vesicle-based human melanocyte and melanoma signature | PIGMENT CELL & MELANOMA RESEARCH; 2023 | AgÃ¼era-Lorente, A and Alonso-Pardavila, A and Larrinaga, M and Boyano, MD and GonzÃ¡lez, E and FalcÃ³n-PÃ©rez, JM and Asumendi, A and Apraiz, A and Aguera-Lorente, Andrea and Alonso-Pardavila, Ainhoa and Larrinaga, Maria and Boyano, Maria Dolores and Gonzalez, Esperanza and Falcon-Perez, Juan Manuel and Asumendi, Aintzane and Apraiz, Aintzane | 10.1111/pcmr.13158 EA DEC 2023 | TRANSFERRIN RECEPTOR;CELL |
| Anti-human CD9 antibody Fab fragment impairs the internalization of extracellular vesicles and the nuclear transfer of their cargo proteins | JOURNAL OF CELLULAR AND MOLECULAR MEDICINE; 2019 | Santos, MF and Rappa, G and KarbanovÃ¡, J and Vanier, C and Morimoto, C and Corbeil, D and Lorico, A and Santos, Mark F. and Rappa, Germana and Karbanova, Jana and Vanier, Cheryl and Morimoto, Chikao and Corbeil, Denis and Lorico, Aurelio | 10.1111/jcmm.14334 | OF-THE-ART;TETRASPANIN CD9;MEMBRANE-PARTICLES;INTEGRIN ALPHA-6-BETA-1;MEDIATED ENDOCYTOSIS;NEURAL PROGENITORS;EXOSOME UPTAKE;STROMAL CELLS;GENOMIC DNA;FUSION |
| Extracellular vesicles promote migration despite BRAF inhibitor treatment in malignant melanoma cells | CELL COMMUNICATION AND SIGNALING; 2024 | NÃ©meth, A and BÃ¡nyai, GL and Dobos, NK and KÃ³s, T and GaÃ¡l, A and Varga, Z and BuzÃ¡s, EI and Khamari, D and Dank, M and TakÃ¡cs, I and SzÃ¡sz, AM and Garay, T and Nemeth, Afrodite and Banyai, Greta L. and Dobos, Nikolett K. and Kos, Tamas and Gaal, Aniko and Varga, Zoltan and Buzas, Edit I. and Khamari, Delaram and Dank, Magdolna and Takacs, Istvan and Szasz, A. Marcell and Garay, Tamas | 10.1186/s12964-024-01660-4 | METASTATIC PHENOTYPE;ACQUIRED-RESISTANCE;VEMURAFENIB;EXOSOMES;BRAF(V600E);SURVIVAL;GROWTH |
| Microenvironmental pH and Exosome Levels Interplay in Human Cancer Cell Lines of Different Histotypes | CANCERS; 2018 | Logozzi, M and Mizzoni, D and Angelini, DF and Di Raimo, R and Falchi, M and Battistini, L and Fais, S and Logozzi, Mariantonia and Mizzoni, Davide and Angelini, Daniela F. and Di Raimo, Rossella and Falchi, Mario and Battistini, Luca and Fais, Stefano | 10.3390/cancers10100370 | PROTON PUMP INHIBITORS;HUMAN-MELANOMA;MICROVESICLES;MICRORNAS;VESICLES;DELIVERY;INDUCE |
| miR-1246 in tumor extracellular vesicles promotes metastasis <i>via</i> increased tumor cell adhesion and endothelial cell barrier destruction | FRONTIERS IN ONCOLOGY; 2023 | Morimoto, M and Maishi, N and Tsumita, T and Alam, MT and Kikuchi, H and Hida, Y and Yoshioka, Y and Ochiya, T and Annan, DA and Takeda, R and Kitagawa, Y and Hida, K and Morimoto, Masahiro and Maishi, Nako and Tsumita, Takuya and Alam, Mohammad Towfik and Kikuchi, Hiroshi and Hida, Yasuhiro and Yoshioka, Yusuke and Ochiya, Takahiro and Annan, Dorcas A. and Takeda, Ryo and Kitagawa, Yoshimasa and Hida, Kyoko | 10.3389/fonc.2023.973871 | COLORECTAL-CANCER;ANGIOGENESIS;MICRORNAS;JUNCTIONS |
| Double Digital Assay for Single Extracellular Vesicle and Single Molecule Detection | ADVANCED SCIENCE; 2023 | Reynolds, DE and Pan, MH and Yang, JB and Galanis, G and Roh, YH and Morales, RTT and Kumar, SS and Heo, SJ and Xu, XW and Guo, W and Ko, JA and Reynolds, David E. and Pan, Menghan and Yang, Jingbo and Galanis, George and Roh, Yoon Ho and Morales, Renee-Tyler T. and Kumar, Shailesh Senthil and Heo, Su-Jin and Xu, Xiaowei and Guo, Wei and Ko, Jina | 10.1002/advs.202303619 EA OCT 2023 | EXOSOMES;EXPRESSION;CANCER;PLASMA;PD-L1 |
| RNA in Salivary Extracellular Vesicles as a Possible Tool for Systemic Disease Diagnosis | JOURNAL OF DENTAL RESEARCH; 2017 | Kim, J and Shin, H and Park, J and Kim, J. and Shin, H. and Park, J. | 10.1177/0022034517702100 | TUMOR-MARKER;BIOMARKER;EXOSOMES;MELANOMA;MODEL |
| Melanoma-Derived Exosomes Induce PD-1 Overexpression and Tumor Progression via Mesenchymal Stem Cell Oncogenic Reprogramming | FRONTIERS IN IMMUNOLOGY; 2019 | Gyukity-SebestyÃ©n, E and Harmati, M and Dobra, G and NÃ©meth, IB and MihÃ¡ly, J and Zvara, A and Hunyadi-GulyÃ¡s, Ã‰ and Katona, R and Nagy, I and HorvÃ¡th, P and BÃ¡lind, A and Szkalisity, A and KovÃ¡cs, M and Pankotai, T and Borsos, B and ErdÃ©lyi, M and Szegletes, Z and VerÃ©b, ZJ and Buzas, EI and KemÃ©ny, L and BirÃ³, T and BuzÃ¡s, K and Gyukity-Sebestyen, Edina and Harmati, Maria and Dobra, Gabriella and Nemeth, Istvan B. and Mihaly, Johanna and Zvara, Agnes and Hunyadi-Gulyas, Eva and Katona, Robert and Nagy, Istvan and Horvath, Peter and Balind, Arpad and Szkalisity, Abel and Kovacs, Maria and Pankotai, Tibor and Borsos, Barbara and Erdelyi, Miklos and Szegletes, Zsolt and Vereb, Zoltan J. and Buzas, Edit, I and Kemeny, Lajos and Biro, Tamas and Buzas, Krisztina | 10.3389/fimmu.2019.02459 | EXTRACELLULAR VESICLES;METASTASIS;GROWTH;COMMUNICATION;INHIBITION;EXPRESSION;INVASION;MARKER;TARGET |
| Plasma-derived extracellular vesicle analysis and deconvolution enable prediction and tracking of melanoma checkpoint blockade outcome | SCIENCE ADVANCES; 2020 | Shi, A and Kasumova, GG and Michaud, WA and Cintolo-Gonzalez, J and DÃ­az-MartÃ­nez, M and Ohmura, J and Mehta, A and Chien, I and Frederick, DT and Cohen, S and Plana, D and Johnson, D and Flaherty, KT and Sullivan, RJ and Kellis, M and Boland, GM and Shi, Alvin and Kasumova, Gyulnara G. and Michaud, William A. and Cintolo-Gonzalez, Jessica and Diaz-Martinez, Marta and Ohmura, Jacqueline and Mehta, Arnav and Chien, Isabel and Frederick, Dennie T. and Cohen, Sonia and Plana, Deborah and Johnson, Douglas and Flaherty, Keith T. and Sullivan, Ryan J. and Kellis, Manolis and Boland, Genevieve M. | 10.1126/sciadv.abb3461 | MOLECULAR SIGNATURES;EXPRESSION;EXOSOMES;IMMUNOTHERAPY;CELLS |
| Isolation of High-Purity Extracellular Vesicles by Extracting Proteins Using Aqueous Two-Phase System | PLOS ONE; 2015 | Kim, J and Shin, H and Kim, J and Kim, J and Park, J and Kim, Jongmin and Shin, Hyunwoo and Kim, Jiyoon and Kim, Junho and Park, Jaesung | 10.1371/journal.pone.0129760 | MEMBRANE-VESICLES;PLASMA-MEMBRANES;MESSENGER-RNA;INSIDE-OUT;EXOSOMES;SEPARATION;CELL |
| Use of extracellular vesicles from lymphatic drainage as surrogate markers of melanoma progression and BRAF<i><SUP>V600E</SUP></i> mutation | JOURNAL OF EXPERIMENTAL MEDICINE; 2019 | GarcÃ­a-Silva, S and Benito-MartÃ­n, A and SÃ¡nchez-Redondo, S and HernÃ¡ndez-Barranco, A and XimÃ©nez-EmbÃºn, P and NoguÃ©s, L and Mazariegos, MS and Brinkmann, K and LÃ³pez, AA and Meyer, L and RodrÃ­guez, C and GarcÃ­a-MartÃ­n, C and Boskovic, J and LetÃ³n, R and Montero, C and Robledo, M and Santambrogio, L and Brady, MS and Szumera-Cieckiewicz, A and Kalinowska, I and Skog, J and Noerholm, M and MuÃ±oz, J and Ortiz-Romero, PL and Ruano, Y and RodrÃ­guez-Peralto, JL and Rutkowski, P and Peinado, H and Garcia-Silva, Susana and Benito-Martin, Alberto and Sanchez-Redondo, Sara and Hernandez-Barranco, Alberto and Ximenez-Embun, Pilar and Nogues, Laura and Mazariegos, Marina S. and Brinkmann, Kay and Amor Lopez, Ana and Meyer, Lisa and Rodriguez, Carlos and Garcia-Martin, Carmen and Boskovic, Jasminka and Leton, Rocio and Montero, Cristina and Robledo, Mercedes and Santambrogio, Laura and Brady, Mary Sue and Szumera-Cieckiewicz, Anna and Kalinowska, Iwona and Skog, Johan and Noerholm, Mikkel and Munoz, Javier and Ortiz-Romero, Pablo L. and Ruano, Yolanda and Rodriguez-Peralto, Jose L. and Rutkowski, Piotr and Peinado, Hector | 10.1084/jem.20181522 | MUTANT KRAS;EXOSOMES;DNA;CELLS |
| Evidence for liver and peripheral immune cells secreting tumor-suppressive extracellular vesicles in melanoma patients | EBIOMEDICINE; 2020 | Lee, JH and Eberhardt, M and Blume, K and Vera, J and Baur, AS and Lee, Jung-Hyun and Eberhardt, Martin and Blume, Katja and Vera, Julio and Baur, Andreas S. | 10.1016/j.ebiom.2020.103119 | HIV-NEF;RESISTANCE;INHIBITORS;EXOSOMES;FATE |
| Tumor-Derived Membrane Vesicles from the IL-2 Overexpression Melanoma Cells Affect on the Expression of Surface Markers of Human Peripheral Blood Mononuclear Cells In Vitro | BIONANOSCIENCE; 2023 | Filin, IY and Kitaeva, KV and Chulpanova, DS and Rizvanov, AA and Akhmetzyanova, ER and Solovyeva, VV and Filin, Ivan Yu and Kitaeva, Kristina, V and Chulpanova, Daria S. and Rizvanov, Albert A. and Akhmetzyanova, Elvira R. and Solovyeva, Valeriya V. | 10.1007/s12668-022-01044-3 EA NOV 2022 | EXTRACELLULAR VESICLES;RESPONSES |
| Immunosuppressive functions of melanoma cell-derived exosomes in plasma of melanoma patients | FRONTIERS IN CELL AND DEVELOPMENTAL BIOLOGY; 2023 | Whiteside, TL and Whiteside, Theresa L. | 10.3389/fcell.2022.1080925 | CANCER |
| Extracellular vesicles from plasma have higher tumour RNA fraction than platelets | JOURNAL OF EXTRACELLULAR VESICLES; 2020 | Brinkman, K and Meyer, L and Bickel, A and Enderle, D and Berking, C and Skog, J and Noerholm, M and Brinkman, Kay and Meyer, Lisa and Bickel, Anne and Enderle, Daniel and Berking, Carola and Skog, Johan and Noerholm, Mikkel | 10.1080/20013078.2020.1741176 | EDUCATED PLATELETS;BLOOD-PLATELETS;LIQUID BIOPSY;CANCER |
| Characterization of plasma circulating small extracellular vesicles in patients with metastatic solid tumors and newly diagnosed brain metastasis | ONCOIMMUNOLOGY; 2022 | Carretero-GonzÃ¡lez, A and Hergueta-Redondo, M and SÃ¡nchez-Redondo, S and XimÃ©nez-EmbÃºn, P and SÃ¡nchez, LM and Gil, EC and Castellano, D and de Velasco, G and Peinado, H and Carretero-Gonzalez, Alberto and Hergueta-Redondo, Marta and Sanchez-Redondo, Sara and Ximenez-Embun, Pilar and Manso Sanchez, Luis and Ciruelos Gil, Eva and Castellano, Daniel and de Velasco, Guillermo and Peinado, Hector | 10.1080/2162402X.2022.2067944 | CANCER;STAT3;CELLS;EXOSOMES;SUPPRESSION;MELANOMA;EPIDEMIOLOGY;ACTIVATION;CROSSTALK;NICHE |
| Coronin 1C inhibits melanoma metastasis through regulation of MT1-MMP-containing extracellular vesicle secretion | SCIENTIFIC REPORTS; 2020 | Tagliatela, AC and Hempstead, SC and Hibshman, PS and Hockenberry, MA and Brighton, HE and Pecot, C and Bear, JE and Tagliatela, Alicia C. and Hempstead, Stephanie C. and Hibshman, Priya S. and Hockenberry, Max A. and Brighton, Hailey E. and Pecot, Chad, V and Bear, James E. | 10.1038/s41598-020-67465-w | MATRIX-METALLOPROTEINASE MT1-MMP;CELL-MIGRATION;1-MATRIX METALLOPROTEINASE;EXOSOME SECRETION;DOWN-REGULATION;MEMBRANE;INVASION;PROTEIN;INVADOPODIA;MELANOSOMES |
| Identification of novel, immune-mediating extracellular vesicles in human lymphatic effluent draining primary cutaneous melanoma | ONCOIMMUNOLOGY; 2019 | Maus, RLG and Jakub, JW and Hieken, TJ and Nevala, WK and Christensen, TA and Sutor, SL and Flotte, TJ and Markovic, SN and Maus, Rachel L. G. and Jakub, James W. and Hieken, Tina J. and Nevala, Wendy K. and Christensen, Trace A. and Sutor, Shari L. and Flotte, Thomas J. and Markovic, Svetomir N. | 10.1080/2162402X.2019.1667742 EA SEP 2019 | SENTINEL-NODE BIOPSY;DENDRITIC CELLS;BREAST-CANCER;TUMOR;METASTASIS;MELANOCYTES;KERATINOCYTES;LYMPHANGIOGENESIS;EXPRESSION;EXOSOMES |
| Isolation of extracellular vesicles improves the detection of mutant DNA from plasma of metastatic melanoma patients | SCIENTIFIC REPORTS; 2020 | Zocco, D and Bernardi, S and Novelli, M and Astrua, C and Fava, P and Zarovni, N and Carpi, FM and Bianciardi, L and Malavenda, O and Quaglino, P and Foroni, C and Russo, D and Chiesi, A and Fierro, MT and Zocco, Davide and Bernardi, Simona and Novelli, Mauro and Astrua, Chiara and Fava, Paolo and Zarovni, Natasa and Carpi, Francesco M. and Bianciardi, Laura and Malavenda, Ottavia and Quaglino, Pietro and Foroni, Chiara and Russo, Domenico and Chiesi, Antonio and Fierro, Maria Teresa | 10.1038/s41598-020-72834-6 | MUTATION ANALYSIS;SERUM EXOSOMES;BRAF MUTATIONS;DIGITAL PCR;CANCER;KRAS;MICROVESICLES;INFORMATION;GUIDELINES;SURVIVAL |
| In vivo visualization of murine melanoma cells B16-derived exosomes through magnetic resonance imaging | BIOCHIMICA ET BIOPHYSICA ACTA-GENERAL SUBJECTS; 2022 | Liu, TQ and Li, ZL and Li, XD and Zhao, RT and Wei, XH and Wang, ZX and Xin, SXG and Liu, Tianqi and Li, Zhenlin and Li, Xiaodong and Zhao, Ruiting and Wei, Xinhua and Wang, Zixin and Xin, Sherman Xuegang | 10.1016/j.bbagen.2021.130062 EA NOV 2021 | EXTRACELLULAR VESICLES;REPORTER GENE;STEM-CELLS;FERRITIN;MRI;BIODISTRIBUTION;EXPRESSION;TRACKING;DIFFERENTIATION;LACTADHERIN |
| uPAR<SUP>+</SUP> extracellular vesicles: a robust biomarker of resistance to checkpoint inhibitor immunotherapy in metastatic melanoma patients | JOURNAL FOR IMMUNOTHERAPY OF CANCER; 2021 | Porcelli, L and Guida, M and De Summa, S and Di Fonte, R and De Risi, I and Garofoli, M and Caputo, M and Negri, A and Strippoli, S and SerratÃ¬, S and Azzariti, A and Porcelli, Letizia and Guida, Michele and De Summa, Simona and Di Fonte, Roberta and De Risi, Ivana and Garofoli, Marianna and Caputo, Mariapia and Negri, Antonio and Strippoli, Sabino and Serrati, Simona and Azzariti, Amalia | 10.1136/jitc-2021-002372 | EXOSOMES;PHENOTYPE;IMMUNITY;THERAPY;MARKERS;SYSTEM;S100 |
| Melanoma extracellular vesicles inhibit tumor growth and metastasis by stimulating CD8 T cells | MOLECULAR IMMUNOLOGY; 2024 | Dan, YX and Ma, J and Long, YQ and Jiang, Y and Fang, LQ and Bai, J and Dan, Yuxi and Ma, Jing and Long, Yuqing and Jiang, Yao and Fang, Liaoqiong and Bai, Jin | 10.1016/j.molimm.2024.03.003 EA MAR 2024 | DENDRITIC CELLS;VACCINE |
| Role of Extracellular Vesicle Surface Proteins in the Pharmacokinetics of Extracellular Vesicles | MOLECULAR PHARMACEUTICS; 2018 | Charoenyiriyakul, C and Takahashi, Y and Morishita, M and Nishikawa, M and Takakura, Y and Charoenyiriyakul, Chonlada and Takahashi, Yuki and Morishita, Masaki and Nishikawa, Makiya and Takakura, Yoshinobu | 10.1021/acs.molpharmaceut.7b00950 | PHYSICOCHEMICAL PROPERTIES;B16BL6-DERIVED EXOSOMES;BLOOD-CIRCULATION;DELIVERY VEHICLES;DRUG-DELIVERY;CELLS;NANOPARTICLES;CLEARANCE;INJECTION;MELANOMA |
| Comprehensive Analyses of miRNAs Revealed miR-92b-3p, miR-182-5p and miR-183-5p as Potential Novel Biomarkers in Melanoma-Derived Extracellular Vesicles | FRONTIERS IN ONCOLOGY; 2022 | Gerloff, D and Kewitz-Hempel, S and Hause, G and Ehrenreich, J and Golle, L and Kingreen, T and Sunderkoetter, C and Gerloff, Dennis and Kewitz-Hempel, Stefanie and Hause, Gerd and Ehrenreich, Jovine and Golle, Linda and Kingreen, Tim and Sunderkoetter, Cord | 10.3389/fonc.2022.935816 | ENDOTHELIAL-CELLS;EXOSOMES;MICRORNA;CANCER;RNA;ANGIOGENESIS;MECHANISM;MIGRATION |
| Differential ultracentrifugation enables deep plasma proteomics through enrichment of extracellular vesicles | PROTEOMICS; 2023 | Kverneland, AH and Ostergaard, O and Emdal, KB and Svane, IM and Olsen, JV and Kverneland, Anders H. and Ostergaard, Ole and Emdal, Kristina Bennet and Svane, Inge Marie and Olsen, Jesper Velgaard | 10.1002/pmic.202200039 EA NOV 2022 | MASS-SPECTROMETRY;INFLAMMATION;BIOMARKER |
| Isolation and characterization of extracellular vesicle subpopulations from tissues | NATURE PROTOCOLS; 2021 | Crescitelli, R and Lasser, C and Lattvall, J and Crescitelli, Rossella and Lasser, Cecilia and Lotvall, Jan | 10.1038/s41596-020-00466-1 EA JAN 2021 | IMMUNE-RESPONSE;MELANOMA-CELLS;IN-VIVO;EXOSOMES;EXPRESSION;MICROVESICLES;SECRETOME;DELIVERY |
| CA-IX-Expressing Small Extracellular Vesicles (sEVs) Are Released by Melanoma Cells under Hypoxia and in the Blood of Advanced Melanoma Patients | INTERNATIONAL JOURNAL OF MOLECULAR SCIENCES; 2023 | Venturella, M and Falsini, A and Coppola, F and Giuntini, G and Carraro, F and Zocco, D and Chiesi, A and Naldini, A and Venturella, Marta and Falsini, Alessandro and Coppola, Federica and Giuntini, Gaia and Carraro, Fabio and Zocco, Davide and Chiesi, Antonio and Naldini, Antonella | 10.3390/ijms24076122 | CARBONIC-ANHYDRASE-IX;CANCER-CELLS;EXOSOMES;OVEREXPRESSION;INHIBITORS;BIOLOGY;SERUM;XII |
| Immunoaffinity-based isolation of melanoma cell-derived exosomes from plasma of patients with melanoma | JOURNAL OF EXTRACELLULAR VESICLES; 2018 | Sharma, P and Ludwig, S and Muller, L and Hong, CS and Kirkwood, JM and Ferrone, S and Whiteside, TL and Sharma, Priyanka and Ludwig, Sonja and Muller, Laurent and Hong, Chang Sook and Kirkwood, John M. and Ferrone, Soldano and Whiteside, Theresa L. | 10.1080/20013078.2018.1435138 | T-CELL;EXTRACELLULAR VESICLES;ANTIGEN;CANCER;MICROVESICLES;EXPRESSION;ANTIBODY;CSPG4 |
| The Fatty Acid and Protein Profiles of Circulating CD81-Positive Small Extracellular Vesicles Are Associated with Disease Stage in Melanoma Patients | CANCERS; 2021 | Paolino, G and Huber, V and Camerini, S and Casella, M and Macone, A and Bertuccini, L and Iosi, F and Moliterni, E and Cecchetti, S and Ruspantini, I and Chiarotti, F and Vergani, E and Lalli, L and Raggi, C and Di Biase, A and Calvieri, S and Mercuri, S and Lugini, L and Federici, C and Paolino, Giovanni and Huber, Veronica and Camerini, Serena and Casella, Marialuisa and Macone, Alberto and Bertuccini, Lucia and Iosi, Francesca and Moliterni, Elisa and Cecchetti, Serena and Ruspantini, Irene and Chiarotti, Flavia and Vergani, Elisabetta and Lalli, Luca and Raggi, Carla and Di Biase, Antonella and Calvieri, Stefano and Mercuri, Santo Raffaele and Lugini, Luana and Federici, Cristina | 10.3390/cancers13164157 | OLEIC-ACID;INTERACTION NETWORK;BREAST-CANCER;TUMOR-CELLS;EXOSOMES;PROGRESSION;METABOLISM;MARKER;RATIO;BIOMARKERS |
| Defining the Soluble and Extracellular Vesicle Protein Compartments of Plasma Using In-Depth Mass Spectrometry-Based Proteomics | JOURNAL OF PROTEOME RESEARCH; 2024 | Sharma, N and Angori, S and Sandberg, A and Mermelekas, G and LehtiÃ¶, J and Wiklander, OPB and GÃ¶rgens, A and El Andaloussi, S and Eriksson, H and Pernemalm, M and Sharma, Nidhi and Angori, Silvia and Sandberg, AnnSofi and Mermelekas, Georgios and Lehtio, Janne and Wiklander, Oscar P. B. and Gorgens, Andre and El Andaloussi, Samir and Eriksson, Hanna and Pernemalm, Maria | 10.1021/acs.jproteome.4c00490 EA AUG 2024 | |
| Isolation of circulating exosomes and identification of exosomal PD-L1 for predicting immunotherapy response | NANOSCALE; 2022 | Zhang, JL and Zhu, YF and Guan, MT and Liu, YY and Lv, M and Zhang, CW and Zhang, HL and Zhang, ZZ and Zhang, Junli and Zhu, Yifan and Guan, Mengting and Liu, Yingying and Lv, Min and Zhang, Chongwei and Zhang, Hongling and Zhang, Zhenzhong | 10.1039/d2nr00829g EA MAY 2022 | EXTRACELLULAR VESICLES |
| Serum exosomes as predictors of clinical response to ipilimumab in metastatic melanoma | ONCOIMMUNOLOGY; 2018 | Tucci, M and Passarelli, A and Mannavola, F and Stucci, LS and Ascierto, PA and Capone, M and Madonna, G and Lopalco, P and Silvestris, F and Tucci, Marco and Passarelli, Anna and Mannavola, Francesco and Stucci, Luigia Stefania and Ascierto, Paolo Antonio and Capone, Marilena and Madonna, Gabriele and Lopalco, Patrizia and Silvestris, Francesco | 10.1080/2162402X.2017.1387706 | CELL-DERIVED EXOSOMES;EXTRACELLULAR VESICLES;DENDRITIC CELLS;T-CELLS;BIOMARKERS;NIVOLUMAB;EVEROLIMUS;CORRELATE;EFFECTOR;SURVIVAL |
| The Proangiogenic Effects of Melanoma-Derived Ectosomes Are Mediated by Î±vÎ²5 Integrin Rather than Î±vÎ²3 Integrin | CELLS; 2024 | Surman, M and Wilczak, M and Bzowska, M and Tylko, G and Przybylo, M and Surman, Magdalena and Wilczak, Magdalena and Bzowska, Malgorzata and Tylko, Grzegorz and Przybylo, Malgorzata | 10.3390/cells13161336 | VERTICAL GROWTH-PHASE;PROMOTE ANGIOGENESIS;CELLS;MICROVESICLES;CILENGITIDE;EXPRESSION;GLIOBLASTOMA;PROGRESSION;INHIBITION;VESICLES |
| Exosome Isolation: Cyclical Electrical Field Flow Fractionation in Low-Ionic-Strength Fluids | ANALYTICAL CHEMISTRY; 2018 | Petersen, KE and Shiri, F and White, T and Bardi, GT and Sant, H and Gale, BK and Hood, JL and Petersen, Kevin E. and Shiri, Farhad and White, Travis and Bardi, Gina T. and Sant, Himanshu and Gale, Bruce K. and Hood, Joshua L. | 10.1021/acs.analchem.8b03146 | EXTRACELLULAR VESICLES;MELANOMA EXOSOMES;STEM-CELLS;SEPARATION;PROTEINS;EXOCARTA;MOBILITY;LIGHT;SIZE;RNA |
| Gold nanoparticle based double-labeling of melanoma extracellular vesicles to determine the specificity of uptake by cells and preferential accumulation in small metastatic lung tumors | JOURNAL OF NANOBIOTECHNOLOGY;2020 | Lara, P and Palma-Florez, S and Salas-Huenuleo, E and Polakovicova, I and Guerrero, S and Lobos-Gonzalez, L and Campos, A and MuÃ±oz, L and Jorquera-Cordero, C and Varas-Godoy, M and Cancino, J and Arias, E and Villegas, J and Cruz, LJ and Albericio, F and Araya, E and Corvalan, AH and Quest, AFG and Kogan, MJ and Lara, Pablo and Palma-Florez, Sujey and Salas-Huenuleo, Edison and Polakovicova, Iva and Guerrero, Simon and Lobos-Gonzalez, Lorena and Campos, America and Munoz, Luis and Jorquera-Cordero, Carla and Varas-Godoy, Manuel and Cancino, Jorge and Arias, Eloisa and Villegas, Jaime and Cruz, Luis J. and Albericio, Fernando and Araya, Eyleen and Corvalan, Alejandro H. and Quest, Andrew F. G. and Kogan, Marcelo J. | 10.1186/s12951-020-0573-0 | CONJUGATING FOLIC-ACID;EXOSOMES;DELIVERY;BIODISTRIBUTION;TOXICITY;SYSTEM;SIZE;THERAPY |
| Innate extracellular vesicles from melanoma patients suppress Î²-catenin in tumor cells by miRNA-34a | LIFE SCIENCE ALLIANCE; 2019 | Lee, JH and Dindorf, J and Eberhardt, M and Lai, X and Ostalecki, C and Koliha, N and Gross, S and Blume, K and Bruns, H and Wild, S and Schuler, G and Vera, J and Baur, AS and Lee, Jung-Hyun and Dindorf, Jochen and Eberhardt, Martin and Lai, Xin and Ostalecki, Christian and Koliha, Nina and Gross, Stefani and Blume, Katja and Bruns, Heiko and Wild, Stefan and Schuler, Gerold and Vera, Julio and Baur, Andreas S. | 10.26508/lsa.201800205 | HIV-NEF;TRANSCRIPTION FACTORS;CANCER EXOSOMES;METASTASIS;SECRETION;MICROPARTICLES;MICROVESICLES;ACTIVATION;CLEARANCE;TARGETS |
| Mitochondrial protein enriched extracellular vesicles discovered in human melanoma tissues can be detected in patient plasma | JOURNAL OF EXTRACELLULAR VESICLES; 2019 | Jang, SC and Crescitelli, R and Cvjetkovic, A and Belgrano, V and Bagge, RO and Sundfeldt, K and Ochiya, T and Kalluri, R and LÃ¶tvall, J and Jang, Su Chul and Crescitelli, Rossella and Cvjetkovic, Aleksander and Belgrano, Valerio and Bagge, Roger Olofsson and Sundfeldt, Karin and Ochiya, Takahiro and Kalluri, Raghu and Lotvall, Jan | 10.1080/20013078.2019.1635420 | CANCER-CELLS;EXOSOMES;MEMBRANE;IDENTIFICATION;BIOGENESIS;PROTEOMICS;MARKERS |
| Ultrasensitive quantification of PD-L1+extracellular vesicles in melanoma patient plasma using a parallelized high throughput droplet digital assay | LAB ON A CHIP; 2024 | Shen, HF and Atiyas, Y and Yang, ZJ and Lin, AA and Yang, JB and Liu, D and Park, J and Guo, W and Issadore, DA and Shen, Hanfei and Atiyas, Yasemin and Yang, Zijian and Lin, Andrew A. and Yang, Jingbo and Liu, Diao and Park, Juhwan and Guo, Wei and Issadore, David A. | 10.1039/d4lc00331d EA JUN 2024 | EXTRACELLULAR VESICLES;PROTEIN-DETECTION |
| Melanoma-derived extracellular vesicles skew neutrophils into a pro-tumor phenotype | JOURNAL OF LEUKOCYTE BIOLOGY; 2022 | Guimaraes-Bastos, D and Frony, AC and Barja-Fidalgo, C and Moraes, JA and Guimaraes-Bastos, Daniel and Frony, Ana Clara and Barja-Fidalgo, Christina and Moraes, Joao Alfredo | 10.1002/JLB.3A0120-050RR EA MAY 2021 | INTRATUMORAL NEUTROPHILS;CANCER;TRAPS;CELLS;INFLAMMATION;CARCINOMA;APOPTOSIS;EXOSOMES;INNATE;GROWTH |
| Investigating the tumor-immune microenvironment through extracellular vesicles from frozen patient biopsies and 3D cultures | FRONTIERS IN IMMUNOLOGY; 2023 | Al Hrout, A and Levesque, MP and Chahwan, R and Al Hrout, Ala'a and Levesque, Mitchell P. P. and Chahwan, Richard | 10.3389/fimmu.2023.1176175 | CELL-CULTURE;MECHANISMS;BIOMARKERS;RESISTANCE;MICRORNAS;MELANOMA;MODELS |
| Extracellular vesicle DNA from human melanoma tissues contains cancer-specific mutations | FRONTIERS IN CELL AND DEVELOPMENTAL BIOLOGY; 2022 | Crescitelli, R and Filges, S and Karimi, N and UrzÃ¬, O and Alonso-Agudo, T and StÃ¥hlberg, A and LÃ¶tvall, J and LÃ¤sser, C and Bagge, RO and Crescitelli, Rossella and Filges, Stefan and Karimi, Nasibeh and Urzi, Ornella and Alonso-Agudo, Tamara and Stahlberg, Anders and Lotvall, Jan and Lasser, Cecilia and Bagge, Roger Olofsson | 10.3389/fcell.2022.1028854 | CIRCULATING TUMOR DNA;CELL-FREE DNA;GENOMIC DNA;PLASMA;FRAGMENTS;DIAGNOSIS;EXOSOMES;MARKERS;BRAF |
| Melanoma-Derived Extracellular Vesicles Bear the Potential for the Induction of Antigen-Specific Tolerance | CELLS; 2019 | DÃ¼chler, M and Czernek, L and Peczek, L and Cypryk, W and Sztiller-Sikorska, M and Czyz, M and Duchler, Markus and Czernek, Liliana and Peczek, Lukasz and Cypryk, Wojciech and Sztiller-Sikorska, Malgorzata and Czyz, Malgorzata | 10.3390/cells8070665 | REGULATORY T-CELLS;CLASS-II+ EXOSOMES;DENDRITIC CELLS;TGF-BETA;SUPPRESSIVE FUNCTION;GROWTH-FACTOR;IMMUNITY;EXPRESSION;MICRORNAS;RECEPTOR |
| Rapid communication: insights into the role of extracellular vesicles during Auger radioimmunotherapy | INTERNATIONAL JOURNAL OF RADIATION BIOLOGY; 2023 | Karam, J and Constanzo, J and Pichard, A and Gros, L and Chopineau, J and Morille, M and Pouget, JP and Karam, Jihad and Constanzo, Julie and Pichard, Alexandre and Gros, Laurent and Chopineau, Joel and Morille, Marie and Pouget, Jean-Pierre | 10.1080/09553002.2021.1955999 EA JUL 2021 | IONIZING-RADIATION;CELL-MEMBRANE;EXOSOMES;RADIONUCLIDES;SECRETION;DOSIMETRY;CANCER;TUMOR;DNA |
| Assessment of ATP metabolism to adenosine by ecto-nucleotidases carried by tumor-derived small extracellular vesicles | PURINERGIC SIGNALLING; 2024 | Hong, CS and Menshikova, EV and Whiteside, TL and Jackson, EK and Hong, Chang-Sook and Menshikova, Elizabeth V. and Whiteside, Theresa L. and Jackson, Edwin K. | 10.1007/s11302-024-10038-7 EA JUL 2024 | T-CELLS;EXOSOMES;CANCER;RELEASE;CD73;MECHANISM;POTENT |
| HAS3-induced extracellular vesicles from melanoma cells stimulate IHH mediated c-Myc upregulation via the hedgehog signaling pathway in target cells | CELLULAR AND MOLECULAR LIFE SCIENCES; 2020 | Arasu, UT and Deen, AJ and Pasonen-SeppÃ¤nen, S and Heikkinen, S and Lalowski, M and KÃ¤rnÃ¤, R and HÃ¤rkÃ¶nen, K and MÃ¤kinen, P and LÃ¡zaro-IbÃ¡Ã±ez, E and Siljander, PRM and Oikari, S and Levonen, AL and Rilla, K and Arasu, Uma Thanigai and Deen, Ashik Jawahar and Pasonen-Seppanen, Sanna and Heikkinen, Sami and Lalowski, Maciej and Karna, Riikka and Harkonen, Kai and Makinen, Petri and Lazaro-Ibanez, Elisa and Siljander, Pia R-M and Oikari, Sanna and Levonen, Anna-Liisa and Rilla, Kirsi | 10.1007/s00018-019-03399-5 EA DEC 2019 | HYALURONAN SYNTHASE;TRANSCRIPTION FACTORS;PANCREATIC-CANCER;O-GLCNACYLATION;SHEDDING LIGHT;TUMOR-CELLS;PROLIFERATION;ACTIVATION;CYCLE;MICROVESICLES |
| Immunoaffinity-Based Isolation of Melanoma Cell-Derived and T Cell-Derived Exosomes from Plasma of Melanoma Patients | MELANOMA: Methods and Protocols; 2021 | Mondal, SK and Whiteside, TL and Mondal, Sujan Kumar and Whiteside, Theresa L. | 10.1007/978-1-0716-1205-7_23 D2 10.1007/978-1-0716-1205-7 | ANTIBODY;ANTIGEN |
| Small RNA deep sequencing discriminates subsets of extracellular vesicles released by melanoma cells - Evidence of unique microRNA cargos | RNA BIOLOGY; 2015 | Lunavat, TR and Cheng, L and Kim, DK and Bhadury, J and Jang, SC and LÃ¤sser, C and Sharples, RA and LÃ³pez, MD and Nilsson, J and Gho, YS and Hill, AF and LÃ¶tvall, J and Lunavat, Taral R. and Cheng, Lesley and Kim, Dae-Kyum and Bhadury, Joydeep and Jang, Su Chul and Lasser, Cecilia and Sharples, Robyn A. and Lopez, Marcela Davila and Nilsson, Jonas and Gho, Yong Song and Hill, Andrew F. and Lotvall, Jan | 10.1080/15476286.2015.1056975 | NONCODING RNAS;MESSENGER-RNAS;EXOSOMES;CANCER;MICROVESICLES;EXPRESSION;REVEALS;TRANSCRIPTOME;PROLIFERATION;PROGRESSION |
| Melanoma Cells Produce Large Vesicular-Bodies That Cause Rapid Disruption of Brain Endothelial Barrier-Integrity and Disassembly of Junctional Proteins | INTERNATIONAL JOURNAL OF MOLECULAR SCIENCES; 2023 | Spurling, D and Anchan, A and Hucklesby, J and Finlay, G and Angel, CE and Graham, ES and Spurling, Dayna and Anchan, Akshata and Hucklesby, James and Finlay, Graeme and Angel, Catherine E. and Graham, E. Scott | 10.3390/ijms24076082 | CIRCULATING TUMOR-CELLS;EXTRACELLULAR VESICLES;TRANSENDOTHELIAL MIGRATION;BREAST-CANCER;METASTASIS;EXOSOMES;MICROVESICLES;DIAPEDESIS;SURVIVAL;CADHERIN |
| Extracellular vesicles shed by melanoma cells contain a modified form of H1.0 linker histone and H1.0 mRNA-binding proteins | INTERNATIONAL JOURNAL OF ONCOLOGY; 2016 | Schiera, G and Di Liegro, CM and Puleo, V and Colletta, O and Fricano, A and Cancemi, P and Di Cara, G and Di Liegro, I and Schiera, Gabriella and Di Liegro, Carlo Maria and Puleo, Veronica and Colletta, Oriana and Fricano, Anna and Cancemi, Patrizia and Di Cara, Gianluca and Di Liegro, Italia | 10.3892/ijo.2016.3692 | MEMBRANE-VESICLES;H1-DEGREES;IDENTIFICATION;TRANSCRIPTION;EXOSOMES;RELEASE;TISSUES;PIPPIN;BRAIN |
| MALDI Detection of Exosomes: A Potential Tool for Cancer Studies | CHEM; 2019 | Zhu, YD and Pick, H and Gasilova, N and Li, XY and Lin, TE and Laeubli, HP and Zippelius, A and Ho, PC and Girault, HH and Zhu, Yingdi and Pick, Horst and Gasilova, Natalia and Li, Xiaoyun and Lin, Tzu-En and Laeubli, Heinz Philipp and Zippelius, Alfred and Ho, Ping-Chih and Girault, Hubert H. | 10.1016/j.chempr.2019.04.007 | C-REACTIVE PROTEIN;EXTRACELLULAR VESICLES;MASS-SPECTROMETRY;LIQUID BIOPSY;MELANOMA-CELLS;PROGRESSION;MARKER;EXPRESSION;IDENTIFICATION;MICROVESICLES |
| Extracellular vesicle-dependent effect of RNA-binding protein IGF2BP1 on melanoma metastasis | ONCOGENE; 2019 | Ghoshal, A and Rodrigues, LC and Gowda, CP and Elcheva, IA and Liu, ZQ and Abraham, T and Spiegelman, VS and Ghoshal, Archita and Rodrigues, Lucas C. and Gowda, Chethana P. and Elcheva, Irina A. and Liu, Zhenqiu and Abraham, Thomas and Spiegelman, Vladimir S. | 10.1038/s41388-019-0797-3 | MYC MESSENGER-RNA;CRD-BP;CODING REGION;C-MYC;NICHE FORMATION;EXPRESSION;CELLS;TRANSCRIPTION;BETA-TRCP1;PHENOTYPE |
| Tracking extracellular vesicle phenotypic changes enables treatment monitoring in melanoma | SCIENCE ADVANCES; 2020 | Wang, J and Wuethrich, A and Ibn Sina, A and Lane, RE and Lin, LL and Wang, YL and Cebon, J and Behren, A and Trau, M and Wang, Jing and Wuethrich, Alain and Ibn Sina, Abu Ali and Lane, Rebecca E. and Lin, Lynlee L. and Wang, Yuling and Cebon, Jonathan and Behren, Andreas and Trau, Matt | 10.1126/sciadv.aax3223 | BREAST-CANCER;EXOSOMES;MICROVESICLES;PROTEIN;CELLS |
| Inhibition of extracellular vesicle-derived miR-146a-5p decreases progression of melanoma brain metastasis via Notch pathway dysregulation in astrocytes | JOURNAL OF EXTRACELLULAR VESICLES; 2023 | Rigg, E and Wang, JW and Xue, ZW and Lunavat, TR and Liu, GW and Hoang, T and Parajuli, H and Han, MZ and Bjerkvig, R and Nazarov, PV and Nicot, N and Kreis, S and Margue, C and Nomigni, MT and Utikal, J and Miletic, H and Sundstrom, T and Ystaas, LAR and Li, XA and Thorsen, F and Rigg, Emma and Wang, Jiwei and Xue, Zhiwei and Lunavat, Taral R. and Liu, Guowei and Hoang, Tuyen and Parajuli, Himalaya and Han, Mingzhi and Bjerkvig, Rolf and Nazarov, Petr V. and Nicot, Nathalie and Kreis, Stephanie and Margue, Christiane and Nomigni, Milene Tetsi and Utikal, Jochen and Miletic, Hrvoje and Sundstrom, Terje and Ystaas, Lars A. R. and Li, Xingang and Thorsen, Frits | 10.1002/jev2.12363 | MONOCYTE-CHEMOATTRACTANT PROTEIN-1;CELLS;MICRORNAS;GROWTH |
| Size-exclusion chromatography combined with DIA-MS enables deep proteome profiling of extracellular vesicles from melanoma plasma and serum | CELLULAR AND MOLECULAR LIFE SCIENCES; 2024 | Lattmann, E and RÃ¤ss, L and Tognetti, M and GÃ³mez, JMM and Lapaire, V and Bruderer, R and Reiter, L and Feng, YH and Steinmetz, LM and Levesque, MP and Lattmann, Evelyn and Rass, Luca and Tognetti, Marco and Gomez, Julia M. Martinez and Lapaire, Valerie and Bruderer, Roland and Reiter, Lukas and Feng, Yuehan and Steinmetz, Lars M. and Levesque, Mitchell P. | 10.1007/s00018-024-05137-y | TRANSFER-RNA SYNTHETASES;EXOSOMES;MICROPARTICLES;ACTIVATION;MICRORNAS;INTEGRINS;CLEAVAGE;DISEASE;CSPG4;CELLS |
| Hsp70-containing extracellular vesicles are capable of activating of adaptive immunity in models of mouse melanoma and colon carcinoma | SCIENTIFIC REPORTS; 2021 | Komarova, EY and Suezov, RV and Nikotina, AD and Aksenov, ND and Garaeva, LA and Shtam, TA and Zhakhov, AV and Martynova, MG and Bystrova, OA and Istomina, MS and Ischenko, AM and Margulis, BA and Guzhova, IV and Komarova, Elena Y. and Suezov, Roman, V and Nikotina, Alina D. and Aksenov, Nikolay D. and Garaeva, Luiza A. and Shtam, Tatiana A. and Zhakhov, Alexander, V and Martynova, Marina G. and Bystrova, Olga A. and Istomina, Maria S. and Ischenko, Alexander M. and Margulis, Boris A. and Guzhova, Irina, V | 10.1038/s41598-021-00734-4 | HEAT-SHOCK PROTEINS;HEAT-SHOCK-PROTEIN-70 HSP70;PLASMA-MEMBRANE;CANCER-CELLS;IFN-GAMMA;EXOSOMES;IL-10;PROTECTION;GROWTH;IMMUNOTHERAPY |
| Extracellular vesicles powered cancer immunotherapy: Targeted delivery of adenovirus-based cancer vaccine in humanized melanoma model | JOURNAL OF CONTROLLED RELEASE; 2024 | Mathlouthi, S and Kuryk, L and Prygiel, M and Lupo, MG and Zasada, AA and Pesce, C and Ferri, N and Rinner, B and Salmaso, S and Garofalo, M and Mathlouthi, Sara and Kuryk, Lukasz and Prygiel, Marta and Lupo, Maria Giovanna and Zasada, Aleksandra Anna and Pesce, Cristiano and Ferri, Nicola and Rinner, Beate and Salmaso, Stefano and Garofalo, Mariangela | 10.1016/j.jconrel.2024.10.057 EA NOV 2024 | ONCOLYTIC VIRUSES;TUMOR;NRAS |
| Proteome characterization of melanoma exosomes reveals a specific signature for metastatic cell lines | PIGMENT CELL & MELANOMA RESEARCH; 2015 | Lazar, I and Clement, E and Ducoux-Petit, M and Denat, L and Soldan, V and Dauvillier, S and Balor, S and Burlet-Schiltz, O and Larue, L and Muller, C and Nieto, L and Lazar, Ikrame and Clement, Emily and Ducoux-Petit, Manuelle and Denat, Laurence and Soldan, Vanessa and Dauvillier, Stephanie and Balor, Stephanie and Burlet-Schiltz, Odile and Larue, Lionel and Muller, Catherine and Nieto, Laurence | 10.1111/pcmr.12380 | TUMOR-RELEASED MICROVESICLES;EXTRACELLULAR VESICLES;MEMBRANE-VESICLES;PROMOTE;CAVEOLIN-1;GROWTH |
| Procoagulant and immunogenic properties of melanoma exosomes, microvesicles and apoptotic vesicles | ONCOTARGET; 2016 | Muhsin-Sharafaldine, MR and Saunderson, SC and Dunn, AC and Faed, JM and Kleffmann, T and McLellan, AD and Muhsin-Sharafaldine, Morad-Remy and Saunderson, Sarah C. and Dunn, Amy C. and Faed, James M. and Kleffmann, Torsten and McLellan, Alexander D. | 10.18632/oncotarget.10783 | CELL-DERIVED EXOSOMES;TISSUE FACTOR;EXTRACELLULAR VESICLES;THROMBIN GENERATION;TUMOR-CELLS;SHOTGUN PROTEOMICS;PANCREATIC-CANCER;MEMBRANE-VESICLES;B-CELLS;MICROPARTICLES |
| Natural-Killer-Derived Extracellular Vesicles: Immune Sensors and Interactors | FRONTIERS IN IMMUNOLOGY; 2020 | Federici, C and Shahaj, E and Cecchetti, S and Camerini, S and Casella, M and Iessi, E and Camisaschi, C and Paolino, G and Calvieri, S and Ferro, S and Cova, A and Squarcina, P and Bertuccini, L and Iosis, F and Huber, V and Lugini, L and Federici, Cristina and Shahaj, Eriomina and Cecchetti, Serena and Camerini, Serena and Casella, Marialuisa and Iessi, Elisabetta and Camisaschi, Chiara and Paolino, Giovanni and Calvieri, Stefano and Ferro, Simona and Cova, Agata and Squarcina, Paola and Bertuccini, Lucia and Iosis, Francesca and Huber, Veronica and Lugini, Luana | 10.3389/fimmu.2020.00262 | NK CELLS;PERIPHERAL-BLOOD;EXOSOMES;IMMUNOTHERAPY;EXPRESSION;COAGULATION;LYMPHOCYTES;RECRUITMENT;MECHANISMS;BIOLOGY |
| Similarities and Differences in the Protein Composition of Cutaneous Melanoma Cells and Their Exosomes Identified by Mass Spectrometry | CANCERS; 2023 | Surman, M and Jankowska, U and Wilczak, M and Przybylo, M and Surman, Magdalena and Jankowska, Urszula and Wilczak, Magdalena and Przybylo, Malgorzata | 10.3390/cancers15041097 | ANNEXIN A1;TUMOR;GROWTH;METASTASIS;EXPRESSION;INDUCTION;MIGRATION;BIOMARKER;INVASION |
| Extracellular Vesicles Shedding Promotes Melanoma Growth in Response to Chemotherapy | SCIENTIFIC REPORTS; 2019 | Andrade, LND and Otake, AH and Cardim, SGB and da Silva, FIL and Sakamoto, MMI and Furuya, TK and Uno, M and Pasini, FS and Chammas, R and de Sousa Andrade, Luciana Nogueira and Otake, Andreia Hanada and Braga Cardim, Silvia Guedes and da Silva, Felipe I. Lelis and Ikoma Sakamoto, Mariana Mari and Furuya, Tatiane Katsue and Uno, Miyuki and Pasini, Fatima Solange and Chammas, Roger | 10.1038/s41598-019-50848-z | BREAST-CANCER CELLS;IN-VITRO;EXOSOMES;TUMOR;RESISTANCE;PHENOTYPE;ANGIOGENESIS;MICROENVIRONMENT;BIOCHEMOTHERAPY;MACROPHAGES |
| <i>In Vivo</i> Immunogenicity Screening of Tumor-Derived Extracellular Vesicles by Flow Cytometry of Splenic T Cells | JOVE-JOURNAL OF VISUALIZED EXPERIMENTS; 2021 | Stritzke, F and Poeck, H and Heidegger, S and Stritzke, Florian and Poeck, Hendrik and Heidegger, Simon | 10.3791/62811 | |
| Inorganic Nanoparticles Change Cancer-Cell-Derived Extracellular Vesicle Secretion Levels and Cargo Composition, Resulting in Secondary Biological Effects | ACS APPLIED MATERIALS & INTERFACES; 2024 | Buttiens, K and Maksoudian, C and Gilabert, IP and Luci, CR and Manshian, BB and Soenen, SJ and Buttiens, Kiana and Maksoudian, Christy and Gilabert, Irati Perez and Luci, Carla Rios and Manshian, Bella B. and Soenen, Stefaan J. | 10.1021/acsami.3c12680 | ALKALINE-PHOSPHATASE;TUMOR;PATHWAY;DISEASE;BIOGENESIS;EXOSOMES;NETWORK;INSULIN;GROWTH;LIGHT |
| Tracking the EMT-like phenotype switching during targeted therapy in melanoma by analyzing extracellular vesicle phenotypes | BIOSENSORS & BIOELECTRONICS; 2024 | Zhou, Q and Wang, J and Zhang, Z and Wuethrich, A and Lobb, RJ and Trau, M and Zhou, Quan and Wang, Jing and Zhang, Zhen and Wuethrich, Alain and Lobb, Richard J. and Trau, Matt | 10.1016/j.bios.2023.115819 EA NOV 2023 | MESENCHYMAL TRANSITION;RESISTANCE;EXPRESSION;BRAF;GENE |
| Investigating nano-sized tumor-derived extracellular vesicles in enhancing anti-PD-1 immunotherapy | NANOSCALE; 2024 | Abouali, H and Przedborski, M and Kohandel, M and Poudineh, M and Abouali, Hesam and Przedborski, Michelle and Kohandel, Mohammad and Poudineh, Mahla | 10.1039/d4nr00729h EA SEP 2024 | SQUAMOUS-CELL CARCINOMA;PATHWAY;PD-L1;ACTIVATION;EXOSOMES;MELANOMA;HEAD;NECK |
| Metabolic reprogramming of stromal fibroblasts by melanoma exosome microRNA favours a pre-metastatic microenvironment | SCIENTIFIC REPORTS; 2018 | La Shu, S and Yang, YC and Allen, CL and Maguire, O and Minderman, H and Sen, A and Ciesielski, MJ and Collins, KA and Bush, PJ and Singh, P and Wang, X and Morgan, M and Qu, J and Bankert, RB and Whiteside, TL and Wu, Y and Ernstoff, MS and La Shu, Shin and Yang, Yunchen and Allen, Cheryl L. and Maguire, Orla and Minderman, Hans and Sen, Arindam and Ciesielski, Michael J. and Collins, Katherine A. and Bush, Peter J. and Singh, Prashant and Wang, Xue and Morgan, Martin and Qu, Jun and Bankert, Richard B. and Whiteside, Theresa L. and Wu, Yun and Ernstoff, Marc S. | 10.1038/s41598-018-31323-7 | BIOLOGICALLY-ACTIVE EXOSOMES;BREAST-CANCER CELLS;TUMOR MICROENVIRONMENT;EXTRACELLULAR VESICLES;PROGRESSION;INDUCE;PH;TRANSITION;EXTRACTION;PHENOTYPE |
| Melanoma Extracellular Vesicles Generate Immunosuppressive Myeloid Cells by Upregulating PD-L1 via TLR4 Signaling | CANCER RESEARCH; 2019 | Fleming, V and Hu, XY and Weller, C and Weber, R and Groth, C and Riester, Z and HÃ¼ser, L and Sun, Q and Nagibin, V and Kirschning, C and Bronte, V and Utikal, J and Altevogt, P and Umansky, V and Fleming, Viktor and Hu, Xiaoying and Weller, Celine and Weber, Rebekka and Groth, Christopher and Riester, Zeno and Hueser, Laura and Sun, Qian and Nagibin, Vasyl and Kirschning, Carsten and Bronte, Vincenzo and Utikal, Jochen and Altevogt, Peter and Umansky, Viktor | 10.1158/0008-5472.CAN-19-0053 | RET TRANSGENIC MICE;SUPPRESSOR-CELLS;EXOSOMES;MICROVESICLES;INDUCTION;MICRORNAS;PHENOTYPE;MELATONIN;PATHWAY;PLASMA |
| Cellular Vesicles: New Insights in Engineering Methods, Interaction with Cells and Potential for Brain Targeting | JOURNAL OF PHARMACOLOGY AND EXPERIMENTAL THERAPEUTICS; 2019 | Marazioti, A and Papadia, K and Kannavou, M and Spella, M and Basta, A and de Lastic, AL and Rodi, M and Mouzaki, A and Samiotaki, M and Panayotou, G and Stathopoulos, GT and Antimisiaris, SG and Marazioti, A. and Papadia, K. and Kannavou, M. and Spella, M. and Basta, A. and de Lastic, A-L and Rodi, M. and Mouzaki, A. and Samiotaki, M. and Panayotou, G. and Stathopoulos, G. T. and Antimisiaris, S. G. | 10.1124/jpet.119.257097 | EXOSOME-MIMETIC NANOVESICLES;DRUG-DELIVERY SYSTEMS;EXTRACELLULAR VESICLES;BARRIER MODEL;LIPOSOMES;LOCALIZATION;NANOCARRIERS;GENERATION;INTEGRITY;HCMEC/D3 |
| Platelet-derived extracellular vesicles induced through different activation pathways drive melanoma progression by functional and transcriptional changes | CELL COMMUNICATION AND SIGNALING; 2024 | Tavukcuoglu, Z and Butt, U and de Faria, AVS and Oesterreicher, J and Holnthoner, W and Laitinen, S and Palviainen, M and Siljander, PRM and Tavukcuoglu, Zeynep and Butt, Umar and de Faria, Alessandra V. Sousa and Oesterreicher, Johannes and Holnthoner, Wolfgang and Laitinen, Saara and Palviainen, Mari and Siljander, Pia R-M | 10.1186/s12964-024-01973-4 | IN-VITRO;METASTASIS;CANCER;EXPRESSION;FUCOIDAN;CELLS |
| Preservation of exosomes at room temperature using lyophilization | INTERNATIONAL JOURNAL OF PHARMACEUTICS; 2018 | Charoenviriyakul, C and Takahashi, Y and Nishikawa, M and Takakura, Y and Charoenviriyakul, Chonlada and Takahashi, Yuki and Nishikawa, Makiya and Takakura, Yoshinobu | 10.1016/j.ijpharm.2018.10.032 | EXTRACELLULAR VESICLES;DELIVERY VEHICLES;STABILITY;PROTEINS;CELLS;SIRNA;CRYOPROTECTANTS;FORMULATION;LIPOSOMES;TRACKING |
| Exosomal microRNA-4535 of Melanoma Stem Cells Promotes Metastasis by Inhibiting Autophagy Pathway | STEM CELL REVIEWS AND REPORTS; 2023 | Liu, DD and Li, XS and Zeng, B and Zhao, QT and Chen, H and Zhang, YH and Chen, YT and Wang, JY and Xing, HR and Liu, Doudou and Li, Xiaoshuang and Zeng, Bin and Zhao, Qiting and Chen, Hao and Zhang, Yuhan and Chen, Yuting and Wang, Jianyu and Xing, H. Rosie | 10.1007/s12015-022-10358-4 EA MAR 2022 | EXTRACELLULAR VESICLES;ANALYSIS REVEALS;TUMOR-CELLS;CANCER;SURVIVAL;DORMANCY |
| Novel lncRNA Gm33149 modulates metastatic heterogeneity in melanoma by regulating the miR-5623-3p/Wnt axis via exosomal transfer | CANCER GENE THERAPY; 2024 | Chen, Y and Zhang, YH and Li, J and Shi, L and Xie, JC and Han, X and Chen, YT and Xiang, M and Li, BW and Xing, HR and Wang, JY and Chen, Yan and Zhang, Yu-Han and Li, Jie and Shi, Lei and Xie, Jia-Cheng and Han, Xue and Chen, Yu-Ting and Xiang, Meng and Li, Bo-Wen and Xing, H. Rosie and Wang, Jian-Yu | 10.1038/s41417-023-00707-x EA DEC 2023 | CANCER;PATHWAY;PD-L1;CELLS |
| Extracellular vesicles originating from melanoma cells promote dysregulation in haematopoiesis as a component of cancer immunoediting | JOURNAL OF EXTRACELLULAR VESICLES; 2024 | Mamand, DR and Bazaz, S and Mohammad, DK and Liang, XM and Pavlova, S and Mim, C and Gabrielsson, S and Nordin, JZ and Wiklander, OPB and Abedi-Valugerdi, M and EL-Andaloussi, S and Mamand, Doste R. and Bazaz, Safa and Mohammad, Dara K. and Liang, Xiuming and Pavlova, Svetlana and Mim, Carsten and Gabrielsson, Susanne and Nordin, Joel Z. and Wiklander, Oscar P. B. and Abedi-Valugerdi, Manuchehr and EL-Andaloussi, Samir | 10.1002/jev2.12471 | ROLES;TUMOR;VEGF |
| <i>In Vivo</i> Visualized Tracking of Tumor-Derived Extracellular Vesicles Using CRISPR-Cas9 System | TECHNOLOGY IN CANCER RESEARCH & TREATMENT; 2022 | Ye, YY and Shi, Q and Yang, T and Xie, F and Zhang, X and Xu, B and Fang, JW and Chen, JN and Zhang, YJ and Li, J and Ye, Yangyang and Shi, Qian and Yang, Ting and Xie, Fei and Zhang, Xiang and Xu, Bin and Fang, Jingwen and Chen, Jiangning and Zhang, Yujing and Li, Jing | 10.1177/15330338221085370 | METASTASIS;EXOSOMES;COMPLEX;NICHE;CELLS;GUIDE;RNA |
| Melanoma Affects the Composition of Blood Cell-Derived Extracellular Vesicles | FRONTIERS IN IMMUNOLOGY; 2016 | Koliha, N and Heider, U and Ozimkowski, T and Wiemann, M and Bosio, A and Wild, S and Koliha, Nina and Heider, Ute and Ozimkowski, Tobias and Wiemann, Martin and Bosio, Andreas and Wild, Stefan | 10.3389/fimmu.2016.00282 | EXOSOMAL-LIKE VESICLES;T-CELLS;CIRCULATING MICROPARTICLES;MEMBRANE-VESICLES;DENDRITIC CELLS;MICROVESICLES;RELEASE;SURFACE;ACTIVATION;GROWTH |
| Pre-metastatic cancer exosomes induce immune surveillance by patrolling monocytes at the metastatic niche | NATURE COMMUNICATIONS; 2017 | Plebanek, MP and Angeloni, NL and Vinokour, E and Li, J and Henkin, A and Martinez-Marin, D and Filleur, S and Bhowmick, R and Henkin, J and Miller, SD and Ifergan, I and Lee, Y and Osman, I and Thaxton, CS and Volpert, OV and Plebanek, Michael P. and Angeloni, Nicholas L. and Vinokour, Elena and Li, Jia and Henkin, Anna and Martinez-Marin, Dalia and Filleur, Stephanie and Bhowmick, Reshma and Henkin, Jack and Miller, Stephen D. and Ifergan, Igal and Lee, Yesung and Osman, Iman and Thaxton, C. Shad and Volpert, Olga V. | 10.1038/s41467-017-01433-3 | EPITHELIUM-DERIVED FACTOR;EXTRACELLULAR VESICLES;TUMOR MICROENVIRONMENT;TRANSCRIPTION FACTOR;CELLS;MICROVESICLES;BIOGENESIS;INHIBITION;MEDIATORS;DISEASE |
| Oncolytic alphavirus-induced extracellular vesicles counteract the immunosuppressive effect of melanoma-derived extracellular vesicles | SCIENTIFIC REPORTS; 2025 | Bhatt, DK and Boerma, A and Bustos, SO and Otake, AH and Carrasco, AGM and Reis, PP and Chammas, R and Daemen, T and Andrade, LND and Bhatt, Darshak K. and Boerma, Annemarie and Bustos, Silvina Odete and Otake, Andreia Hanada and Carrasco, Alexis German Murillo and Reis, Patricia Pintor and Chammas, Roger and Daemen, Toos and Andrade, Luciana Nogueira de Sousa | 10.1038/s41598-024-82331-9 | EXOSOMES;TUMOR;BIOGENESIS;IMMUNITY |
| P2X7 promotes metastatic spreading and triggers release of miRNA-containing exosomes and microvesicles from melanoma cells | CELL DEATH & DISEASE2021 | Pegoraro, A and De Marchi, E and Ferracin, M and Orioli, E and Zanoni, M and Bassi, C and Tesei, A and Capece, M and Dika, E and Negrini, M and Di Virgilio, F and Adinolfi, E and Pegoraro, Anna and De Marchi, Elena and Ferracin, Manuela and Orioli, Elisa and Zanoni, Michele and Bassi, Cristian and Tesei, Anna and Capece, Marina and Dika, Emi and Negrini, Massimo and Di Virgilio, Francesco and Adinolfi, Elena | 10.1038/s41419-021-04378-0 | EXTRACELLULAR VESICLES;P2X(7) RECEPTOR;ATP;INVASION;PROLIFERATION;STIMULATION;CHANNELS |
| SCS macrophages suppress melanoma by restricting tumor-derived vesicle-B cell interactions | SCIENCE; 2016 | Pucci, F and Garris, C and Lai, CP and Newton, A and Pfirschke, C and Engblom, C and Alvarez, D and Sprachman, M and Evavold, C and Magnuson, A and von Andrian, UH and Glatz, K and Breakefield, XO and Mempel, TR and Weissleder, R and Pittet, MJ and Pucci, Ferdinando and Garris, Christopher and Lai, Charles P. and Newton, Andita and Pfirschke, Christina and Engblom, Camilla and Alvarez, David and Sprachman, Melissa and Evavold, Charles and Magnuson, Angela and von Andrian, Ulrich H. and Glatz, Katharina and Breakefield, Xandra O. and Mempel, Thorsten R. and Weissleder, Ralph and Pittet, Mikael J. | 10.1126/science.aaf1328 | SUBCAPSULAR SINUS MACROPHAGES;LYMPH-NODES;INFLAMMATION;EXOSOMES;MICROVESICLES;INFECTION;RNA |
| Exosome-Containing Preparations From Postirradiated Mouse Melanoma Cells Delay Melanoma Growth In Vivo by a Natural Killer Cell-Dependent Mechanism | INTERNATIONAL JOURNAL OF RADIATION ONCOLOGY BIOLOGY PHYSICS; 2020 | Jella, KK and Nasti, TH and Li, ZT and Lawson, DH and Switchenko, JM and Ahmed, R and Dynan, WS and Khan, MK and Jella, Kishore Kumar and Nasti, Tahseen H. and Li, Zhentian and Lawson, David H. and Switchenko, Jeffrey M. and Ahmed, Rafi and Dynan, William S. and Khan, Mohammad K. | 10.1016/j.ijrobp.2020.06.016 | HEAT-SHOCK PROTEINS;CANCER-CELLS;RADIATION;MICROENVIRONMENT;SECRETION;RELEASE;PATHWAY;BIOLOGY;INNATE;TSG101 |
| TGF-Î² mRNA levels in circulating extracellular vesicles are associated with response to anti-PD1 treatment in metastatic melanoma | FRONTIERS IN MOLECULAR BIOSCIENCES; 2024 | Crucitta, S and Cucchiara, F and Marconcini, R and Bulleri, A and Manacorda, S and Capuano, A and Cioni, D and Nuzzo, A and de Jonge, E and Mathjissen, RHJ and Neri, E and van Schaik, RHN and Fogli, S and Danesi, R and Del Re, M and Crucitta, Stefania and Cucchiara, Federico and Marconcini, Riccardo and Bulleri, Alessandra and Manacorda, Simona and Capuano, Annalisa and Cioni, Dania and Nuzzo, Amedeo and de Jonge, Evert and Mathjissen, Ron H. J. and Neri, Emanuele and van Schaik, Ron H. N. and Fogli, Stefano and Danesi, Romano and Del Re, Marzia | 10.3389/fmolb.2024.1288677 | GROWTH-FACTOR-BETA;IMMUNE CHECKPOINT INHIBITORS;PD-L1 EXPRESSION;RESISTANCE;PLASMA;NIVOLUMAB;GAMMA;PEMBROLIZUMAB;IPILIMUMAB;BIOMARKERS |
| <i>In Vivo</i> Flow Cytometry of Circulating Tumor-Associated Exosomes | ANALYTICAL CELLULAR PATHOLOGY; 2016 | Nolan, J and Sarimollaoglu, M and Nedosekin, DA and Jamshidi-Parsian, A and Galanzha, EI and Kore, RA and Griffin, RJ and Zharov, VP and Nolan, Jacqueline and Sarimollaoglu, Mustafa and Nedosekin, Dmitry A. and Jamshidi-Parsian, Azemat and Galanzha, Ekaterina I. and Kore, Rajshekhar A. and Griffin, Robert J. and Zharov, Vladimir P. | 10.1155/2016/1628057 | EXTRACELLULAR VESICLES;CANCER METASTASIS;LIQUID BIOPSY;CELLS;MICROPARTICLES;MICROVESICLES;BIOMARKERS;TRACKING |
| Efficient extracellular vesicle isolation by combining cell media modifications, ultrafiltration, and size-exclusion chromatography | PLOS ONE; 2018 | Guerreiro, EM and Vestad, B and Steffensen, LA and Aass, HCD and Saeed, M and Ovstebo, R and Costea, DE and Galtung, HK and Soland, TM and Guerreiro, Eduarda M. and Vestad, Beate and Steffensen, Lilly Alice and Aass, Hans Christian D. and Saeed, Muhammad and Ovstebo, Reidun and Costea, Daniela Elena and Galtung, Hilde Kanli and Soland, Tine M. | 10.1371/journal.pone.0204276 | EXOSOMES;CULTURE;MICROVESICLES;TRACKING;GROWTH;SERA;RNAS |
| Development of a rapid lateral flow immunoassay test for detection of exosomes previously enriched from cell culture medium and body fluids | JOURNAL OF EXTRACELLULAR VESICLES; 2016 | Oliveira-RodrÃ­guez, M and LÃ³pez-Cobo, S and Reyburn, HT and Costa-GarcÃ­a, A and LÃ³pez-MartÃ­n, S and YÃ¡Ã±ez-MÃ³, M and Cernuda-MorollÃ³n, E and Paschen, A and ValÃ©s-GÃ³mez, M and Blanco-LÃ³pez, MC and Oliveira-Rodriguez, Myriam and Lopez-Cobo, Sheila and Reyburn, Hugh T. and Costa-Garcia, Agustin and Lopez-Martin, Soraya and Yanez-Mo, Maria and Cernuda-Morollon, Eva and Paschen, Annette and Vales-Gomez, Mar and Carmen Blanco-Lopez, Maria | 10.3402/jev.v5.31803 | EXTRACELLULAR VESICLES;CIRCULATING EXOSOMES;TETRASPANINS;MICROVESICLES;DIAGNOSTICS;PLASMA;ASSAY |
| Exosomal PD-L1 contributes to immunosuppression and is associated with anti-PD-1 response | NATURE; 2018 | Chen, G and Huang, AC and Zhang, W and Zhang, G and Wu, M and Xu, W and Yu, ZL and Yang, JG and Wang, BK and Sun, HH and Xia, HF and Man, QW and Zhong, WQ and Antelo, LF and Wu, B and Xiong, XP and Liu, XM and Guan, L and Li, T and Liu, SJ and Yang, RF and Lu, YT and Dong, LY and McGettigan, S and Somasundaram, R and Radhakrishnan, R and Mills, G and Lu, YL and Kim, J and Chen, YHH and Dong, HD and Zhao, YF and Karakousis, GC and Mitchell, TC and Schuchter, LM and Herlyn, M and Wherry, EJ and Xu, XW and Guo, W and Chen, Gang and Huang, Alexander C. and Zhang, Wei and Zhang, Gao and Wu, Min and Xu, Wei and Yu, Zili and Yang, Jiegang and Wang, Beike and Sun, Honghong and Xia, Houfu and Man, Qiwen and Zhong, Wenqun and Antelo, Leonardo F. and Wu, Bin and Xiong, Xuepeng and Liu, Xiaoming and Guan, Lei and Li, Ting and Liu, Shujing and Yang, Ruifeng and Lu, Youtao and Dong, Liyun and McGettigan, Suzanne and Somasundaram, Rajasekharan and Radhakrishnan, Ravi and Mills, Gordon and Lu, Yiling and Kim, Junhyong and Chen, Youhai H. and Dong, Haidong and Zhao, Yifang and Karakousis, Giorgos C. and Mitchell, Tara C. and Schuchter, Lynn M. and Herlyn, Meenhard and Wherry, E. John and Xu, Xiaowei and Guo, Wei | 10.1038/s41586-018-0392-8 | INDUCE APOPTOSIS;T-CELLS;BLOCKADE;PEMBROLIZUMAB;RESISTANCE;MECHANISM;BIOMARKER;VESICLES;PLASMA;CD8(+) |
| Single-cell analysis of a tumor-derived exosome signature correlates with prognosis and immunotherapy response | JOURNAL OF TRANSLATIONAL MEDICINE; 2021 | Wu, JN and Zeng, DQ and Zhi, SM and Ye, ZL and Qiu, WJ and Huang, N and Sun, L and Wang, CL and Wu, ZZ and Bin, JP and Liao, YL and Shi, M and Liao, WJ and Wu, Jiani and Zeng, Dongqiang and Zhi, Shimeng and Ye, Zilan and Qiu, Wenjun and Huang, Na and Sun, Li and Wang, Chunlin and Wu, Zhenzhen and Bin, Jianping and Liao, Yulin and Shi, Min and Liao, Wangjun | 10.1186/s12967-021-03053-4 | CANCER EXOSOMES;EGFR MUTATIONS;LUNG-CANCER;VESICLES;GENE |
| Cancer-derived exosomes trigger endothelial to mesenchymal transition followed by the induction of cancer-associated fibroblasts | ACTA BIOMATERIALIA; 2018 | Yeon, JH and Jeong, HE and Seo, H and Cho, S and Kim, K and Na, D and Chung, S and Park, J and Choi, N and Kang, JY and Yeon, Ju Hun and Jeong, Hyo Eun and Seo, Hyemin and Cho, Siwoo and Kim, Kimin and Na, Dokyun and Chung, Seok and Park, Jaesung and Choi, Nakwon and Kang, Ji Yoon | 10.1016/j.actbio.2018.07.001 | CARCINOMA-ASSOCIATED FIBROBLASTS;TUMOR-GROWTH;MYOFIBROBLAST DIFFERENTIATION;INTERSTITIAL FLOW;CELL;DISEASE;PROLIFERATION;ANGIOGENESIS;PLASTICITY;MIGRATION |
| Tumor-derived exosomes promote the in vitro osteotropism of melanoma cells by activating the SDF-1/CXCR4/CXCR7 axis | JOURNAL OF TRANSLATIONAL MEDICINE; 2019 | Mannavola, F and Tucci, M and Felici, C and Passarelli, A and D'Oronzo, S and Silvestris, F and Mannavola, Francesco and Tucci, Marco and Felici, Claudia and Passarelli, Anna and D'Oronzo, Stella and Silvestris, Francesco | 10.1186/s12967-019-1982-4 | EPITHELIAL-MESENCHYMAL TRANSITION;BONE METASTASES;PIVOTAL ROLE;MIGRATION;PHENOTYPE;SURVIVAL;MARROW;CXCL12 |
| Analysis of cancer-related mutations in extracellular vesicles RNA by Droplet Digitalâ„¢ PCR | BIOTECHNIQUES; 2020 | Yap, SA and MÃ¼nster-Wandowski, A and Nonnenmacher, A and Keilholz, U and Liebs, S and Yap, Soo Ann and Muenster-Wandowski, Agnieszka and Nonnenmacher, Anika and Keilholz, Ulrich and Liebs, Sandra | 10.2144/btn-2020-0028 | LIQUID BIOPSY;MEMBRANE-VESICLES;NUCLEIC-ACIDS;EXOSOMES;DNA;KRAS;BRAF;MICROVESICLES;PROGRESSION;INHIBITION |
| Purity and yield of melanoma exosomes are dependent on isolation method | JOURNAL OF EXTRACELLULAR VESICLES; 2020 | Shu, SL and Yang, YC and Allen, CL and Hurley, E and Tung, KH and Minderman, H and Wu, Y and Ernstoff, MS and Shu, Shin La and Yang, Yunchen and Allen, Cheryl L. and Hurley, Edward and Tung, Kaity H. and Minderman, Hans and Wu, Yun and Ernstoff, Marc S. | 10.1080/20013078.2019.1692401 | DENSITY-GRADIENT SEPARATION;CELL-DERIVED EXOSOMES;EXTRACELLULAR VESICLES;INDUCE APOPTOSIS;TUMOR-GROWTH;EXPRESSION;CYTOKINES;ULTRAFILTRATION;CHROMATOGRAPHY;INTERLEUKIN-8 |
| Comparative gene expression analysis in melanocytes driven by tumor cell-derived exosomes | EXPERIMENTAL CELL RESEARCH; 2020 | Xiao, DY and Li, XH and Rouchka, EC and Waigel, S and Zacharias, W and McMasters, KM and Hao, HY and Xiao, Deyi and Li, Xiaohong and Rouchka, Eric C. and Waigel, Sabine and Zacharias, Wolfgang and McMasters, Kelly M. and Hao, Hongying | 10.1016/j.yexcr.2019.111690 | EXTRACELLULAR VESICLES;ONTOLOGY;TOOL;ID3 |
| Exosome-mediated transfer of miR-222 is sufficient to increase tumor malignancy in melanoma | JOURNAL OF TRANSLATIONAL MEDICINE; 2016 | Felicetti, F and De Feo, A and Coscia, C and Puglisi, R and Pedini, F and Pasquini, L and Bellenghi, M and Errico, MC and Pagani, E and CarÃ¨, A and Felicetti, Federica and De Feo, Alessandra and Coscia, Carolina and Puglisi, Rossella and Pedini, Francesca and Pasquini, Luca and Bellenghi, Maria and Errico, Maria Cristina and Pagani, Elena and Care, Alessandra | 10.1186/s12967-016-0811-2 | PROGNOSTIC-SIGNIFICANCE;EXTRACELLULAR VESICLES;CANCER-CELLS;ACTIVATION;MICRORNAS;COMPLEX;PROGRESSION;PLASMA;GROWTH;CD63 |
| Extracellular vesicles released by melanocytes after UVA irradiation promote intercellular signaling via miR21 | PIGMENT CELL & MELANOMA RESEARCH; 2020 | WÃ¤ster, P and Eriksson, I and Vainikka, L and Ã–llinger, K and Waster, Petra and Eriksson, Ida and Vainikka, Linda and Ollinger, Karin | 10.1111/pcmr.12860 EA JAN 2020 | MICRORNA-21 PROMOTES;LYSOSOMAL EXOCYTOSIS;PLASMA-MEMBRANE;E-CADHERIN;EXPRESSION;MELANOMA;ULTRAVIOLET;DIFFERENTIATION;PROLIFERATION;KERATINOCYTES |
| Extracellular vesicles or free circulating DNA: where to search for BRAF and cKIT mutations? | NANOMEDICINE-NANOTECHNOLOGY BIOLOGY AND MEDICINE; 2018 | Klump, J and Phillipp, U and Follo, M and Eremin, A and Lehmann, H and Nestel, S and von Bubnoff, N and Nazarenko, I and Klump, Jennifer and Phillipp, Ulrike and Follo, Marie and Eremin, Anna and Lehmann, Hannes and Nestel, Sigrun and von Bubnoff, Nikolas and Nazarenko, Irina | 10.1016/j.nano.2017.12.009 | METASTATIC COLORECTAL-CANCER;PANCREATIC-CANCER;LIQUID BIOPSY;LUNG-CANCER;TUMOR DNA;EXOSOMES;KRAS;IDENTIFICATION;BIOMARKER;CETUXIMAB |
| Accelerated growth of B16BL6 tumor in mice through efficient uptake of their own exosomes by B16BL6 cells | CANCER SCIENCE; 2017 | Matsumoto, A and Takahashi, Y and Nishikawa, M and Sano, K and Morishita, M and Charoenviriyakul, C and Saji, H and Takakura, Y and Matsumoto, Akihiro and Takahashi, Yuki and Nishikawa, Makiya and Sano, Kohei and Morishita, Masaki and Charoenviriyakul, Chonlada and Saji, Hideo and Takakura, Yoshinobu | 10.1111/cas.13310 | NEUTRAL SPHINGOMYELINASE;INTRAVENOUS-INJECTION;DRUG DISCOVERY;PROGRESSION;METASTASIS;SURVIVIN;PATHWAY;PROTEIN;PROLIFERATION;CLEARANCE |
| Pre-analytical factors affecting the establishment of a single tube assay for multiparameter liquid biopsy detection in melanoma patients | MOLECULAR ONCOLOGY; 2020 | Schneegans, S and LÃ¼ck, L and Besler, K and Bluhm, L and Stadler, JC and Staub, J and Greinert, R and Volkmer, B and Kubista, M and Gebhardt, C and Sartori, A and Irwin, D and Serkkola, E and HÃ¤llstrÃ¶m, T and Lianidou, E and Sprenger-Haussels, M and Hussong, M and Mohr, P and Schneider, SW and Shaffer, J and Pantel, K and Wikman, H and Schneegans, Svenja and Lueck, Lelia and Besler, Katharina and Bluhm, Leonie and Stadler, Julia-Christina and Staub, Janina and Greinert, Ruediger and Volkmer, Beate and Kubista, Mikael and Gebhardt, Christoffer and Sartori, Alexander and Irwin, Darryl and Serkkola, Elina and Hallstrom, Taija and Lianidou, Evi and Sprenger-Haussels, Markus and Hussong, Melanie and Mohr, Peter and Schneider, Stefan W. and Shaffer, Jonathan and Pantel, Klaus and Wikman, Harriet | 10.1002/1878-0261.12669 EA APR 2020 | CIRCULATING TUMOR-CELLS;CANCER;DNA;MICRORNAS;NORMALIZATION;HETEROGENEITY;COLLECTION;EXPRESSION |
| Growth Hormone Upregulates Melanoma Drug Resistance and Migration via Melanoma-Derived Exosomes | CANCERS; 2024 | Kulkarni, P and Basu, R and Bonn, T and Low, B and Mazurek, N and Kopchick, JJ and Kulkarni, Prateek and Basu, Reetobrata and Bonn, Taylor and Low, Beckham and Mazurek, Nathaniel and Kopchick, John J. | 10.3390/cancers16152636 | EPITHELIAL-MESENCHYMAL TRANSITION;MAMMARY-CARCINOMA CELLS;CANCER-CELLS;STIMULATES ONCOGENICITY;MULTIDRUG-RESISTANCE;P-GLYCOPROTEIN;EXPRESSION;RECEPTOR;LINES;SENSITIVITY |
| Exosomes are secreted at similar densities by M21 and PC3 human cancer cells and show paclitaxel solubility | BIOCHIMICA ET BIOPHYSICA ACTA-BIOMEMBRANES; 2022 | Fisher, WS and Tchounwou, C and Wei, SP and Roberts, L and Ewert, KK and Safinya, CR and Fisher, William S. and Tchounwou, Christine and Wei, Sophia and Roberts, Logan and Ewert, Kai K. and Safinya, Cyrus R. | 10.1016/j.bbamem.2021.183841 EA JAN 2022 | LOADED CATIONIC LIPOSOMES;BLOOD-BRAIN-BARRIER;DRUG-DELIVERY;TRANSFERRIN RECEPTOR;VESICLES;MICROVESICLES;CYTOTOXICITY;MATURATION;EFFICIENCY;MICRORNAS |
| Oncolytic viruses alter the biogenesis of tumor extracellular vesicles and influence their immunogenicity | MOLECULAR THERAPY ONCOLOGY; 2024 | Hirigoyen, U and Guilbaud, C and Krejbich, M and Fouet, M and Fresquet, J and Arnaud, B and Com, E and Pineau, C and Cadiou, G and Burlaud-Gaillard, J and Erbs, P and Fradin, D and LabarriÃ¨re, N and Fonteneau, JF and Petithomme, T and Boisgerault, N and Hirigoyen, Ugo and Guilbaud, Coraly and Krejbich, Morgane and Fouet, Morgane and Fresquet, Judith and Arnaud, Bastien and Com, Emmanuelle and Pineau, Charles and Cadiou, Gwenann and Burlaud-Gaillard, Julien and Erbs, Philippe and Fradin, Delphine and Labarriere, Nathalie and Fonteneau, Jean-Francois and Petithomme, Tacien and Boisgerault, Nicolas | 10.1016/j.omton.2024.200887 EA OCT 2024 | VACCINIA VIRUS;DENDRITIC CELLS;EXOSOMES;DELIVERY |
| Malignant Melanoma-Derived Exosomes Induce Endothelial Damage and Glial Activation on a Human BBB Chip Model | BIOSENSORS-BASEL; 2022 | Wang, P and Wu, YS and Chen, WW and Zhang, M and Qin, JH and Wang, Peng and Wu, Yunsong and Chen, Wenwen and Zhang, Min and Qin, Jianhua | 10.3390/bios12020089 | BLOOD-BRAIN-BARRIER;ON-A-CHIP;NEUROVASCULAR UNIT;CANCER;METASTASIS;NICHE;CELLS |
| Tumor Suppressor Role of hsa-miR-193a-3p and-5p in Cutaneous Melanoma | INTERNATIONAL JOURNAL OF MOLECULAR SCIENCES; 2020 | Polini, B and Carpi, S and Doccini, S and Citi, V and Martelli, A and Feola, S and Santorelli, FM and Cerullo, V and Romanini, A and Nieri, P and Polini, Beatrice and Carpi, Sara and Doccini, Stefano and Citi, Valentina and Martelli, Alma and Feola, Sara and Santorelli, Filippo Maria and Cerullo, Vincenzo and Romanini, Antonella and Nieri, Paola | 10.3390/ijms21176183 | FACTOR RECEPTOR SUPERFAMILY;PI3K/AKT PATHWAY;DOWN-REGULATION;ERBB RECEPTORS;LUNG-CANCER;IN-VIVO;MIR-193A-3P;EXPRESSION;NECROSIS;TROY |
| Low-metastatic melanoma cells acquire enhanced metastatic capability via exosomal transfer of miR-199a-1-5p from highly metastatic melanoma cells | CELL DEATH DISCOVERY; 2022 | Zhao, QT and Chen, H and Li, XS and Zeng, B and Sun, ZW and Liu, DD and Chen, YT and Zhang, YH and Xing, HR and Wang, JY and Zhao, Qiting and Chen, Hao and Li, Xiaoshuang and Zeng, Bin and Sun, Zhiwei and Liu, Doudou and Chen, Yuting and Zhang, Yuhan and Xing, H. Rosie and Wang, Jianyu | 10.1038/s41420-022-00993-8 | TUMOR HETEROGENEITY;EXTRAVASATION;PHENOTYPE |
| Silencing Exosomal circ102927 Inhibits Foot Melanoma Metastasis via Regulating Invasiveness, Epithelial-Mesenchymal Transition and Apoptosis | CANCER MANAGEMENT AND RESEARCH; 2024 | Wan, HY and Zhong, L and Xia, T and Zhang, DD and Wan, Huiying and Zhong, Ling and Xia, Tian and Zhang, Dingding | 10.2147/CMAR.S460315 | CIRCULAR RNA;PROLIFERATION;PROGRESSION;BIOMARKER;INVASION;BIOLOGY;CELLS |
| Melanotransferrin is efficiently sorted on the surface of exosomes secreted by melanoma cells | MELANOMA RESEARCH; 2021 | Bonhoure, A and Henry, L and Morille, M and Aissaoui, N and Bellot, G and Stoebner, PE and Vidal, M and Bonhoure, Anne and Henry, Laurent and Morille, Marie and Aissaoui, Nesrine and Bellot, Gaetan and Stoebner, Pierre-Emmanuel and Vidal, Michel | 10.1097/CMR.0000000000000741 | HUMAN-MALIGNANT MELANOMA;RELIABLE TUMOR-MARKER;TRANSFERRIN RECEPTOR;PHOSPHOLIPASE-C;20S PROTEASOME;IN-VITRO;PLASMA;P97;PROTEIN;EXPRESSION |
| Membrane microvesicles as mediators for melanoma-fibroblasts communication: Roles of the VCAM-1/VLA-4 axis and the ERK1/2 signal pathway | CANCER LETTERS; 2015 | Zhao, XP and Wang, M and Song, Y and Song, K and Yan, TL and Wang, L and Liu, K and Shang, ZJ and Zhao, Xiao-Ping and Wang, Meng and Song, Yong and Song, Kai and Yan, Ting-lin and Wang, Lin and Liu, Ke and Shang, Zheng-jun | 10.1016/j.canlet.2015.01.032 | CELL-ADHESION MOLECULE-1;CANCER;EXOSOMES;SURVIVAL;MIGRATION;PROMOTES;LUNG;MICROENVIRONMENT;GLYCOLYSIS;DEPENDENCE |
| Melanoma-Derived Exosomal miR-125b-5p Educates Tumor Associated Macrophages (TAMs) by Targeting Lysosomal Acid Lipase A (LIPA) | CANCERS; 2020 | Gerloff, D and LÃ¼tzkendorf, J and Moritz, RKC and Wersig, T and MÃ¤der, K and MÃ¼ller, LP and SunderkÃ¶tter, C and Gerloff, Dennis and Luetzkendorf, Jana and Moritz, Rose K. C. and Wersig, Tom and Maeder, Karsten and Mueller, Lutz P. and Sunderkoetter, Cord | 10.3390/cancers12020464 | SUPPRESSOR-CELLS;DOWN-REGULATION;MTOR PATHWAY;EXPRESSION;MICRORNAS;ACTIVATION;CANCER;MIRNA;INFLAMMATION;INVOLVEMENT |
| WNT5A induces release of exosomes containing pro-angiogenic and immunosuppressive factors from malignant melanoma cells | MOLECULAR CANCER; 2014 | EkstrÃ¶m, EJ and Bergenfelz, C and von BÃ¼low, V and Serifler, F and Carlemalm, E and JÃ¶nsson, G and Andersson, T and Leandersson, K and Ekstrom, Elin J. and Bergenfelz, Caroline and von Bulow, Verena and Serifler, Filiz and Carlemalm, Eric and Jonsson, Goran and Andersson, Tommy and Leandersson, Karin | 10.1186/1476-4598-13-88 | PLASMA-MEMBRANE;EPITHELIAL-CELLS;TUMOR EXOSOMES;SUPPRESSOR-CELLS;CHROMAFFIN CELLS;EXOCYTOSIS;CDC42;EXPRESSION;SECRETION;PATHWAYS |
| Melanoma stem cells promote metastasis via exosomal miR-1268a inactivation of autophagy | BIOLOGICAL RESEARCH; 2022 | Li, XS and Liu, DD and Chen, H and Zeng, B and Zhao, QT and Zhang, YH and Chen, YT and Wang, JY and Xing, HR and Li, Xiaoshuang and Liu, Doudou and Chen, Hao and Zeng, Bin and Zhao, Qiting and Zhang, Yuhan and Chen, Yuting and Wang, Jianyu and Xing, H. Rosie | 10.1186/s40659-022-00397-z | CANCER;EXTRAVASATION;PHENOTYPE;DORMANCY;BIOLOGY |
| Interferon-Alpha Decreases Cancer Stem Cell Properties and Modulates Exosomes in Malignant Melanoma | CANCERS; 2023 | Garcia-Ortega, MB and Aparicio, E and GriÃ±Ã¡n-LisÃ³n, C and JimÃ©nez, G and LÃ³pez-Ruiz, E and Palacios, JL and Ruiz-AlcalÃ¡, G and Alba, C and MartÃ­nez, A and Boulaiz, H and PerÃ¡n, M and Hackenberg, M and BraganÃ§a, J and Calado, SM and Marchal, JA and GarcÃ­a, MA and Garcia-Ortega, Maria Belen and Aparicio, Ernesto and Grinan-Lison, Carmen and Jimenez, Gema and Lopez-Ruiz, Elena and Palacios, Jose Luis and Ruiz-Alcala, Gloria and Alba, Cristina and Martinez, Antonio and Boulaiz, Houria and Peran, Macarena and Hackenberg, Michael and Braganca, Jose and Calado, Sofia M. and Marchal, Juan A. and Garcia, Maria Angel | 10.3390/cancers15143666 | SIDE POPULATION;GUIDELINE;TARGET |
| Detecting early-stage malignant melanoma using a calcium switch-enriched exosome subpopulation containing tumor markers as a sample | BIOSENSORS & BIOELECTRONICS; 2022 | Choi, DY and Park, JN and Paek, SH and Choi, SC and Paek, SH and Choi, Da-Yeon and Park, Ji-Na and Paek, Sung-Ho and Choi, Seung-Cheol and Paek, Se-Hwan | 10.1016/j.bios.2021.113828 EA NOV 2021 | VESICLE |
| Tumor-derived microvesicles modulate the establishment of metastatic melanoma in a phosphatidylserine-dependent manner | CANCER LETTERS; 2009 | Lima, LG and Chammas, R and Monteiro, RQ and Moreira, MEC and Barcinski, MA and Lima, Luize G. and Chammas, Roger and Monteiro, Robson Q. and Moreira, Maria Elisabete C. and Barcinski, Marcello A. | 10.1016/j.canlet.2009.03.041 | GROWTH-FACTOR-BETA;MEMBRANE PHOSPHOLIPID ASYMMETRY;PLASMA-MEMBRANE;RELEASED MICROVESICLES;MESSENGER-RNA;T-LYMPHOCYTES;TGF-BETA;CELLS;CANCER;VESICLES |
| <i>LADON</i>, a Natural Antisense Transcript of <i>NODAL</i>, Promotes Tumour Progression and Metastasis in Melanoma | NON-CODING RNA; 2023 | Dutriaux, A and Diazzi, S and Bresesti, C and Hardouin, S and Deshayes, F and Collignon, J and Flagiello, D and Dutriaux, Annie and Diazzi, Serena and Bresesti, Chiara and Hardouin, Sylvie and Deshayes, Frederique and Collignon, Jerome and Flagiello, Domenico | 10.3390/ncrna9060071 | LONG NONCODING RNAS;SIGNALING PATHWAYS;CELL-MIGRATION;CANCER;GENE;EXPRESSION;ACTIVATION;SUPPRESSOR;HYPOTHESIS;PLASTICITY |
| Metabolomic profile of cancer stem cell-derived exosomes from patients with malignant melanoma | MOLECULAR ONCOLOGY; 2021 | Palacios-Ferrer, JL and GarcÃ­a-Ortega, MB and Gallardo-GÃ³mez, M and GarcÃ­a, MA and DÃ­az, C and Boulaiz, H and Valdivia, J and Jurado, JM and Almazan-Fernandez, FM and Arias-Santiago, S and Amezcua, V and Peinado, H and Vicente, F and del Palacio, JP and Marchal, JA and Palacios-Ferrer, Jose Luis and Garcia-Ortega, Maria Belen and Gallardo-Gomez, Maria and Garcia, Maria Angel and Diaz, Caridad and Boulaiz, Houria and Valdivia, Javier and Jurado, Jose Miguel and Almazan-Fernandez, Francisco M. and Arias-Santiago, Salvador and Amezcua, Victor and Peinado, Hector and Vicente, Francisca and Perez del Palacio, Jose and Marchal, Juan A. | 10.1002/1878-0261.12823 EA NOV 2020 | HEPATOCELLULAR-CARCINOMA;THERAPEUTIC TARGET;TUMOR;BIOMARKERS;PLASMA;MICROVESICLES;REVEALS;DISEASE |
| Beta3-Tubulin Is Critical for Microtubule Dynamics, Cell Cycle Regulation, and Spontaneous Release of Microvesicles in Human Malignant Melanoma Cells (A375) | INTERNATIONAL JOURNAL OF MOLECULAR SCIENCES; 2020 | Altonsy, MO and Ganguly, A and Amrein, M and Surmanowicz, P and Li, SS and Lauzon, GJ and Mydlarski, PR and Altonsy, Mohammed O. and Ganguly, Anutosh and Amrein, Matthias and Surmanowicz, Philip and Li, Shu Shun and Lauzon, Gilles J. and Mydlarski, P. Regine | 10.3390/ijms21051656 | ENDOTHELIAL-DERIVED MICROPARTICLES;III BETA-TUBULIN;EXPRESSION;CONVEYORS;APOPTOSIS;INSIGHTS;ISOTYPES |
| Study of Circulating MicroRNA-125b Levels in Serum Exosomes in Advanced Melanoma | ARCHIVES OF PATHOLOGY & LABORATORY MEDICINE; 2014 | Alegre, E and Sanmamed, MF and Rodriguez, C and Carranza, O and MartÃ­n-Algarra, S and GonzÃ¡lez, A and Alegre, Estibaliz and Sanmamed, Miguel F. and Rodriguez, Carmen and Carranza, Omar and Martin-Algarra, Salvador and Gonzalez, Alvaro | 10.5858/arpa.2013-0134-OA | EXPRESSION PROFILES;MALIGNANT-MELANOMA;PLASMA;CELLS;MIRNAS;CANCER;PROGRESSION;BIOMARKERS;DIAGNOSIS;MIR-125B |
| Serum exosomal microRNAs as potent circulating biomarkers for melanoma | MELANOMA RESEARCH; 2018 | Li, TD and Long, SP and Gu, ML and Guo, J and Liu, Y and Zhang, WW and Deng, AM and Li Tengda and Long Shuping and Gu Mingli and Guo Jie and Liu Yun and Zhang Weiwei and Deng Anmei | 10.1097/CMR.0000000000000450 | CUTANEOUS MELANOMA;CANCER;EXPRESSION;PROGRESSION;CELLS |
| High Levels of Exosomes Expressing CD63 and Caveolin-1 in Plasma of Melanoma Patients | PLOS ONE; 2009 | Logozzi, M and De Milito, A and Lugini, L and Borghi, M and CalabrÃ², L and Spada, M and Perdicchio, M and Marino, ML and Federici, C and Iessi, E and Brambilla, D and Venturi, G and Lozupone, F and Santinami, M and Huber, V and Maio, M and Rivoltini, L and Fais, S and Logozzi, Mariantonia and De Milito, Angelo and Lugini, Luana and Borghi, Martina and Calabro, Luana and Spada, Massimo and Perdicchio, Maurizio and Marino, Maria Lucia and Federici, Cristina and Iessi, Elisabetta and Brambilla, Daria and Venturi, Giulietta and Lozupone, Francesco and Santinami, Mario and Huber, Veronica and Maio, Michele and Rivoltini, Licia and Fais, Stefano | 10.1371/journal.pone.0005219 | |
| Metformin Increases Sensitivity of Melanoma Cells to Cisplatin by Blocking Exosomal-Mediated miR-34a Secretion | JOURNAL OF ONCOLOGY; 2021 | Ge, L and Wu, YG and Wan, M and You, Y and Zhai, ZF and Song, ZQ and Ge, Lan and Wu, Yaguang and Wan, Ming and You, Yi and Zhai, Zhifang and Song, Zhiqiang | 10.1155/2021/5525231 | CANCER;THERAPY;P53;ACTIVATION;EXPRESSION;COMPONENT |
| Exosomal miR-211-5p regulates glucose metabolism, pyroptosis, and immune microenvironment of melanoma through GNA15 | PHARMACOLOGICAL RESEARCH; 2023 | Zeng, B and Chen, YT and Chen, H and Zhao, QT and Sun, ZW and Liu, DD and Li, XS and Zhang, YH and Wang, JY and Xing, HR and Zeng, Bin and Chen, Yuting and Chen, Hao and Zhao, Qiting and Sun, Zhiwei and Liu, Doudou and Li, Xiaoshuang and Zhang, Yuhan and Wang, Jianyu and Xing, H. Rosie | 10.1016/j.phrs.2023.106660 EA JAN 2023 | |
| Exosomal miR-19a derived from melanoma cell promotes the vemurafenib resistance of malignant melanoma through directly targeting LRIG1 to reactivate AKT and MAPK pathway | PATHOLOGY RESEARCH AND PRACTICE; 2024 | Luan, WK and Lu, X and Peng, HY and Shen, XL and Rao, M and Ruan, HR and Luan, Wenkang and Lu, Xu and Peng, Huiyong and Shen, Xuanlin and Rao, Min and Ruan, Hongru | 10.1016/j.prp.2024.155410 EA JUL 2024 | BRAF INHIBITOR RESISTANCE;GLUCOSE-METABOLISM;DOWN-REGULATION;CANCER;EXPRESSION;MICROENVIRONMENT;SIGNATURES;DIAGNOSIS;SURVIVAL;UPDATE |
| Exosomes produced by melanoma cells significantly influence the biological properties of normal and cancer-associated fibroblasts | HISTOCHEMISTRY AND CELL BIOLOGY; 2022 | StrnadovÃ¡, K and PfeiferovÃ¡, L and Prikryl, P and DvorÃ¡nkovÃ¡, B and VlcÃ¡k, E and FrydlovÃ¡, J and Vokurka, M and Novotny, J and SÃ¡chovÃ¡, J and HradilovÃ¡, M and BrÃ¡bek, J and SmigovÃ¡, J and RÃ¶sel, D and Smetana, K and KolÃ¡r, M and Lacina, L and Strnadova, Karolina and Pfeiferova, Lucie and Prikryl, Petr and Dvorankova, Barbora and Vlcak, Erik and Frydlova, Jana and Vokurka, Martin and Novotny, Jiri and Sachova, Jana and Hradilova, Miluse and Brabek, Jan and Smigova, Jana and Rosel, Daniel and Smetana, Karel, Jr. and Kolar, Michal and Lacina, Lukas | 10.1007/s00418-021-02052-2 EA NOV 2021 | GROWTH;INVASION |
| Melanoma cell-derived exosomes promote epithelial-mesenchymal transition in primary melanocytes through paracrine/autocrine signaling in the tumor microenvironment | CANCER LETTERS; 2016 | Xiao, DY and Barry, S and Kmetz, D and Egger, M and Pan, JM and Rai, SN and Qu, JF and McMasters, KM and Hao, HY and Xiao, Deyi and Barry, Samantha and Kmetz, Daniel and Egger, Michael and Pan, Jianmin and Rai, Shesh N. and Qu, Jifu and McMasters, Kelly M. and Hao, Hongying | 10.1016/j.canlet.2016.03.050 | LUNG-CANCER CELLS;STEM-CELLS;MIR-200 FAMILY;OVARIAN-CANCER;E-CADHERIN;EMT;EXPRESSION;INVASION;MICROVESICLES;ANGIOGENESIS |
| Circulating melanoma exosomes as diagnostic and prognosis biomarkers | CLINICA CHIMICA ACTA; 2016 | Alegre, E and Zubiri, L and Perez-Gracia, JL and GonzÃ¡lez-Cao, M and Soria, L and MartÃ­n-Algarra, S and GonzÃ¡lez, A and Alegre, Estibaliz and Zubiri, Leyre and Luis Perez-Gracia, Jose and Gonzalez-Cao, Maria and Soria, Lourdes and Martin-Algarra, Salvador and Gonzalez, Alvaro | 10.1016/j.cca.2015.12.031 | TUMOR-RELEASED MICROVESICLES;LACTATE-DEHYDROGENASE;COLORECTAL-CANCER;MEMBRANE-VESICLES;S100B PROTEIN;CELLS;MIA;MARKERS;CAVEOLIN-1;ASCITES |
| Increased Microtubule Growth Triggered by Microvesicle-mediated Paracrine Signaling is Required for Melanoma Cancer Cell Invasion | CANCER RESEARCH COMMUNICATIONS; 2022 | Pudelko, K and Wieland, A and Hennecke, M and Raeschle, M and Bastians, H and Pudelko, Karoline and Wieland, Angela and Hennecke, Magdalena and Raeschle, Markus and Bastians, Holger | 10.1158/2767-9764.CRC-22-0010 | EXTRA CENTROSOMES;OXIDATIVE STRESS;MIGRATION;DYNAMICS;HETERODIMERIZATION;PROTRUSION;PROTEASOME |
| The plasma exosomal miR-1180-3p serves as a novel potential diagnostic marker for cutaneous melanoma | CANCER CELL INTERNATIONAL; 2021 | Guo, YY and Zhang, X and Wang, LH and Li, M and Shen, MX and Zhou, Z and Zhu, SS and Li, KK and Fang, ZQ and Yan, B and Zhao, S and Su, J and Chen, X and Peng, C and Guo, Yeye and Zhang, Xu and Wang, Linconghua and Li, Min and Shen, Minxue and Zhou, Zhe and Zhu, Susi and Li, Keke and Fang, Zhiqin and Yan, Bei and Zhao, Shuang and Su, Juan and Chen, Xiang and Peng, Cong | 10.1186/s12935-021-02164-8 | ECONOMIC BURDEN;MICRORNAS;BIOMARKERS;CARCINOMA;DISEASE;CANCER;STAGE |
| Melanoma exosomes educate bone marrow progenitor cells toward a pro-metastatic phenotype through MET | NATURE MEDICINE; 2012 | Peinado, H and Aleckovic, M and Lavotshkin, S and Matei, I and Costa-Silva, B and Moreno-Bueno, G and Hergueta-Redondo, M and Williams, C and GarcÃ­a-Santos, G and Ghajar, CM and Nitadori-Hoshino, A and Hoffman, C and Badal, K and Garcia, BA and Callahan, MK and Yuan, JD and Martins, VR and Skog, J and Kaplan, RN and Brady, MS and Wolchok, JD and Chapman, PB and Kang, YB and Bromberg, J and Lyden, D and Peinado, Hector and Aleckovic, Masa and Lavotshkin, Simon and Matei, Irina and Costa-Silva, Bruno and Moreno-Bueno, Gema and Hergueta-Redondo, Marta and Williams, Caitlin and Garcia-Santos, Guillermo and Ghajar, Cyrus M. and Nitadori-Hoshino, Ayuko and Hoffman, Caitlin and Badal, Karen and Garcia, Benjamin A. and Callahan, Margaret K. and Yuan, Jianda and Martins, Vilma R. and Skog, Johan and Kaplan, Rosandra N. and Brady, Mary S. and Wolchok, Jedd D. and Chapman, Paul B. and Kang, Yibin and Bromberg, Jacqueline and Lyden, David | 10.1038/nm.2753 | PREMETASTATIC NICHE;RELEASED EXOSOMES;SIGNALING PATHWAY;C-MET;CANCER;MICROVESICLES;GROWTH;RECRUITMENT;EXPRESSION;MECHANISM |
| Detection of Exosomal miRNAs in the Plasma of Melanoma Patients | JOURNAL OF CLINICAL MEDICINE; 2015 | Pfeffer, SR and Grossmann, KF and Cassidy, PB and Yang, CH and Fan, MY and Kopelovich, L and Leachman, SA and Pfeffer, LM and Pfeffer, Susan R. and Grossmann, Kenneth F. and Cassidy, Pamela B. and Yang, Chuan He and Fan, Meiyun and Kopelovich, Levy and Leachman, Sancy A. and Pfeffer, Lawrence M. | 10.3390/jcm4121957 | MICRORNA EXPRESSION;CIRCULATING MICRORNAS;CDKN2A MUTATIONS;CANCER;PATHWAY;MIR-21;GENE;INDUCTION;SIGNATURE;MIR-17-92 |
| Acidic microenvironment plays a key role in human melanoma progression through a sustained exosome mediated transfer of clinically relevant metastatic molecules | JOURNAL OF EXPERIMENTAL & CLINICAL CANCER RESEARCH; 2018 | Boussadia, Z and Lamberti, J and Mattei, F and Pizzi, E and Puglisi, R and Zanetti, C and Pasquini, L and Fratini, F and Fantozzi, L and Felicetti, F and Fecchi, K and Raggi, C and Sanchez, M and D'Atri, S and CarÃ¨, A and Sargiacomo, M and Parolini, I and Boussadia, Zaira and Lamberti, Jessica and Mattei, Fabrizio and Pizzi, Elisabetta and Puglisi, Rossella and Zanetti, Cristiana and Pasquini, Luca and Fratini, Federica and Fantozzi, Luca and Felicetti, Federica and Fecchi, Katia and Raggi, Carla and Sanchez, Massimo and D'Atri, Stefania and Care, Alessandra and Sargiacomo, Massimo and Parolini, Isabella | 10.1186/s13046-018-0915-z | MESENCHYMAL TRANSITION;EXTRACELLULAR PH;TUMOR PH;CELLS;CANCER;MICROVESICLES;PROMOTE;FLOW;SUBPOPULATIONS;EXPRESSION |
| Extracellular microvesicle microRNAs as predictive biomarkers for targeted therapy in metastastic cutaneous malignant melanoma | PLOS ONE; 2018 | Svedman, FC and Lohcharoenkal, W and Bottai, M and Brage, SE and Sonkoly, E and Hansson, J and Pivarcsi, A and Eriksson, H and Svedman, Fernanda Costa and Lohcharoenkal, Warangkana and Bottai, Matteo and Brage, Suzanne Egyhazi and Sonkoly, Eniko and Hansson, Johan and Pivarcsi, Andor and Eriksson, Hanna | 10.1371/journal.pone.0206942 | IMPROVED SURVIVAL;DOWN-REGULATION;BRAF;IDENTIFICATION;DABRAFENIB;RESISTANCE;CELLS;MULTICENTER;VEMURAFENIB;INHIBITION |
| uPAR-expressing melanoma exosomes promote angiogenesis by VE-Cadherin, EGFR and uPAR overexpression and rise of ERK1,2 signaling in endothelial cells | CELLULAR AND MOLECULAR LIFE SCIENCES; 2021 | Biagioni, A and Laurenzana, A and Menicacci, B and Peppicelli, S and Andreucci, E and Bianchini, F and Guasti, D and Paoli, P and SerratÃ¬, S and Mocali, A and Calorini, L and Del Rosso, M and Fibbi, G and ChillÃ , A and Margheri, F and Biagioni, Alessio and Laurenzana, Anna and Menicacci, Beatrice and Peppicelli, Silvia and Andreucci, Elena and Bianchini, Francesca and Guasti, Daniele and Paoli, Paolo and Serrati, Simona and Mocali, Alessandra and Calorini, Lido and Del Rosso, Mario and Fibbi, Gabriella and Chilla, Anastasia and Margheri, Francesca | 10.1007/s00018-020-03707-4 EA NOV 2020 | UROKINASE PLASMINOGEN-ACTIVATOR;GROWTH-FACTOR RECEPTOR;LIPID RAFTS;CANCER;BIOLOGY;SERUM;INHIBITION;BIOMARKERS;PLASMA;SYSTEM |
| Constitutive expression and costimulatory function of LIGHT/TNFSF14 on human melanoma cells and melanoma-derived microvesicles | CANCER RESEARCH; 2005 | Mortarini, R and Scarito, A and Nonaka, D and Zanon, M and Bersani, I and Montaldi, E and Pennacchioli, E and Patuzzo, R and Santinami, M and Anichini, A and Mortarini, R and Scarito, A and Nonaka, D and Zanon, M and Bersani, I and Montaldi, E and Pennacchioli, E and Patuzzo, R and Santinami, M and Anichini, A | 10.1158/0008-5472.CAN-04-3239 | LYMPHOTOXIN-BETA-RECEPTOR;HERPESVIRUS ENTRY MEDIATOR;TUMOR-NECROSIS-FACTOR;ACTIVATED T-CELLS;METASTATIC MELANOMA;LYMPHOID-TISSUES;DENDRITIC CELLS;STROMAL CELLS;LIGHT;APOPTOSIS |
| Exosomes released by metabotropic glutamate receptor 1 (GRM1) expressing melanoma cells increase cell migration and invasiveness | ONCOTARGET; 2018 | Isola, AL and Eddy, K and Zembrzuski, K and Goydos, JS and Chen, SZ and Isola, Allison L. and Eddy, Kevinn and Zembrzuski, Krzysztof and Goydos, James S. and Chen, Suzie | 10.18632/oncotarget.23455 | GLUTAMATE-RECEPTOR-1;MICROVESICLES;ACTIVATION;MESSENGER;VESICLES;GROWTH;RAS |
| Malignant transformation in melanocytes is associated with increased production of procoagulant microvesicles | THROMBOSIS AND HAEMOSTASIS; 2011 | Lima, LG and Oliveira, AS and Campos, LC and Bonamino, M and Chammas, R and Werneck, CC and Vicente, CP and Barcinski, MA and Petersen, LC and Monteiro, RQ and Lima, Luize G. and Oliveira, Andreia S. and Campos, Luiza C. and Bonamino, Martin and Chammas, Roger and Werneck, Claudio C. and Vicente, Cristina P. and Barcinski, Marcello A. and Petersen, Lars C. and Monteiro, Robson Q. | 10.1160/TH11-03-0143 | TISSUE FACTOR EXPRESSION;BINDS ANIONIC PHOSPHOLIPIDS;MURINE MELANOMA-CELLS;TUMOR BLOOD-VESSELS;MONOCLONAL-ANTIBODY;CELLULAR MICROPARTICLES;COAGULATION ACTIVATION;VENOUS THROMBOEMBOLISM;MOLECULAR-MECHANISMS;METASTATIC MELANOMA |
| Electroporation-derived melanoma extracellular particles activate fibroblasts | BIOCHIMICA ET BIOPHYSICA ACTA-GENERAL SUBJECTS; 2024 | Choromanska, A and Szwedowicz, U and Szewczyk, A and Daczewska, M and Saczko, J and Kruszakin, R and Pawlik, KJ and Baczynska, D and Kulbacka, J and Choromanska, Anna and Szwedowicz, Urszula and Szewczyk, Anna and Daczewska, Malgorzata and Saczko, Jolanta and Kruszakin, Roksana and Pawlik, Krzysztof J. and Baczynska, Dagmara and Kulbacka, Julita | 10.1016/j.bbagen.2024.130723 EA OCT 2024 | FOCAL-ADHESION KINASE;CANCER-CELLS;ELECTROCHEMOTHERAPY;EXOSOMES;PROLIFERATION;METASTASES;MIGRATION;INVASION |
| Microenvironmental pH Is a Key Factor for Exosome Traffic in Tumor Cells | JOURNAL OF BIOLOGICAL CHEMISTRY; 2009 | Parolini, I and Federici, C and Raggi, C and Lugini, L and Palleschi, S and De Milito, A and Coscia, C and Iessi, E and Logozzi, M and Molinari, A and Colone, M and Tatti, M and Sargiacomo, M and Fais, S and Parolini, Isabella and Federici, Cristina and Raggi, Carla and Lugini, Luana and Palleschi, Simonetta and De Milito, Angelo and Coscia, Carolina and Iessi, Elisabetta and Logozzi, Mariantonia and Molinari, Agnese and Colone, Marisa and Tatti, Massimo and Sargiacomo, Massimo and Fais, Stefano | 10.1074/jbc.M109.041152 | LAURDAN FLUORESCENCE;MEMBRANE-FUSION;PROTEOMIC ANALYSIS;INDUCE APOPTOSIS;PLASMA-MEMBRANE;MELANOMA-CELLS;T-LYMPHOCYTES;VESICLES;MICROVESICLES;CANCER |
| Self-calibrated magnetic aptamer sensor with dual Lanthanide-assisted Time-resolved luminescence for high-sensitive detection of melanoma exosomal PD-L1 | MATERIALS & DESIGN; 2023 | Zhu, NH and Wang, XK and Zhang, YJ and Mao, YC and Yu, Y and Yi, QY and Wu, Y and Zhu, Nanhang and Wang, Xuekang and Zhang, Yujia and Mao, Yanchao and Yu, Yue and Yi, Qiangying and Wu, Yao | 10.1016/j.matdes.2023.111714 EA FEB 2023 | CANCER;LIGHT |
| Biochemical and biological characterization of exosomes containing prominin-1/CD133 | MOLECULAR CANCER; 2013 | Rappa, G and Mercapide, J and Anzanello, F and Pope, RM and Lorico, A and Rappa, Germana and Mercapide, Javier and Anzanello, Fabio and Pope, Robert M. and Lorico, Aurelio | 10.1186/1476-4598-12-62 | HUMAN HEMATOPOIETIC STEM;DECOY SEARCH STRATEGY;PEPTIDE IDENTIFICATIONS;STATISTICAL-MODEL;MEMBRANE-LIPIDS;CANCER CELLS;TUMOR-GROWTH;PROTEINS;MICRORNA;CD133 |
| Detection of micro-plasma-induced exosomes secretion in a fibroblast-melanoma co-culture model | ANALYTICA CHIMICA ACTA; 2023 | Lee, H and Liao, JD and Wong, TW and Wu, CW and Huang, BY and Wu, SC and Shao, PL and Wei, YH and Cheng, MH and Lee, Han and Liao, Jiunn-Der and Wong, Tak-Wah and Wu, Che-Wei and Huang, Bo-Yao and Wu, Shun-Cheng and Shao, Pei -Lin and Wei, Yu-Han and Cheng, Ming-Hsien | 10.1016/j.aca.2023.341910 EA OCT 2023 | PROTEINS;VESICLES;CANCER;CELLS |
| Identifying mRNA, MicroRNA and Protein Profiles of Melanoma Exosomes | PLOS ONE; 2012 | Xiao, DY and Ohlendorf, J and Chen, YL and Taylor, DD and Rai, SN and Waigel, S and Zacharias, W and Hao, HY and McMasters, KM and Xiao, Deyi and Ohlendorf, Joanna and Chen, Yinlu and Taylor, Douglas D. and Rai, Shesh N. and Waigel, Sabine and Zacharias, Wolfgang and Hao, Hongying and McMasters, Kelly M. | 10.1371/journal.pone.0046874 | TUMOR-GROWTH;ADAPTER PROTEIN;IN-VITRO;CELLS;METASTASIS;HYALURONAN;ACTIVATION;MDA-9/SYNTENIN;MICROVESICLES;ACCUMULATION |
| Melanoma cell-secreted exosomal miR-1555p induce proangiogenic switch of cancer-associated fibroblasts via SOCS1/JAK2/STAT3 signaling pathway | JOURNAL OF EXPERIMENTAL & CLINICAL CANCER RESEARCH; 2018 | Zhou, XC and Yan, TL and Huang, CM and Xu, Z and Wang, L and Jiang, EH and Wang, H and Chen, Y and Liu, K and Shao, Z and Shang, ZJ and Zhou, Xiaocheng and Yan, Tinglin and Huang, Chunming and Xu, Zhi and Wang, Lin and Jiang, Erhui and Wang, Hui and Chen, Yang and Liu, Ke and Shao, Zhe and Shang, Zhengjun | 10.1186/s13046-018-0911-3 | MALIGNANT-MELANOMA;TUMOR ANGIOGENESIS;MICRORNAS;COMMUNICATION;SUPPRESSOR;CARCINOMA;INVASION |
| Exosome Release and Low pH Belong to a Framework of Resistance of Human Melanoma Cells to Cisplatin | PLOS ONE; 2014 | Federici, C and Petrucci, F and Caimi, S and Cesolini, A and Logozzi, M and Borghi, M and D'Ilio, S and Lugini, L and Violante, N and Azzarito, T and Majorani, C and Brambilla, D and Fais, S and Federici, Cristina and Petrucci, Francesco and Caimi, Stefano and Cesolini, Albino and Logozzi, Mariantonia and Borghi, Martina and D'Ilio, Sonia and Lugini, Luana and Violante, Nicola and Azzarito, Tommaso and Majorani, Costanza and Brambilla, Daria and Fais, Stefano | 10.1371/journal.pone.0088193 | PROTON PUMP INHIBITORS;INTRACELLULAR PH;H+-ATPASES;CHEMOTHERAPY;MECHANISMS;EXPRESSION;PLASMA;TRAFFICKING;APOPTOSIS;VESICLES |
| A Lipidomic Approach to Identify Potential Biomarkers in Exosomes From Melanoma Cells With Different Metastatic Potential | FRONTIERS IN PHYSIOLOGY; 2021 | Lobasso, S and Tanzarella, P and Mannavola, F and Tucci, M and Silvestris, F and Felici, C and Ingrosso, C and Corcelli, A and Lopalco, P and Lobasso, Simona and Tanzarella, Paola and Mannavola, Francesco and Tucci, Marco and Silvestris, Francesco and Felici, Claudia and Ingrosso, Chiara and Corcelli, Angela and Lopalco, Patrizia | 10.3389/fphys.2021.748895 | CANCER-CELLS;BIS(MONOACYLGLYCERO)PHOSPHATE;MEMBRANE;METABOLISM;MECHANISM;AUTOTAXIN;RECEPTOR;BIOLOGY |
| MicroRNAs in Tumor Exosomes Drive Immune Escape in Melanoma | CANCER IMMUNOLOGY RESEARCH; 2020 | Vignard, V and LabbÃ©, M and Marec, N and AndrÃ©-GrÃ©goire, G and Jouand, N and Fonteneau, JF and LabarriÃ¨re, N and Fradin, D and Vignard, Virginie and Labbe, Maureen and Marec, Nadege and Andre-Gregoire, Gwennan and Jouand, Nicolas and Fonteneau, Jean-Francois and Labarriere, Nathalie and Fradin, Delphine | 10.1158/2326-6066.CIR-19-0522 | CD45;ACTIVATION;MODULATE;RNA;EXPRESSION;ANTIGEN;MIRNAS;CELLS |
| Melanoma exosomes enable tumor tolerance in lymph nodes | MEDICAL HYPOTHESES; 2016 | Hood, JL and Hood, Joshua L. | 10.1016/j.mehy.2016.02.018 | PREMETASTATIC NICHE;CELLS;CANCER;MICROVESICLES;METASTASIS;ENDOTHELIUM;MODULATION;EXPRESSION;MECHANISM;ROLES |
| Melanoma exosome induction of endothelial cell GM-CSF in pre-metastatic lymph nodes may result in different M1 and M2 macrophage mediated angiogenic processes | MEDICAL HYPOTHESES; 2016 | Hood, JL and Hood, Joshua L. | 10.1016/j.mehy.2016.07.009 | TUMOR;CANCER;GROWTH;FACTOR-2-ALPHA;MICROVESICLES;EXPRESSION;RELEASE;ROLES |
| Exosomes Released by Melanoma Cells Prepare Sentinel Lymph Nodes for Tumor Metastasis | CANCER RESEARCH; 2011 | Hood, JL and Roman, SS and Wickline, SA and Hood, Joshua L. and Roman, Susana San and Wickline, Samuel A. | 10.1158/0008-5472.CAN-10-4455 | MEMBRANE-VESICLES;VEGF-B;GROWTH;MICROVESICLES;ANGIOGENESIS;EXPRESSION;BIOMARKERS;PROMOTE;ALPHA;RNA |
| MicroRNA-300: A Transcellular Mediator in Exosome Regulates Melanoma Progression | FRONTIERS IN ONCOLOGY; 2019 | Chen, L and Karisma, VW and Liu, HW and Zhong, L and Chen, Long and Karisma, Vega Windy and Liu, Huawen and Zhong, Li | 10.3389/fonc.2019.01005 | INDUCED OXIDATIVE STRESS;INDUCED DNA-DAMAGE;SKIN;RADIATION;GENE;UVB;BIOINFORMATICS;APOPTOSIS;VESICLES;PATHWAYS |
| Exosomal miR-106b-5p derived from melanoma cell promotes primary melanocytes epithelial-mesenchymal transition through targeting EphA4 | JOURNAL OF EXPERIMENTAL & CLINICAL CANCER RESEARCH; 2021 | Luan, WK and Ding, YT and Xi, HL and Ruan, HR and Lu, F and Ma, SJ and Wang, JL and Luan, Wenkang and Ding, Yuting and Xi, Haolan and Ruan, Hongru and Lu, Feng and Ma, Shaojun and Wang, Jinlong | 10.1186/s13046-021-01906-w | GLUCOSE-METABOLISM;MEDIATED TRANSFER;STEM-CELLS;EXPRESSION;GROWTH;METASTASIS;ACTIVATION;MUTATIONS;MICRORNAS;MECHANISM |
| Exosome-delivered circRPS5 inhibits the progression of melanoma via regulating the miR-151a/NPTX1 axis | PLOS ONE; 2023 | Zhu, HJ and Zhang, P and Shi, J and Kou, DQ and Bai, XP and Zhu, Haijun and Zhang, Pan and Shi, Jia and Kou, Deqiang and Bai, Xinping | 10.1371/journal.pone.0287347 | CIRCRNAS;PROMOTES |
| Cluster of Differentiation 46 Is the Major Receptor in Human Blood-Brain Barrier Endothelial Cells for Uptake of Exosomes Derived from Brain-Metastatic Melanoma Cells (SK-Mel-28) | MOLECULAR PHARMACEUTICS; 2019 | Kuroda, H and Tachikawa, M and Yagi, Y and Umetsu, M and Nurdin, A and Miyauchi, E and Watanabe, M and Uchida, Y and Terasaki, T and Kuroda, Hiroki and Tachikawa, Masanori and Yagi, Yuta and Umetsu, Mina and Nurdin, Armania and Miyauchi, Eisuke and Watanabe, Michitoshi and Uchida, Yasuo and Terasaki, Tetsuya | 10.1021/acs.molpharmaceut.8b00985 | ABSORPTIVE-MEDIATED ENDOCYTOSIS;HEPARAN-SULFATE PROTEOGLYCANS;TARGETED PROTEOMICS;MASS-SPECTROMETRY;EPITHELIAL-CELLS;EXPRESSION;CD46;INTEGRINS;PROTEINS;INTERNALIZATION |
| Melanoma exosomes promote mixed M1 and M2 macrophage polarization | CYTOKINE; 2018 | Bardi, GT and Smith, MA and Hood, JL and Bardi, Gina T. and Smith, Mary Ann and Hood, Joshua L. | 10.1016/j.cyto.2018.02.002 | TUMOR-ASSOCIATED MACROPHAGES;NECROSIS-FACTOR-ALPHA;SUPPRESSOR-CELLS;NITRIC-OXIDE;LYMPH-NODES;RECRUITMENT;GROWTH;NANOPARTICLE;INFLAMMATION;METASTASIS |
| TIM-3 shuttled by MV3 cells-secreted exosomes inhibits CD4<SUP>+</SUP> T cell immune function and induces macrophage M2 polarization to promote the growth and metastasis of melanoma cells | TRANSLATIONAL ONCOLOGY; 2022 | Li, XH and Liu, Y and Yang, L and Jiang, YN and Qian, QH and Li, Xinghui and Liu, Yu and Yang, Li and Jiang, Yannan and Qian, Qihong | 10.1016/j.tranon.2021.101334 EA JAN 2022 | |
| Plasma Exosome-Derived SENP1 May Be a Potential Prognostic Predictor for Melanoma | FRONTIERS IN ONCOLOGY; 2021 | Hu, HJ and Ling, B and Shi, YH and Wu, HH and Zhu, BY and Meng, YL and Zhang, GM and Hu, Hejuan and Ling, Bai and Shi, Yuhan and Wu, Haohao and Zhu, Bingying and Meng, Yiling and Zhang, Guo-Ming | 10.3389/fonc.2021.685009 | CANCER |
| Melanoma cell-derived exosomes in plasma of melanoma patients suppress functions of immune effector cells | SCIENTIFIC REPORTS; 2020 | Sharma, P and Diergaarde, B and Ferrone, S and Kirkwood, JM and Whiteside, TL and Sharma, Priyanka and Diergaarde, Brenda and Ferrone, Soldano and Kirkwood, John M. and Whiteside, Theresa L. | 10.1038/s41598-019-56542-4 | T-CELL;INDUCE APOPTOSIS;NECK-CANCER;TUMOR;MICROVESICLES;VESICLES;BIOLOGY;HEAD;SURFACE;SERA |
| Plasma exosome-derived connexin43 as a promising biomarker for melanoma patients | BMC CANCER; 2023 | Shen, Y and Li, M and Liao, L and Gao, SY and Wang, YZ and Shen, Yue and Li, Ming and Liao, Li and Gao, Suyue and Wang, Yongzhen | 10.1186/s12885-023-10705-9 | CLINICAL-SIGNIFICANCE;E-CADHERIN;EXPRESSION;CANCER;EPIDEMIOLOGY;DIAGNOSIS;BIOLOGY;MARKER;CX43 |
| Blockage of transferred exosome-shuttled miR-494 inhibits melanoma growth and metastasis | JOURNAL OF CELLULAR PHYSIOLOGY; 2019 | Li, JJ and Chen, J and Wang, SH and Li, P and Zheng, CL and Zhou, X and Tao, YG and Chen, X and Sun, LC and Wang, AJ and Cao, K and Tang, SJ and Zhou, JD and Li, Jingjing and Chen, Jia and Wang, Shaohua and Li, Ping and Zheng, Changli and Zhou, Xiao and Tao, Yongguang and Chen, Xiang and Sun, Lichun and Wang, Aijun and Cao, Ke and Tang, Shijie and Zhou, Jianda | 10.1002/jcp.28234 | CANCER EXOSOMES;TUMOR;APOPTOSIS;INITIATE;CELLS |
| Suppression of exosomal hsa_circ_0001005 eliminates the Vemurafenib resistance of melanoma | JOURNAL OF CANCER RESEARCH AND CLINICAL ONCOLOGY; 2023 | Wang, XC and Cheng, Q and Wang, Xicheng and Cheng, Qiong | 10.1007/s00432-022-04434-y EA JAN 2023 | CELLS;RNA;PROGRESSION;IDENTIFICATION;EXPRESSION;PREDICTION;DATABASE |
| Analysis of the miRNA Profiles of Melanoma Exosomes Derived Under Normoxic and Hypoxic Culture Conditions | ANTICANCER RESEARCH; 2017 | Wozniak, M and Peczek, L and Czernek, L and DÃ¼chler, M and Wozniak, Michal and Peczek, Lukasz and Czernek, Liliana and Duchler, Markus | 10.21873/anticanres.12138 | CELL LUNG-CANCER;MESSENGER-RNAS;COLORECTAL-CANCER;STEM-CELLS;MICRORNAS;ANGIOGENESIS;METASTASIS;EXPRESSION;PHENOTYPE;MIGRATION |
| Abrogation of RAB27A expression transiently affects melanoma cell proliferation | PIGMENT CELL & MELANOMA RESEARCH; 2020 | Guo, D and Beaumont, KA and Sharp, DM and Lui, GYL and Weninger, W and Haass, NK and Tikoo, S and Guo, Dajiang and Beaumont, Kimberley A. and Sharp, Danae M. and Lui, Goldie Y. L. and Weninger, Wolfgang and Haass, Nikolas K. and Tikoo, Shweta | 10.1111/pcmr.12903 EA JUN 2020 | INVASION |
| Dual-Isolation and Profiling of Circulating Tumor Cells and Cancer Exosomes from Blood Samples with Melanoma Using Immunoaffinity-Based Microfluidic Interfaces | ADVANCED SCIENCE; 2020 | Kang, YT and Hadlock, T and Lo, TW and Purcell, E and Mutukuri, A and Fouladdel, S and Raguera, MD and Fairbairn, H and Murlidhar, V and Durham, A and McLean, SA and Nagrath, S and Kang, Yoon-Tae and Hadlock, Thomas and Lo, Ting-Wen and Purcell, Emma and Mutukuri, Anusha and Fouladdel, Shamileh and Raguera, Monica De Silva and Fairbairn, Heather and Murlidhar, Vasudha and Durham, Alison and McLean, Scott A. and Nagrath, Sunitha | 10.1002/advs.202001581 EA AUG 2020 | VESICLES;SURVIVAL;PROGRESSION;DEVICE;PCR |
| Melanoma-derived exosomes induce reprogramming fibroblasts into cancer-associated fibroblasts via Gm26809 delivery | CELL CYCLE; 2019 | Hu, TR and Hu, JC and Hu, Tairan and Hu, Jiacai | 10.1080/15384101.2019.1669380 EA SEP 2019 | NONCODING RNAS;CARCINOMA |
| Inhibition of xCT suppresses the efficacy of anti-PD-1/L1 melanoma treatment through exosomal PD-L1-induced macrophage M2 polarization | MOLECULAR THERAPY; 2021 | Liu, N and Zhang, JL and Yin, MZ and Liu, H and Zhang, X and Li, JD and Yan, B and Guo, YY and Zhou, JD and Tao, J and Hu, S and Chen, X and Peng, C and Liu, Nian and Zhang, JiangLin and Yin, Mingzhu and Liu, Hong and Zhang, Xu and Li, Jiaoduan and Yan, Bei and Guo, Yeye and Zhou, Jianda and Tao, Juan and Hu, Shuo and Chen, Xiang and Peng, Cong | 10.1016/j.ymthe.2021.03.013 | TUMOR-ASSOCIATED MACROPHAGES;OXIDATIVE STRESS;CANCER;RESISTANCE;PD-L1;SULFASALAZINE;ANTIPORTER;EXPRESSION;PROMOTES;GROWTH |
| RAB27A promotes melanoma cell invasion and metastasis <i>via</i> regulation of pro-invasive exosomes | INTERNATIONAL JOURNAL OF CANCER; 2019 | Guo, DJ and Lui, GYL and Lai, SL and Wilmott, JS and Tikoo, S and Jackett, LA and Quek, C and Brown, DL and Sharp, DM and Kwan, RYQ and Chacon, D and Wong, JH and Beck, D and van Geldermalsen, M and Holst, J and Thompson, JF and Mann, GJ and Scolyer, RA and Stow, JL and Weninger, W and Haass, NK and Beaumont, KA and Guo, Dajiang and Lui, Goldie Y. L. and Lai, Siew Li and Wilmott, James S. and Tikoo, Shweta and Jackett, Louise A. and Quek, Camelia and Brown, Darren L. and Sharp, Danae M. and Kwan, Rain Y. Q. and Chacon, Diego and Wong, Jason H. and Beck, Dominik and van Geldermalsen, Michelle and Holst, Jeff and Thompson, John F. and Mann, Graham J. and Scolyer, Richard A. and Stow, Jennifer L. and Weninger, Wolfgang and Haass, Nikolas K. and Beaumont, Kimberley A. | 10.1002/ijc.32064 | DIFFERENTIAL EXPRESSION;TUMOR MICROENVIRONMENT;CANCER;GROWTH;GENE;PROGRESSION;BIOGENESIS;MECHANISMS;SECRETION;MIGRATION |
| Melanoma-Derived Exosomes Endow Fibroblasts with an Invasive Potential via miR-21 Target Signaling Pathway | CANCER MANAGEMENT AND RESEARCH; 2020 | Wang, CMY and Wang, YT and Chang, XL and Ba, XY and Hu, N and Liu, Q and Fang, LQ and Wang, ZB and Wang, Chenmeiyi and Wang, Yiting and Chang, Xiulin and Ba, Xiaoyun and Hu, Na and Liu, Qing and Fang, Liaoqiong and Wang, Zhibiao | 10.2147/CMAR.S273718 | MATRIX METALLOPROTEINASES;TUMOR;MICROVESICLES;EXPRESSION;BIOGENESIS;MICRORNAS;MECHANISM;GROWTH;CELLS |
| Synergistic inhibition of NUDT21 by secretory S100A11 and exosomal miR-487a-5p promotes melanoma oligo- to poly-metastatic progression | MOLECULAR ONCOLOGY; 2023 | Zeng, B and Chen, YT and Chen, H and Zhao, QT and Sun, ZW and Liu, DD and Li, XS and Zhang, YH and Wang, JY and Xing, HR and Zeng, Bin and Chen, Yuting and Chen, Hao and Zhao, Qiting and Sun, Zhiwei and Liu, Doudou and Li, Xiaoshuang and Zhang, Yuhan and Wang, Jianyu and Xing, H. Rosie | 10.1002/1878-0261.13480 EA JUL 2023 | CANCER;GLYCOLYSIS;SUPPRESSES;RESISTANCE;GROWTH |
| Tracking the evolution of circulating exosomal-PD-L1 to monitor melanoma patients | JOURNAL OF EXTRACELLULAR VESICLES; 2020 | Cordonnier, M and Nardin, C and Chanteloup, G and Derangere, V and Algros, MP and Arnould, L and Garrido, C and Aubin, F and Gobbo, J and Cordonnier, Marine and Nardin, Charlee and Chanteloup, Gaetan and Derangere, Valentin and Algros, Marie-Paule and Arnould, Laurent and Garrido, Carmen and Aubin, Francois and Gobbo, Jessica | 10.1080/20013078.2019.1710899 | TUMOR RESPONSE;EXOSOMES;IMMUNOTHERAPY;SUPPRESSION;BIOGENESIS;PLASMA;HEAD |
| T-Lymphocytes Activated by Dendritic Cells Loaded by Tumor-Derived Vesicles Decrease Viability of Melanoma Cells In Vitro | CURRENT ISSUES IN MOLECULAR BIOLOGY; 2023 | Filin, IY and Mayasin, YP and Kharisova, CB and Gorodilova, AV and Chulpanova, DS and Kitaeva, KV and Rizvanov, AA and Solovyeva, VV and Filin, Ivan Yurevich and Mayasin, Yuriy Pavlovich and Kharisova, Chulpan Bulatovna and Gorodilova, Anna Valerevna and Chulpanova, Daria Sergeevna and Kitaeva, Kristina Viktorovna and Rizvanov, Albert Anatolyevich and Solovyeva, Valeria Vladimirovna | 10.3390/cimb45100493 | GM-CSF;HELPER-CELLS;IMMUNOTHERAPY;ANTIGEN;GENERATION;RESPONSES;EXOSOMES;IMMUNITY;TH17;TH2 |
| Exosomal lncRNA Mir100hg derived from cancer stem cells enhance glycolysis and promote metastasis of melanoma through miR-16-5p and miR-23a-3p | EXPERIMENTAL CELL RESEARCH; 2024 | Tan, JY and Tang, Y and Li, BW and Shi, L and Zhang, YH and Chen, YT and Chen, Y and Li, J and Xiang, M and Zhou, YF and Xing, HR and Wang, JY and Tan, Jiyu and Tang, Yao and Li, Bowen and Shi, Lei and Zhang, Yuhan and Chen, Yuting and Chen, Yan and Li, Jie and Xiang, Meng and Zhou, Yufeng and Xing, H. Rosie and Wang, Jianyu | 10.1016/j.yexcr.2024.114319 EA NOV 2024 | NONCODING RNA;TUMOR MICROENVIRONMENT;MESSENGER-RNA;UP-REGULATION;PROGRESSION;PRINCIPLES;EXPRESSION;ROOT |
| An Immunosuppressive Effect of Melanoma-derived Exosomes on NY-ESO-1 Antigen-specific Human CD8<SUP>+</SUP>T Cells is Dependent on IL-10 and Independent of BRAF<SUP>V600E</SUP>Mutation in Melanoma Cell Lines | IMMUNOLOGICAL INVESTIGATIONS; 2020 | Shu, SL and Matsuzaki, J and Want, MY and Conway, A and Benjamin-Davalos, S and Allen, CL and Koroleva, M and Battaglia, S and Odunsi, A and Minderman, H and Ernstoff, MS and Shu, ShinLa and Matsuzaki, Junko and Want, Muzamil Y. and Conway, Alexis and Benjamin-Davalos, Shawna and Allen, Cheryl L. and Koroleva, Marina and Battaglia, Sebastiano and Odunsi, Adekunle and Minderman, Hans and Ernstoff, Marc S. | 10.1080/08820139.2020.1803353 EA AUG 2020 | IFN-GAMMA;T-CELLS;BRAF MUTATIONS;TUMOR;PATHWAY;PD-1 |
| Melanoma-released exosomes directly activate the mitochondrial apoptotic pathway of CD4<SUP>+</SUP> T cells through their microRNA cargo | EXPERIMENTAL CELL RESEARCH; 2018 | Zhou, J and Yang, Y and Wang, WW and Zhang, Y and Chen, ZR and Hao, CL and Zhang, JP and Zhou, Ji and Yang, Yi and Wang, WenWen and Zhang, Yuan and Chen, ZhengRong and Hao, ChuangLi and Zhang, JinPing | 10.1016/j.yexcr.2018.08.030 | TUMOR-DERIVED EXOSOMES;INDUCE APOPTOSIS;IMMUNE-SYSTEM;EXPRESSION;NKG2D;SUPPRESSION;CARCINOMA;MICROVESICLES;CYTOTOXICITY;REJECTION |
| PDL1-positive exosomes suppress antitumor immunity by inducing tumor-specific CD8<SUP>+</SUP> T cell exhaustion during metastasis | CANCER SCIENCE; 2021 | Chen, J and Song, Y and Miao, F and Chen, G and Zhu, YJ and Wu, N and Pang, LW and Chen, ZM and Chen, XF and Chen, Ji and Song, Yang and Miao, Feng and Chen, Gang and Zhu, Yongjun and Wu, Ning and Pang, Liewen and Chen, Zhiming and Chen, Xiaofeng | 10.1111/cas.15033 EA JUL 2021 | EPIGENETIC LANDSCAPE;PD-1;MELANOMA;DIFFERENTIATION;BLOCKADE;SUBSETS;CD4(+);IMMUNOTHERAPY;MICROVESICLES;PERSISTENCE |
| Exosomal CD73 from serum of patients with melanoma suppresses lymphocyte functions and is associated with therapy resistance to anti-PD-1 agents | JOURNAL FOR IMMUNOTHERAPY OF CANCER; 2022 | Turiello, R and Capone, M and Morretta, E and Monti, MC and Madonna, G and Azzaro, R and Del Gaudio, P and Simeone, E and Sorrentino, A and Ascierto, PA and Morello, S and Turiello, Roberta and Capone, Mariaelena and Morretta, Elva and Monti, Maria Chiara and Madonna, Gabriele and Azzaro, Rosa and Del Gaudio, Pasquale and Simeone, Ester and Sorrentino, Antonio and Ascierto, Paolo A. and Morello, Silvana | 10.1136/jitc-2021-004043 | BIOLOGICALLY-ACTIVE EXOSOMES;T-CELLS;ACQUIRED-RESISTANCE;ADENOSINE;PLASMA;PD-L1;PHENOTYPE;BLOCKADE;TUMOR |
| Magnetic resonance imaging of melanoma exosomes in lymph nodes | MAGNETIC RESONANCE IN MEDICINE; 2015 | Hu, LZ and Wickline, SA and Hood, JL and Hu, Lingzhi and Wickline, Samuel A. and Hood, Joshua L. | 10.1002/mrm.25376 | STEM-CELLS;DELIVERY;TOOLS;SIRNA |
| Melanoma cell-derived exosomes alter macrophage and dendritic cell functions <i>in vitro</i> | IMMUNOLOGY LETTERS; 2012 | Marton, A and Vizler, C and Kusz, E and Temesfoi, V and Szathmary, Z and Nagy, K and Szegletes, Z and Varo, G and Siklos, L and Katona, RL and Tubak, V and Howard, OMZ and Duda, E and Minarovits, J and Nagy, K and Buzas, K and Marton, Annamaria and Vizler, Csaba and Kusz, Erzsebet and Temesfoi, Viktoria and Szathmary, Zsuzsa and Nagy, Krisztina and Szegletes, Zsolt and Varo, Gyorgy and Siklos, Laszlo and Katona, Robert L. and Tubak, Vilmos and Howard, O. M. Zack and Duda, Erno and Minarovits, Janos and Nagy, Katalin and Buzas, Krisztina | 10.1016/j.imlet.2012.07.006 | NF-KAPPA-B;PROGRESSION;INHIBITOR;TUMORS |
| Maximizing exosome colloidal stability following electroporation | ANALYTICAL BIOCHEMISTRY; 2014 | Hood, JL and Scott, MJ and Wickline, SA and Hood, Joshua L. and Scott, Michael J. and Wickline, Samuel A. | 10.1016/j.ab.2013.12.001 | TUMOR-DERIVED EXOSOMES;STEM-CELLS;MEMBRANE-VESICLES;PROTEIN;ELECTROFUSION;TREHALOSE;NANOPARTICLES;MECHANISM;DELIVERY |
| Comparison of Exosomes Derived from Non- and Gamma-Irradiated Melanoma Cancer Cells as a Potential Antigenic and Immunogenic Source for Dendritic Cell-Based Immunotherapeutic Vaccine | VACCINES; 2020 | Kim, WS and Choi, D and Park, JM and Song, HY and Seo, HS and Lee, DE and Byun, EB and Kim, Woo Sik and Choi, DaeSeong and Park, Ji Min and Song, Ha-Yeon and Seo, Ho Seong and Lee, Dong-Eun and Byun, Eui-Baek | 10.3390/vaccines8040699 | MATURATION;RESPONSES;DECREASE;ELICIT |
| Paracrine induction of endothelium by tumor exosomes | LABORATORY INVESTIGATION; 2009 | Hood, JL and Pan, H and Lanza, GM and Wickline, SA and Hood, Joshua L. and Pan, Hua and Lanza, Gregory M. and Wickline, Samuel A. and C-TRAIN | 10.1038/labinvest.2009.94 | PROMOTE ANGIOGENESIS;INHIBIT ANGIOGENESIS;PROTEOMIC ANALYSIS;MEMBRANE-VESICLES;T-CELLS;MICROPARTICLES;MICROVESICLES;CANCER;GROWTH;MODEL |
| Tumor-released microvesicles as vehicles of immunosuppression | CANCER RESEARCH; 2007 | Valenti, R and Huber, V and Iero, M and Filipazzi, P and Parmiani, G and Rivoltini, L and Valenti, Roberta and Huber, Veronica and Iero, Manuela and Filipazzi, Paola and Parmiani, Giorgio and Rivoltini, Licia | 10.1158/0008-5472.CAN-07-0520 | MEMBRANE-VESICLES;CYTOTOXIC DRUGS;T-LYMPHOCYTES;MYELOID CELLS;EXOSOMES;CANCER;APOPTOSIS;LOCALIZATION;SUPPRESSION;MECHANISMS |
| Redox-signaling transmitted <i>in trans</i> to neighboring cells by melanoma-derived TNF-containing exosomes | FREE RADICAL BIOLOGY AND MEDICINE; 2007 | SÃ¶derberg, A and Barral, AM and SÃ¶derstrÃ¶m, M and Sander, B and RosÃ©n, A and Soderberg, Anita and Barral, Ana Maria and Soderstrom, Mats and Sander, Birgitta and Rosen, Anders | 10.1016/j.freeradbiomed.2007.03.026 | NECROSIS-FACTOR-ALPHA;THIOREDOXIN REDUCTASE;HYDROGEN-PEROXIDE;FULL-LENGTH;EXPRESSION;ACTIVATION;ACCUMULATION;CYTOTOXICITY;GENERATION;MECHANISM |
| Activated T Cell Exosomes Promote Tumor Invasion via Fas Signaling Pathway | JOURNAL OF IMMUNOLOGY; 2012 | Cai, ZJ and Yang, F and Yu, L and Yu, Z and Jiang, LL and Wang, QQ and Yang, YS and Wang, L and Cao, XT and Wang, JL and Cai, Zhijian and Yang, Fei and Yu, Lei and Yu, Zhou and Jiang, Lingling and Wang, Qingqing and Yang, Yunshan and Wang, Lie and Cao, Xuetao and Wang, Jianli | 10.4049/jimmunol.1103466 | RECEPTOR-INDUCED APOPTOSIS;NF-KAPPA-B;MEDIATED APOPTOSIS;C-FLIP;CASPASE-8 ACTIVATION;EFFICIENT INDUCTION;DEATH;CANCER;CD95;LIGAND |
| Replication study: Melanoma exosomes educate bone marrow progenitor cells toward a pro-metastatic phenotype through MET | ELIFE; 2018 | Kim, J and Afshari, A and Sengupta, R and Sebastiano, V and Gupta, A and Kim, YH and Kim, Jeewon and Afshari, Amirali and Sengupta, Ranjita and Sebastiano, Vittorio and Gupta, Archana and Kim, Young H. and Reproducibility Project Canc Biol | 10.7554/eLife.39944 | NICHE FORMATION;TUMOR-GROWTH;BATCH |
| miRNA profiling of B16F10 melanoma cell exosomes reveals melanin synthesis-related genes | HELIYON; 2024 | Jeon, G and Hwang, AR and Park, DY and Kim, JH and Kim, YH and Cho, BK and Min, JH and Jeon, Gyeongchan and Hwang, Ae Rim and Park, Dae-Young and Kim, Ji-Hun and Kim, Yang-Hoon and Cho, Byung-Kwan and Min, Jiho | 10.1016/j.heliyon.2024.e30474 EA MAY 2024 | EXPRESSION;MICROPHTHALMIA;CLASSIFICATION;MICRORNA;MITF |
| The heat shock protein HSP70 promotes mouse NK cell activity against tumors that express inducible NKG2D ligands | JOURNAL OF IMMUNOLOGY; 2007 | Elsner, L and Muppala, V and Gehrmann, M and Lozano, J and Malzahn, D and BickebÃ¶ller, H and Brunner, E and Zientkowska, M and Herrmann, T and Walter, L and Alves, F and Multhoff, G and Dressel, R and Elsner, Leslie and Muppala, Vijayakumar and Gehrmann, Mathias and Lozano, Jingky and Malzahn, Doerthe and Bickeboeller, Heike and Brunner, Edgar and Zientkowska, Marta and Herrmann, Thomas and Walter, Lutz and Alves, Franke and Multhoff, Gabriele and Dressel, Ralf | 10.4049/jimmunol.179.8.5523 | MHC-CLASS-I;SELECTIVE DEPLETION;CYTOLYTIC ACTIVITY;CHAIN-A;HEAT-SHOCK-PROTEIN-70;RECEPTOR;CANCER;GENES;IMMUNOGENICITY;DEATH |
| The effects of tumor-derived exosomes enriched with miRNA-211a on B16F10 cells | WSPOLCZESNA ONKOLOGIA-CONTEMPORARY ONCOLOGY; 2024 | Atashzar, MR and Ataollahi, MR and Asad, AG and Doroudgar, P and Amani, D and Atashzar, Mohammad Reza and Ataollahi, Mohammad Reza and Asad, Ali Ghanbari and Doroudgar, Parisa and Amani, Davar | 10.5114/wo.2024.142364 | MEDIATED DELIVERY;GENETIC EXCHANGE;IN-VITRO;CANCER;THERAPY;PROLIFERATION;METASTASIS;INHIBITOR;MICRORNAS;MECHANISM |
| Secretion of Active Membrane Type 1 Matrix Metalloproteinase (MMP-14) Into Extracellular Space in Microvesicular Exosomes | JOURNAL OF CELLULAR BIOCHEMISTRY; 2008 | Hakulinen, J and Sankkila, L and Sugiyama, N and Lehti, K and Keski-Oja, J and Hakulinen, Juha and Sankkila, Lotta and Sugiyama, Nami and Lehti, Kaisa and Keski-Oja, Jorma | 10.1002/jcb.21923 | TUMOR-DERIVED EXOSOMES;HUMAN ENDOTHELIAL-CELLS;1-MATRIX METALLOPROTEINASE;REJECTION ANTIGENS;COFACTOR PROTEIN;TISSUE INHIBITOR;MT1-MMP;ACTIVATION;SURFACE;VESICLES |
| Novel phosphatidylserine-binding molecule enhances antitumor T-cell responses by targeting immunosuppressive exosomes in human tumor microenvironments | JOURNAL FOR IMMUNOTHERAPY OF CANCER; 2021 | Bhatta, M and Shenoy, GN and Loyall, JL and Gray, BD and Bapardekar, M and Conway, A and Minderman, H and Kelleher, RJ and Carreno, BM and Linette, G and Shultz, LD and Odunsi, K and Balu-Iyer, SV and Pak, KY and Bankert, RB and Bhatta, Maulasri and Shenoy, Gautam N. and Loyall, Jenni L. and Gray, Brian D. and Bapardekar, Meghana and Conway, Alexis and Minderman, Hans and Kelleher Jr, Raymond J. and Carreno, Beatriz M. and Linette, Gerald and Shultz, Leonard D. and Odunsi, Kunle and Balu-Iyer, Sathy, V and Pak, Koon Yan and Bankert, Richard B. | 10.1136/jitc-2021-003148 | RECOGNITION;IL-12;XENOGRAFTS;BIOMARKERS;MODEL |
| Melanoma Derived Exosomes Amplify Radiotherapy Induced Abscopal Effect via IRF7/I-IFN Axis in Macrophages | ADVANCED SCIENCE; 2024 | Wang, L and Shen, KJ and Gao, ZX and Ren, M and Wei, CL and Yang, Y and Li, YL and Zhu, Y and Zhang, SM and Ding, YT and Zhang, TY and Li, JR and Zhu, M and Zheng, SL and Yang, YW and Du, SS and Wei, CY and Gu, JY and Wang, Lu and Shen, Kangjie and Gao, Zixu and Ren, Ming and Wei, Chenlu and Yang, Yang and Li, Yinlam and Zhu, Yu and Zhang, Simin and Ding, Yiteng and Zhang, Tianyi and Li, Jianrui and Zhu, Ming and Zheng, Shaoluan and Yang, Yanwen and Du, Shisuo and Wei, Chuanyuan and Gu, Jianying | 10.1002/advs.202304991 EA JAN 2024 | CIRCULAR RNA;RADIATION-THERAPY;CANCER;ENHANCEMENT;CONTRIBUTES |
| Human tumor-released microvesicles promote the differentiation of myeloid cells with transforming growth factor-Î²-mediated suppressive activity on T lymphocytes | CANCER RESEARCH; 2006 | Valenti, R and Huber, V and Filipazzi, P and Pilla, L and Sovena, G and Villa, A and Corbelli, A and Fais, S and Parmiani, G and Rivoltini, L and Valenti, Roberta and Huber, Veronica and Filipazzi, Paola and Pilla, Lorenzo and Sovena, Gloria and Villa, Antonello and Corbelli, Alessandro and Fais, Stefano and Parmiani, Giorgio and Rivoltini, Licia | 10.1158/0008-5472.CAN-06-1819 | PROTEOMIC ANALYSIS;FAS LIGAND;PERIPHERAL-BLOOD;DENDRITIC CELLS;IN-VITRO;EXOSOMES;CANCER;VESICLES;IMMUNOSURVEILLANCE;IDENTIFICATION |
| Introduction of the <i>CIITA</i> gene into tumor cells produces exosomes with enhanced anti-tumor effects | EXPERIMENTAL AND MOLECULAR MEDICINE; 2011 | Lee, YS and Kim, SH and Cho, JA and Kim, CW and Lee, Yeong Shin and Kim, Soo Hyun and Cho, Jung Ah and Kim, Chul Woo | 10.3858/emm.2011.43.5.029 | CYTOTOXIC T-LYMPHOCYTES;CLASS-II TRANSACTIVATOR;MULTIVESICULAR BODY;CANCER;ANTIGEN;REJECTION;ACTIVATION;INDUCTION;VESICLES;VACCINE |
| Visualization and in vivo tracking of the exosomes of murine melanoma B16-BL6 cells in mice after intravenous injection | JOURNAL OF BIOTECHNOLOGY; 2013 | Takahashi, Y and Nishikawa, M and Shinotsuka, H and Matsui, Y and Ohara, S and Imai, T and Takakura, Y and Takahashi, Yuki and Nishikawa, Makiya and Shinotsuka, Haruka and Matsui, Yuriko and Ohara, Saori and Imai, Takafumi and Takakura, Yoshinobu | 10.1016/j.jbiotec.2013.03.013 | QUANTITATIVE-ANALYSIS;DELIVERY;RETICULOCYTES;LUCIFERASES;INHIBITION;MATURATION;MECHANISM;PROTEINS;GROWTH;BRAIN |
| PD-L1 mRNA expression in plasma-derived exosomes is associated with response to anti-PD-1 antibodies in melanoma and NSCLC | BRITISH JOURNAL OF CANCER; 2018 | Del Re, M and Marconcini, R and Pasquini, G and Rofi, E and Vivaldi, C and Bloise, F and Restante, G and Arrigoni, E and Caparello, C and Bianco, MG and Crucitta, S and Petrini, I and Vasile, E and Falcone, A and Danesi, R and Del Re, Marzia and Marconcini, Riccardo and Pasquini, Giulia and Rofi, Eleonora and Vivaldi, Caterina and Bloise, Francesco and Restante, Giuliana and Arrigoni, Elena and Caparello, Chiara and Bianco, Maria Grazia and Crucitta, Stefania and Petrini, Iacopo and Vasile, Enrico and Falcone, Alfredo and Danesi, Romano | 10.1038/bjc.2018.9 | CELL LUNG-CANCER;NIVOLUMAB;BLOCKADE |
| Exosomes derived from B16F0 melanoma cells alter the transcriptome of cytotoxic T cells that impacts mitochondrial respiration | FEBS JOURNAL; 2018 | Bland, CL and Byrne-Hoffman, CN and Fernandez, A and Rellick, SL and Deng, W and Klinke, DJ and Bland, Cassidy L. and Byrne-Hoffman, Christina N. and Fernandez, Audry and Rellick, Stephanie L. and Deng, Wentao and Klinke, David J., II | 10.1111/febs.14396 | SET ENRICHMENT ANALYSIS;TUMOR MICROENVIRONMENT;SUPPRESSOR-CELLS;NOTCH LIGANDS;MYELOID CELLS;PHENOTYPE;RNA;DIFFERENTIATION;IDENTIFICATION;EXPRESSION |
| Identification of the SNARE complex that mediates the fusion of multivesicular bodies with the plasma membrane in exosome secretion | JOURNAL OF EXTRACELLULAR VESICLES; 2023 | Liu, CQ and Liu, DX and Wang, S and Gan, L and Yang, XL and Ma, C and Liu, Chuqi and Liu, Dexiang and Wang, Shen and Gan, Lu and Yang, Xiangliang and Ma, Cong | 10.1002/jev2.12356 | METASTASIS;BIOGENESIS;RELEASE;VAMP7 |
| Tumor cell-expressed SerpinB2 is present on microparticles and inhibits metastasis | CANCER MEDICINE; 2014 | Schroder, WA and Major, LD and Le, TT and Gardner, J and Sweet, MJ and Janciauskiene, S and Suhrbier, A and Schroder, Wayne A. and Major, Lee D. and Le, Thuy T. and Gardner, Joy and Sweet, Matthew J. and Janciauskiene, Sabina and Suhrbier, Andreas | 10.1002/cam4.229 | PLASMINOGEN-ACTIVATOR INHIBITOR-2;HT1080 SARCOMA-CELLS;RETINOBLASTOMA PROTEIN;UROKINASE RECEPTOR;CANCER;MICROVESICLES;MELANOMA;INVASION;SURVIVAL;BINDING |
| Programmed Death Ligand-1 in Melanoma and Extracellular Vesicles Promotes Local and Regional Immune Suppression through M2-like Macrophage Polarization | American Journal of Pathology; 2025 | Huang, L. and Yang, J. and Zhu, J. and Wang, H. and Dong, L. and Guo, Y. and Chen, Y. and Zhang, F. and Xu, D.J. and Ou, L. and Xu, J.R. and Guan, L. and Doan, Q.D. and Fan, A.Y. and Zhong, W. and Ko, J. and Liang, C. and Herlyn, M. and Guo, W. and Xu, X. and Liu, S. | 10.1016/j.ajpath.2024.09.011 | CD163 antigen;CXCL2 chemokine;interleukin 10;interleukin 6;macrophage inflammatory protein 1alpha;monocyte chemotactic protein 1;programmed death 1 ligand 1;transforming growth factor beta1;animal cell;animal experiment;animal model;animal tissue;Article;B16-F10 cell line;blood;CCL2 gene;CCL3 gene;CD163 gene;CD274 gene;CD8+ T lymphocyte;clinical outcome;controlled study;CXCL2 gene;dendritic cell;exosome;gene;gene expression;gene knockout;human;IL10 gene;IL6 gene;immune response;in vivo study;M1 macrophage;M2 macrophage;melanoma;melanoma cell line;metastatic melanoma;mouse;MRC1 gene;nonhuman;PD L1 gene;polarization;TGFB1 gene;tumor draining lymph node;tumor growth;wild type;YUMM1.7 cell line |
| Recycled melanoma-secreted melanosomes regulate tumor-associated macrophage diversification | EMBO Journal; 2024 | Parikh, R. and Parikh, S. and Berzin, D. and Vaknine, H. and Ovadia, S. and Likonen, D. and Greenberger, S. and Scope, A. and Elgavish, S. and Nevo, Y. and Plaschkes, I. and Nizri, E. and Kobiler, O. and Maliah, A. and Zaremba, L. and Mohan, V. and Sagi, I. and Ashery-Padan, R. and Carmi, Y. and Luxenburg, C. and Hoheisel, J.D. and Khaled, M. and Levesque, M.P. and Levy, C. | 10.1038/s44318-024-00103-7 | Angiogenesis;Cell-to-Cell-Transfer;Heterogeneity;Melanosomes;Tumor Associated Macrophages;Animals;Cell Communication;Cell Line, Tumor;Extracellular Vesicles;Fibroblasts;Humans;Keratinocytes;Macrophages;Melanocytes;Melanoma;Melanosomes;Mice;Proto-Oncogene Proteins c-akt;Skin Neoplasms;Tumor-Associated Macrophages;Vascular Endothelial Growth Factor A;interleukin 27;mammalian target of rapamycin;mitogen activated protein kinase 1;toll like receptor 3;transcription factor Sox2;tumor necrosis factor receptor associated factor 6;AKT1 protein, human;protein kinase B;vasculotropin A;aggressiveness;Akt/mTOR signaling;angiogenesis;animal cell;animal experiment;animal tissue;Article;astrocyte;B scan;B16-F10 cell line;carcinogenesis;CD4+ T lymphocyte;CD8+ T lymphocyte;cell infiltration;cell isolation;cell migration;cell transfer;coculture;color Doppler flowmetry;controlled study;epidermis;exosome;fibroblast;flow cytometry;fluorescence analysis;gene;gene expression;gene ontology;GIMAP6 gene;high throughput sequencing;human;human cell;immunocompetent cell;immunofluorescence;immunohistochemistry;keratinocyte;macrophage;mass spectrometry;melanocyte;melanogenesis;melanoma;melanoma cell;melanosome;metastatic melanoma;morphogenesis;mouse;nonhuman;peripheral blood mononuclear cell;phenotype;polarization;principal component analysis;protein degradation;protein protein interaction;proteomics;reverse transcription polymerase chain reaction;reversed phase liquid chromatography;RNA sequence;SAMD9L gene;TAP1 gene;tissue microarray;transcriptomics;transmission electron microscopy;tumor growth;tumor microenvironment;tumor volume;tumor-associated macrophage;ultrasound;upregulation;animal;cell communication;exosome;genetics;immunology;metabolism;pathology;skin tumor;tumor cell line |
| Extracellular vesicles from highly invasive melanoma subpopulations increase the invasive capacity of less invasive melanoma cells through mir-1246-mediated inhibition of CCNG2 | Cell Communication and Signaling; 2024 | Kingreen, T. and Kewitz-Hempel, S. and Rohde, C. and Hause, G. and SunderkÃ¶tter, C. and Gerloff, D. | 10.1186/s12964-024-01820-6 | Extracellular vesicles;Invasion;Melanoma;miRNAs;Cell Line, Tumor;Cell Movement;Extracellular Vesicles;Gene Expression Regulation, Neoplastic;Humans;Melanoma;MicroRNAs;Neoplasm Invasiveness;CD63 antigen;CD81 antigen;CD9 antigen;cyclin G2;luciferase;microRNA;microRNA 1246;sphingomyelin phosphodiesterase;unclassified drug;uvomorulin;vimentin;microRNA;MIRN1246 microRNA, human;adult;Article;cell invasion;cohort analysis;controlled study;down regulation;epithelial mesenchymal transition;exosome;female;fluorescence microscopy;gel permeation chromatography;gene overexpression;high throughput sequencing;human;human cell;immunoblotting;luciferase assay;male;melanoma;metastasis;overall survival;real time polymerase chain reaction;RNA isolation;transmission electron microscopy;tumor spheroid;Western blotting;cell motion;gene expression regulation;genetics;metabolism;pathology;tumor cell line;tumor invasion |
| Impact of Rab27 on Melanoma Cell Invasion and sEV Secretion | International Journal of Molecular Sciences; 2024 | Horodecka, K. and Czernek, L. and PÄ™czek, Å. and Gadzinowski, M. and Klink, M. | 10.3390/ijms252212433 | invasiveness;melanoma cells;Rab27;small extracellular vesicles;Cell Line, Tumor;Cell Movement;Extracellular Vesicles;Humans;Melanoma;Neoplasm Invasiveness;rab GTP-Binding Proteins;rab27 GTP-Binding Proteins;guanosine triphosphatase;Ras related protein Rab 27A;Rab protein;RAB27A protein, human;Rab27B protein, human;Ras related protein Rab 27A;A-375 cell line;Article;cell invasion;cell migration;cellular secretion;exosome;human;human cell;melanoma cell;protein content;RKO cell line;SK-MEL-28 cell line;Western blotting;cell motion;genetics;melanoma;metabolism;pathology;tumor cell line;tumor invasion |
| Characterising the HLA-I immunopeptidome of plasma-derived extracellular vesicles in patients with melanoma | Journal of Extracellular Biology; 2024 | Boyne, C. and Coote, A. and Synowsky, S. and Naden, A. and Shirran, S. and Powis, S.J. | 10.1002/jex2.146 | cancer immunology;extracellular vesicles;HLA-I;immunology;immunopeptidomes;tumour associated antigens |
| Genome-Wide Profiling of Extracellular Vesicles Derived from B16 Melanoma Cells Reflects Dynamic Changes in Mutation Profiles of Melanoma Cells | Journal of Biological Regulators and Homeostatic Agents; 2024 | Chang, X. and Hua, Y. and Wang, L. and Jiang, Y. and Fang, L. and Bai, J. | 10.23812/j.biol.regul.homeost.agents.20243806.423 | DNA;evolution;extracellular vesicles;melanoma;mutation;apolipoprotein B;biological marker;bptf protein;cyclin dependent kinase 4;ddb2 protein;deoxyribonuclease I;dlg4 protein;double stranded DNA;Fanconi anemia group D2 protein;fn1 protein;genomic DNA;gnal protein;gnas protein;map3k3 protein;mucin 5AC;myh10 protein;n methyl dextro aspartic acid receptor 2B;ncor2 protein;Notch3 receptor;nrxn3 protein;pdzk1 protein;phosphatidylinositol 3,4,5 trisphosphate 3 phosphatase;pik3c2b protein;plcg1 protein;ppp1cc protein;protein;protein MDMX;serpinb6b protein;SYBR green;tab2 protein;transcription factor NANOG;unclassified drug;uvomorulin;vasculotropin receptor 2;virus DNA;animal cell;animal experiment;animal tissue;Article;B16-F0 cell line;B16-F1 cell line;B16-F10 cell line;bioinformatics;comparative study;controlled study;DNA content;DNA extraction;exosome;gene expression profiling;gene mutation;gene ontology;genetic profile;genome-wide association study;indel mutation;Melan-a cell line;melanoma B16;metastasis potential;missense mutation;mouse;nonhuman;phylogenetic tree;protein protein interaction;single nucleotide polymorphism;staining;whole genome sequencing |
| Profiling miRNAs in Exosomes for the Development of a Diagnostic Panel for Melanoma Metastasis in Melanocyte and Melanoma Cell Line Models | Voprosy Onkologii; 2024 | Antonova, E.I. and Baldueva, I.A. and Kunitsyna, A.V. and Nekhaeva, T.L. and Achilov, A.B. and Koroleva, A.K. and Firsova, N.V. and Sikharulidze, S.V. | 10.37469/0507-3758-2024-70-4-652-660 | diagnosis;melanocytes in vitro;melanoma;metastasis;miRNA;qRT-PCR;biological marker;microRNA;microRNA 149 3p;microRNA 150 5p;microRNA 155;microRNA 193a 5p;microRNA 21 5p;unclassified drug;Article;cancer growth;cancer staging;carcinogenesis;controlled study;cutaneous melanoma cell line;exosome;gene expression level;human;human cell;in vitro study;melanocyte;metastasis;metastatic melanoma;quantitative analysis;RNA fingerprinting |
| The Influence of Melanoma Extracellular Vesicles on Benign Melanocytes: A Role for PRAME in Modulation of the Tumor Microenvironment | Journal of Investigative Dermatology; 2024 | Liu, X. and Janknecht, R. and Asadbeigi, S.N. and Perry, L. and Naqash, A.-R. and Ding, W.-Q. and McBride, J.D. | 10.1016/j.jid.2024.10.612 | Cancer;Extracellular vesicle;Melanoma;Microenvironment;PRAME |
| TP53 mutations correlate with the non-coding RNA content of small extracellular vesicles in melanoma | Journal of Extracellular Biology; 2023 | LabbÃ©, M. and Menoret, E. and Letourneur, F. and Saint-Pierre, B. and de Beaurepaire, L. and Veziers, J. and Dreno, B. and Denis, M.G. and Blanquart, C. and Boisgerault, N. and Fonteneau, J.-F. and Fradin, D. | 10.1002/jex2.105 | long non-coding RNA;microRNA;Small extracellular vesicle;TP53 mutations |
| Optimization of extracellular vesicle isolation and their separation from lipoproteins by size exclusion chromatography | Journal of Extracellular Biology; 2023 | Benayas, B. and Morales, J. and Egea, C. and ArmisÃ©n, P. and YÃ¡Ã±ez-MÃ³, M. | 10.1002/jex2.100 | biofluids;disease biomarkers;extracellular vesicles;lipoproteins;plasma;purification;size exclusion chromatography |
| Localized Imaging of Programmed Death-Ligand 1 on Individual Tumor-Derived Extracellular Vesicles for Prediction of Immunotherapy Response | ACS Nano; 2023 | Zhang, J. and Guan, M. and Lv, M. and Liu, Y. and Zhang, H. and Zhang, Z. and Zhang, K. | 10.1021/acsnano.3c05799 | individual EVs analysis;localized imaging;PD-L1;prediction of immunotherapy response;primer exchange reaction;Animals;B7-H1 Antigen;Extracellular Vesicles;Humans;Immunotherapy;Melanoma;Mice;MicroRNAs;Biochips;Diagnosis;Fluorescence imaging;Forecasting;Ligands;Mammals;CD274 protein, human;microRNA;programmed death 1 ligand 1;Bulk measurement;Exchange reaction;Extracellular;Individual extracellular vesicle analyse;Localised;Localized imaging;Membrane fusion;Prediction of immunotherapy response;Primer exchange reaction;Programmed death-ligand 1;animal;diagnostic imaging;exosome;human;immunotherapy;melanoma;metabolism;mouse;procedures;Tumors |
| Improved Sensitivity in BRAFV600E Detection in Combined Tissue and Extracellular Vesicle-Based Liquid Biopsy in Melanoma | Journal of Investigative Dermatology; 2023 | GarcÃ­a-Silva, S. and Vico-Alonso, C. and Meyer, L. and Enderle, D. and Sanchez, J.A. and Onteniente, M.D.M. and Noerholm, M. and Skog, J. and RodrÃ­guez-Peralto, J.-L. and Ortiz-Romero, P.-L. and Peinado, H. | 10.1016/j.jid.2023.01.025 | Extracellular Vesicles;Humans;Liquid Biopsy;Melanoma;Mutation;Proto-Oncogene Proteins B-raf;B Raf kinase;B Raf kinase;adult;aged;Article;blood sampling;cancer patient;cancer prognosis;clinical article;clinical feature;cohort analysis;controlled study;cutaneous melanoma;dermatologist;exosome;female;gene mutation;genetic variability;human;human tissue;liquid biopsy;male;middle aged;overall survival;prospective study;sensitivity and specificity;very elderly;genetics;liquid biopsy;melanoma;mutation;pathology |
| MAPK inhibitors dynamically affect melanoma release of immune NKG2D-ligands, as soluble protein and extracellular vesicle-associated | Frontiers in Cell and Developmental Biology; 2023 | LÃ³pez-Borrego, S. and Campos-Silva, C. and SandÃºa, A. and Camino, T. and TÃ©llez-PÃ©rez, L. and Alegre, E. and Beneitez, A. and Jara-Acevedo, R. and Paschen, A. and Pardo, M. and GonzÃ¡lez, Ã. and ValÃ©s-GÃ³mez, M. | 10.3389/fcell.2022.1055288 | extracellular vesicles;immune evasion;immunomodulation;metalloproteases;metastatic melanoma;targeted cancer therapy;B Raf kinase inhibitor;binimetinib;cobimetinib;dabrafenib;melanoma antigen;metalloproteinase;mitogen activated protein kinase inhibitor;natural killer cell receptor NKG2D;trametinib;vemurafenib;adult;aged;Article;cancer patient;cell surface;clinical article;controlled study;enzyme inhibition;exosome;female;human;human cell;human tissue;immune evasion;immune response;immune system;immunocompetence;immunomodulation;in vitro study;male;melanoma cell;metastatic melanoma;molecularly targeted therapy;protein blood level;protein expression |
| In Vitro Interaction of Melanoma-Derived Extracellular Vesicles with Collagen | International Journal of Molecular Sciences; 2023 | Palmulli, R. and Bresteau, E. and Raposo, G. and Montagnac, G. and van Niel, G. | 10.3390/ijms24043703 | collagen;ECM;extracellular vesicles;melanoma;Collagen;Extracellular Matrix;Extracellular Vesicles;Humans;Melanoma;Tumor Microenvironment;apolipoprotein E;CD63 antigen;CD9 antigen;collagen gel;collagen type 1;fibronectin;collagen;A-375 cell line;Article;cancer model;cell migration;collagen fiber;collagen fibril;controlled study;electron microscopy;exosome;extracellular matrix;human;human cell;in vitro study;melanoma;melanoma cell line;MNT-1 cell line;nonhuman;protein interaction;pull-down assay;transmission electron microscopy;tumor cell;wm1716 cell line;metabolism;tumor microenvironment |
| Circulating extracellular vesicles expressing PD1 and PD-L1 predict response and mediate resistance to checkpoint inhibitors immunotherapy in metastatic melanoma | Molecular Cancer; 2022 | SerratÃ¬, S. and Guida, M. and Di Fonte, R. and De Summa, S. and Strippoli, S. and Iacobazzi, R.M. and Quarta, A. and De Risi, I. and Guida, G. and Paradiso, A. and Porcelli, L. and Azzariti, A. | 10.1186/s12943-021-01490-9 | Anti-PD1 treatment;Drug resistance;Extracellular vesicles;Metastatic melanoma;PD-L1;PD1;B7-H1 Antigen;Biomarkers, Tumor;Diagnostic Imaging;Drug Resistance, Neoplasm;Extracellular Vesicles;Female;Humans;Immune Checkpoint Inhibitors;Immunophenotyping;Male;Melanoma;Neoplasm Metastasis;Neoplasm Staging;Programmed Cell Death 1 Receptor;Proportional Hazards Models;Reproducibility of Results;CD63 antigen;CD81 antigen;CD9 antigen;immune checkpoint inhibitor;ipilimumab;nivolumab;pembrolizumab;programmed death 1 ligand 1;programmed death 1 receptor;tumor marker;CD274 protein, human;programmed death 1 ligand 1;programmed death 1 receptor;tumor marker;adult;aged;Article;cancer immunotherapy;CD8+ T lymphocyte;cell isolation;cell migration;circulating extracellular vesicle;clinical outcome;cohort analysis;controlled study;correlational study;cutaneous melanoma;diagnostic test accuracy study;drug efficacy;drug response;exosome;female;human;immunocompetent cell;life expectancy;major clinical study;male;metastatic melanoma;mucosal melanoma;observational study;overall response rate;overall survival;predictive value;progression free survival;protein expression;protein secretion;reliability;survival rate;tumor spheroid;uvea melanoma;validation study;cancer staging;diagnostic imaging;drug resistance;exosome;genetics;immunophenotyping;melanoma;metabolism;metastasis;pharmacology;proportional hazards model;reproducibility |
| Extracellular vesicles microRNA-592 of melanoma stem cells promotes metastasis through activation of MAPK/ERK signaling pathway by targeting PTPN7 in non-stemness melanoma cells | Cell Death Discovery; 2022 | Zhang, Y. and Chen, Y. and Shi, L. and Li, J. and Wan, W. and Li, B. and Liu, D. and Li, X. and Chen, Y. and Xiang, M. and Chen, H. and Zeng, B. and Xing, H.R. and Wang, J. | 10.1038/s41420-022-01221-z | |
| A novel microRNA signature for the detection of melanoma by liquid biopsy | Journal of Translational Medicine; 2022 | Sabato, C. and Noviello, T.M.R. and Covre, A. and Coral, S. and Caruso, F.P. and Besharat, Z.M. and Splendiani, E. and Masuelli, L. and Battistelli, C. and Vacca, A. and Catanzaro, G. and Po, A. and Anichini, A. and Maio, M. and Ceccarelli, M. and Di Giacomo, A.M. and Ferretti, E. | 10.1186/s12967-022-03668-1 | Biomarkers signature;Diagnosis;Extracellular vesicles;Liquid biopsy;Melanoma;microRNAs;Biomarkers, Tumor;Circulating MicroRNA;Gene Expression Profiling;Humans;Liquid Biopsy;Melanoma;MicroRNAs;guadecitabine;ipilimumab;microRNA;OX40 ligand;thrombin;circulating microRNA;microRNA;tumor marker;adult;aged;area under the curve;Article;blood sampling;cancer mortality;centrifugation;chemoluminescence;clinical article;clinical trial;cohort analysis;computer assisted tomography;controlled study;cutaneous melanoma;diagnostic test accuracy study;droplet digital polymerase chain reaction;exosome;female;gene expression;gene ontology;hemolysis;human;human tissue;immunoreactivity;immunosuppressive treatment;liquid biopsy;male;melanoma;metastatic melanoma;nuclear magnetic resonance imaging;particle size;personalized medicine;phase 1 clinical trial;quality control;real time polymerase chain reaction;receiver operating characteristic;reverse transcription polymerase chain reaction;RNA isolation;sensitivity and specificity;transmission electron microscopy;very elderly;Western blotting;gene expression profiling;genetics;liquid biopsy;melanoma |
| Extracellular Vesicles Derived from Metastatic Melanoma Cells Transfer nAChR mRNA, Thus Increasing the Surface Expression of the Receptor and Stimulating the Growth of Normal Keratinocytes | Acta Naturae; 2022 | Bychkov, M.L. and Kirichenko, Ð.V. and Mikhaylova, I.N. and Paramonov, A.S. and Kirpichnikov, Ðœ.P. and Shulepko, Ðœ.Ð. and Lyukmanova, E.N. | 10.32607/actanaturae.11734 | Cancer.;Keratinocytes;Metastatic melanoma;Oncotherapy;Vesicles;Î‘7-nachr |
| Extracellular Vesicles Derived from Acidified Metastatic Melanoma Cells Stimulate Growth, Migration, and Stemness of Normal Keratinocytes | Biomedicines; 2022 | Bychkov, M.L. and Kirichenko, A.V. and Mikhaylova, I.N. and Paramonov, A.S. and Yastremsky, E.V. and Kirpichnikov, M.P. and Shulepko, M.A. and Lyukmanova, E.N. | 10.3390/biomedicines10030660 | Adhesion factors;Cancer;Cytokines;Extracellular vesicles;Melanoma;Metastasis;Migration;MiRNA;MRNA;SNAI |
| HSP90/IKK-rich small extracellular vesicles activate pro-angiogenic melanoma-associated fibroblasts via the NF-ÎºB/CXCL1 axis | Cancer Science; 2022 | Tang, H. and Zhou, X. and Zhao, X. and Luo, X. and Luo, T. and Chen, Y. and Liang, W. and Jiang, E. and Liu, K. and Shao, Z. and Shang, Z. | 10.1111/cas.15271 | angiogenesis;cancer-associated fibroblasts;extracellular vesicles;hypoxia;melanoma;Chemokine CXCL1;Extracellular Vesicles;Fibroblasts;HSP90 Heat-Shock Proteins;Humans;Hypoxia;I-kappa B Kinase;I-kappa B Proteins;Melanoma;NF-kappa B;Proteomics;Tumor Microenvironment;chaperone;CXCL1 chemokine;heat shock protein 90;I kappa B;I kappa B kinase;I kappa B kinase alpha;immunoglobulin enhancer binding protein;CXCL1 chemokine;CXCL1 protein, human;heat shock protein 90;I kappa B;I kappa B kinase;immunoglobulin enhancer binding protein;angiogenesis;Article;cancer associated fibroblast;cell migration;cell proliferation assay;coimmunoprecipitation;controlled study;cytosol;data analysis software;enzyme activity;enzyme linked immunosorbent assay;exosome;fibroblast;gene expression;human;human cell;hypoxia;immunofluorescence;immunohistochemistry;in vitro study;in vivo study;melanoma;NF kB signaling;protein expression;protein phosphorylation;proteomics;RNA sequencing;tubulogenesis;upregulation;fibroblast;hypoxia;metabolism;tumor microenvironment |
| Detection of tumor-derived extracellular vesicles in plasma from patients with solid cancer | BMC Cancer; 2021 | Vitale, S.R. and Helmijr, J.A. and Gerritsen, M. and Coban, H. and van Dessel, L.F. and Beije, N. and van der Vlugt-Daane, M. and Vigneri, P. and Sieuwerts, A.M. and Dits, N. and van Royen, M.E. and Jenster, G. and Sleijfer, S. and Lolkema, M. and Martens, J.W.M. and Jansen, M.P.H.M. | 10.1186/s12885-021-08007-z | cfDNA;dPCR;EV-RNA;Liquid biopsy;Biomarkers, Tumor;Cell Line, Tumor;Circulating Tumor DNA;Cohort Studies;Extracellular Vesicles;Humans;Mutation;Neoplasms;RNA, Messenger;CD9 antigen;cell free DNA;circulating tumor DNA;DNA;glyceraldehyde 3 phosphate dehydrogenase;unclassified drug;circulating tumor DNA;messenger RNA;tumor marker;Article;bile duct carcinoma;blood analysis;BRAF gene;breast cancer;cell isolation;clinical article;colon carcinoma;colorectal carcinoma;controlled study;digital polymerase chain reaction;exosome;gene expression;gene frequency;gene identification;genetic transfection;genetic variability;genotype;human;melanoma;non small cell lung cancer;oncogene K ras;PIK3CA gene;plasma;real time polymerase chain reaction;rectum carcinoma;solid malignant neoplasm;tumor-related gene;vein puncture;blood;cohort analysis;comparative study;exosome;genetics;metabolism;mutation;neoplasm;tumor cell line |
| Melanoma-derived small extracellular vesicles induce lymphangiogenesis and metastasis through an NGFR-dependent mechanism | Nature Cancer; 2021 | GarcÃ­a-Silva, S. and Benito-MartÃ­n, A. and NoguÃ©s, L. and HernÃ¡ndez-Barranco, A. and Mazariegos, M.S. and Santos, V. and Hergueta-Redondo, M. and XimÃ©nez-EmbÃºn, P. and Kataru, R.P. and Lopez, A.A. and Merino, C. and SÃ¡nchez-Redondo, S. and GraÃ±a-Castro, O. and Matei, I. and NicolÃ¡s-Avila, J.Ã. and Torres-Ruiz, R. and RodrÃ­guez-Perales, S. and MartÃ­nez, L. and PÃ©rez-MartÃ­nez, M. and Mata, G. and Szumera-CieÄ‡kiewicz, A. and Kalinowska, I. and Saltari, A. and MartÃ­nez-GÃ³mez, J.M. and Hogan, S.A. and Saragovi, H.U. and Ortega, S. and Garcia-Martin, C. and Boskovic, J. and Levesque, M.P. and Rutkowski, P. and Hidalgo, A. and MuÃ±oz, J. and MegÃ­as, D. and Mehrara, B.J. and Lyden, D. and Peinado, H. | 10.1038/s43018-021-00272-y | Animals;Endothelial Cells;Extracellular Vesicles;Humans;Lymphangiogenesis;Lymphatic Metastasis;Melanoma;Mice;Nerve Tissue Proteins;Receptors, Nerve Growth Factor;Tumor Microenvironment;4',6 diamidino 2 phenylindole;eosin;hematoxylin;immunoglobulin enhancer binding protein;intercellular adhesion molecule 1;mitogen activated protein kinase 1;mitogen activated protein kinase 3;nerve growth factor receptor;neurotrophin receptor;short hairpin RNA;nerve growth factor receptor;nerve protein;NGFR protein, human;animal experiment;animal model;animal tissue;Article;bioluminescence;biopsy;cancer survival;cell adhesion;centrifugation;clustered regularly interspaced short palindromic repeat;cohort analysis;colony formation;confocal microscopy;controlled study;down regulation;electron microscopy;endothelium cell;enzyme activity;exosome;flow cytometry;gene expression;histology;housekeeping gene;human;human cell;image analysis;immune response;immunoblotting;immunofluorescence;immunohistochemistry;in vitro study;lymph node metastasis;lymphangiogenesis;male;melanoma;melanoma cell line;metastasis;mouse;mRNA expression level;nonhuman;pilot study;polyacrylamide gel electrophoresis;preclinical study;protein expression;RNA extraction;RNA sequence;sequence analysis;tumor microenvironment;upregulation;Western blotting;animal;genetics;lymphangiogenesis;metabolism;physiology;tumor microenvironment |
| Proteomic profile of melanoma cell-derived small extracellular vesicles in patientsâ€™ plasma: a potential correlate of melanoma progression | Journal of Extracellular Vesicles; 2021 | Pietrowska, M. and Zebrowska, A. and Gawin, M. and Marczak, L. and Sharma, P. and Mondal, S. and Mika, J. and PolaÅ„ska, J. and Ferrone, S. and Kirkwood, J.M. and Widlak, P. and Whiteside, T.L. | 10.1002/jev2.12063 | high-resolution mass spectrometry (HRMS);melanoma cell-derived exosomes (MTEX);proteomics;small extracellular vesicles (sEV);tumour-derived exosomes (TEX);contactin 1;proteome;Article;cancer growth;cancer prognosis;clinical article;controlled study;disease association;exosome;flow cytometry;gene ontology;gene overexpression;human;human cell;mass spectrometry;melanoma;melanoma cell;protein expression;protein fingerprinting;proteomics;upregulation |
| Detection of BRAF splicing variants in plasma-derived cell-free nucleic acids and extracellular vesicles of melanoma patients failing targeted therapy therapies | Oncotarget; 2020 | Clark, M.E. and Rizos, H. and Pereira, M.R. and McEvoy, A.C. and Marsavela, G. and Calapre, L. and Meehan, K. and Ruhen, O. and Khattak, M.A. and Meniawy, T.M. and Long, G.V. and Carlino, M.S. and Menzies, A.M. and Millward, M. and Ziman, M. and Gray, E.S. | 10.18632/oncotarget.27790 | BRAF splicing;Drug resistance;Extracellular vesicles;Melanoma;Targeted therapy;B Raf kinase;CD63 antigen;CD81 antigen;CD9 antigen;circulating microRNA;circulating tumor DNA;dabrafenib;protein p48;protein p55;trametinib;vemurafenib;Article;cancer growth;controlled study;disease burden;DNA splicing;enzyme linked immunosorbent assay;exosome;follow up;gene amplification;gene expression;gene mutation;genetic variability;genotype;human;human cell;immunofluorescence;immunohistochemistry;immunophenotyping;melanoma;molecularly targeted therapy;positron emission tomography;protein expression;RNA extraction;RNA isolation;transmission electron microscopy;Western blotting |
| Small extracellular vesicles convey the stress-induced adaptive responses of melanoma cells | Scientific Reports; 2019 | Harmati, M. and Gyukity-Sebestyen, E. and Dobra, G. and Janovak, L. and Dekany, I. and Saydam, O. and Hunyadi-Gulyas, E. and Nagy, I. and Farkas, A. and Pankotai, T. and Ujfaludi, Z. and Horvath, P. and Piccinini, F. and Kovacs, M. and Biro, T. and Buzas, K. | 10.1038/s41598-019-51778-6 | Animals;Cell Cycle;Cell Movement;Cell Proliferation;Doxorubicin;Endothelial Cells;Extracellular Matrix;Extracellular Vesicles;Male;Melanoma, Experimental;Mesenchymal Stem Cells;Metal Nanoparticles;Mice, Inbred C57BL;MicroRNAs;Proteome;Silver;Stress, Physiological;Titanium;Tumor Microenvironment;doxorubicin;metal nanoparticle;microRNA;proteome;silver;titanium;titanium dioxide;animal;C57BL mouse;cell cycle;cell motion;cell proliferation;chemistry;cytology;drug effect;endothelium cell;exosome;experimental melanoma;extracellular matrix;genetics;male;mesenchymal stem cell;metabolism;pathology;physiological stress;tumor microenvironment;ultrastructure |
| Identification of key microRNAs of plasma extracellular vesicles and their diagnostic and prognostic significance in melanoma | Open Medicine (Poland); 2020 | Xiong, J. and Xue, Y. and Xia, Y. and Zhao, J. and Wang, Y. | 10.1515/med-2020-0111 | Bioinformatics;Differentially expressed genes;Melanoma;Plasma extracellular vesicles;cyclin dependent kinase 2;microRNA;microRNA 550a;unclassified drug;Article;bioinformatics;cancer prognosis;cancer survival;cdk2 gene;clinical article;controlled study;cutaneous melanoma;diagnostic value;DNA microarray;down regulation;exosome;gene;gene expression regulation;gene ontology;human;overall survival;polr2a gene;priority journal;protein protein interaction;upregulation |
| Melanoma-secreted lysosomes trigger monocyte-derived dendritic cell apoptosis and limit cancer immunotherapy | Cancer Research; 2020 | Santana-Magal, N. and Farhat-Younis, L. and Gutwillig, A. and Gleiberman, A. and Rasoulouniriana, D. and Tal, L. and Netanely, D. and Shamir, R. and Blau, R. and Feinmesser, M. and Zlotnik, O. and Gutman, H. and Linde, I.L. and Reticker-Flynn, N.E. and Rider, P. and Carmi, Y. | 10.1158/0008-5472.CAN-19-2944 | Animals;Apoptosis;CD8-Positive T-Lymphocytes;Dendritic Cells;Drug Resistance, Neoplasm;Humans;Immunotherapy;Lymphocyte Activation;Lymphocytes, Tumor-Infiltrating;Lysosomes;Melanoma;Mice;Mice, Inbred C57BL;caspase 3;caspase 7;CD40 antibody;immunological antineoplastic agent;tumor necrosis factor antibody;unclassified drug;adoptive transfer;animal experiment;animal model;animal tissue;apoptosis;Article;cancer growth;cancer immunotherapy;cancer tissue;CD8+ T lymphocyte;cell isolation;confocal microscopy;controlled study;dendritic cell;drug efficacy;exosome;female;flow cytometry;human;human cell;immune system;in vivo study;Lentivirus infection;luminescence;lysosome;male;melanoma;melanoma cell line;mouse;nonhuman;phagocytosis;prevalence;retrovirus infection;scanning electron microscopy;sentinel lymph node;animal;apoptosis;C57BL mouse;dendritic cell;drug resistance;immunology;immunotherapy;lymphocyte activation;melanoma;pathology;tumor associated leukocyte |
| Detection of inflammation-related melanoma small extracellular vesicle (sEV) mRNA content using primary melanocyte sEVs as a reference | International Journal of Molecular Sciences; 2019 | Bardi, G.T. and Al-Rayan, N. and Richie, J.L. and Yaddanapudi, K. and Hood, J.L. | 10.3390/ijms20051235 | Biomarker;Exosome;Extracellular vesicle;Inflammation;Melanoma;MRNA;Biomarkers, Tumor;Cell Line, Tumor;Chemokines, CXC;Extracellular Vesicles;Humans;Inflammation;Melanocytes;Melanoma;RNA, Messenger;Up-Regulation;CD63 antigen;CXCL1 chemokine;CXCL2 chemokine;cyclooxygenase 2;guanine nucleotide binding protein;guanine nucleotide binding protein 1;HLA antigen;interleukin 8;messenger RNA;microRNA;prostaglandin synthase;STAT 1 protein;STAT protein;unclassified drug;alpha chemokine;messenger RNA;tumor marker;adult;Article;biophysics;controlled study;electrophoretic mobility;enzyme linked immunosorbent assay;exosome;female;gene expression;human;human cell;inflammation;male;melanocyte;melanoma;middle aged;photon correlation spectroscopy;real time polymerase chain reaction;reverse transcription polymerase chain reaction;RNA isolation;sucrose density gradient centrifugation;upregulation;zeta potential;exosome;genetics;inflammation;melanoma;pathology;tumor cell line |
| Melanoma-derived extracellular vesicles instigate proinflammatory signaling in the metastatic microenvironment | International Journal of Cancer; 2019 | Gener Lahav, T. and Adler, O. and Zait, Y. and Shani, O. and Amer, M. and Doron, H. and Abramovitz, L. and Yofe, I. and Cohen, N. and Erez, N. | 10.1002/ijc.32521 | astrocytes;cancer-associated fibroblasts;Exosomes;extracellular vesicles;melanoma;metastasis;metastatic niche;Animals;Astrocytes;Exosomes;Extracellular Vesicles;Fibroblasts;Inflammation;Male;Melanoma;Mice;Mice, Inbred C57BL;NIH 3T3 Cells;Paracrine Communication;Signal Transduction;Stromal Cells;Tumor Microenvironment;animal cell;animal experiment;animal model;animal tissue;Article;astrocyte;brain metastasis;cancer associated fibroblast;cellular secretion;controlled study;exosome;gene expression;inflammation;lung fibroblast;lung metastasis;male;melanoma;melanoma cell;metastasis;mouse;nonhuman;nuclear reprogramming;paracrine signaling;priority journal;stroma cell;tumor microenvironment;wound healing;animal;C57BL mouse;exosome;fibroblast;inflammation;melanoma;NIH 3T3 cell line;pathology;physiology;signal transduction;tumor microenvironment |
| Loss of SR-BI down-regulates MITF and suppresses extracellular vesicle release in human melanoma | International Journal of Molecular Sciences; 2019 | Kinslechner, K. and SchÃ¼tz, B. and Pistek, M. and Rapolter, P. and WeitzenbÃ¶ck, H.P. and Hundsberger, H. and Mikulits, W. and Grillari, J. and RÃ¶hrl, C. and HengstschlÃ¤ger, M. and Stangl, H. and Mikula, M. | 10.3390/ijms20051063 | cMET;Extracellular vesicles;Melanoma metastasis;Pigmentation;SCARB1;Secretory pathway;Cell Line, Tumor;Down-Regulation;Extracellular Vesicles;Gene Expression Regulation, Neoplastic;Humans;Melanoma;Microphthalmia-Associated Transcription Factor;rab GTP-Binding Proteins;Scavenger Receptors, Class B;Synaptosomal-Associated Protein 25;Vesicular Transport Proteins;ESCRT protein;high density lipoprotein receptor;microphthalmia associated transcription factor;scavenger receptor BI;small interfering RNA;vasculotropin;microphthalmia associated transcription factor;MITF protein, human;Rab protein;SCARB1 protein, human;scavenger receptor B;SNAP25 protein, human;synaptosomal associated protein 25;vesicular transport protein;Vps25 protein, human;Article;cholesterol transport;confocal microscopy;down regulation;enzyme linked immunosorbent assay;exosome;gene knockdown;human;human cell;immunocytochemistry;immunofluorescence;melanogenesis;melanoma;melanoma cell line;metastatic melanoma;mRNA expression level;polyacrylamide gel electrophoresis;protein expression;proto oncogene;real time polymerase chain reaction;secretory pathway;signal transduction;skin pigmentation;ultracentrifugation;Western blotting;down regulation;gene expression regulation;genetics;melanoma;metabolism;tumor cell line |
| Human melanoma-derived ectosomes are enriched with specific glycan epitopes | Life Sciences; 2018 | Surman, M. and Hoja-Åukowicz, D. and Szwed, S. and DroÅ¼dÅ¼, A. and StÄ™pieÅ„, E. and PrzybyÅ‚o, M. | 10.1016/j.lfs.2018.06.026 | Extracellular vesicles;Glycosylation;Lectins;Melanoma;N-glycans;Biomarkers;Cell Line, Tumor;Cell Membrane;Cell Movement;Cell-Derived Microparticles;Disease Progression;Epitopes;Flow Cytometry;Gene Expression Regulation, Neoplastic;Glycosylation;Humans;Lectins;Lymphatic Metastasis;Melanoma;Polysaccharides;Skin Neoplasms;actin;alkaline phosphatase;alpha5 integrin;beta1 integrin;cell adhesion molecule;epitope;glycan;glyceraldehyde 3 phosphate dehydrogenase;L1CAM protein;lectin;sirtuin 6;unclassified drug;biological marker;epitope;lectin;polysaccharide;Article;cell organelle;cell size;controlled study;cutaneous melanoma cell line;ectosome;human;human cell;in vitro study;protein binding;protein depletion;protein determination;protein glycosylation;protein processing;protein secretion;transmission electron microscopy;validation process;cell membrane;cell motion;chemistry;disease exacerbation;flow cytometry;gene expression regulation;glycosylation;lymph node metastasis;melanoma;membrane microparticle;metabolism;skin tumor;tumor cell line |
| The HDAC6 Inhibitor Tubacin Induces Release of CD133+ Extracellular Vesicles From Cancer Cells | Journal of Cellular Biochemistry; 2017 | Chao, O.S. and Chang, T.C. and Di Bella, M.A. and Alessandro, R. and Anzanello, F. and Rappa, G. and Goodman, O.B. and Lorico, A. | 10.1002/jcb.26095 | CANCER;CD133;EXOSOMES;EXTRACELLULAR VESICLES;HDAC6;LIPID;TUBACIN;AC133 Antigen;Anilides;Cell Line, Tumor;Cell-Derived Microparticles;Histone Deacetylase 6;Histone Deacetylase Inhibitors;Humans;Hydroxamic Acids;Neoplasm Proteins;Neoplasms;CD133 antigen;glycosphingolipid;histone deacetylase 6;lamin A;lamin C;lysophosphatidylcholine;phosphatidic acid;phosphatidylcholine;phosphatidylethanolamine;phosphatidylglycerol;phosphatidylserine;ricolinostat;sphingomyelin;trichostatin A;tubacin;anilide;CD133 antigen;HDAC6 protein, human;histone deacetylase 6;histone deacetylase inhibitor;hydroxamic acid;PROM1 protein, human;tubacin;tumor protein;antineoplastic activity;Article;Caco-2 cell line;cancer cell;cell aggregation;cell death;cell survival;cell viability;clonogenesis;colorectal carcinoma;controlled study;down regulation;drug effect;enzyme activity;exosome;extracellular space;histone acetylation;human;human cell;in vitro study;intracellular signaling;lipid composition;metastatic melanoma;priority journal;protein expression;antagonists and inhibitors;membrane microparticle;metabolism;neoplasm;tumor cell line |
| Intercellular Resistance to BRAF Inhibition Can Be Mediated by Extracellular Vesicleâ€“Associated PDGFRÎ² | Neoplasia (United States); 2017 | Vella, L.J. and Behren, A. and Coleman, B. and Greening, D.W. and Hill, A.F. and Cebon, J. | 10.1016/j.neo.2017.07.002 | Animals;Cattle;Cell Proliferation;Coculture Techniques;Dose-Response Relationship, Drug;Drug Resistance, Neoplasm;Exosomes;Extracellular Fluid;Extracellular Vesicles;Humans;Indoles;Melanoma;Proto-Oncogene Proteins B-raf;Receptor, Platelet-Derived Growth Factor beta;Sulfonamides;Tumor Cells, Cultured;B Raf kinase;B Raf kinase inhibitor;epidermal growth factor receptor;mitogen activated protein kinase;mitogen activated protein kinase 1;mitogen activated protein kinase 3;n [3 (5 chloro 1h pyrrolo[2,3 b]pyridine 3 carbonyl) 2,4 difluorophenyl]propanesulfonamide;phosphatidylinositol 3 kinase;platelet derived growth factor beta receptor;protein kinase B;B Raf kinase;BRAF protein, human;indole derivative;n [3 (5 chloro 1h pyrrolo[2,3 b]pyridine 3 carbonyl) 2,4 difluorophenyl]propanesulfonamide;PDGFRB protein, human;platelet derived growth factor beta receptor;sulfonamide;Article;cancer resistance;controlled study;drug sensitivity;enzyme inhibition;exosome;human;human cell;melanoma;melanoma cell;metastatic melanoma;phenotype;Pi3K/Akt signaling;priority journal;tumor growth;animal;antagonists and inhibitors;bovine;cell proliferation;coculture;dose response;drug effect;drug resistance;exosome;extracellular fluid;melanoma;metabolism;pathology;physiology;tumor cell culture |
| Role of Phosphatidylserine-Derived Negative Surface Charges in the Recognition and Uptake of Intravenously Injected B16BL6-Derived Exosomes by Macrophages | Journal of Pharmaceutical Sciences; 2017 | Matsumoto, A. and Takahashi, Y. and Nishikawa, M. and Sano, K. and Morishita, M. and Charoenviriyakul, C. and Saji, H. and Takakura, Y. | 10.1016/j.xphs.2016.07.022 | clearance;lipids;nanoparticles;pharmacokinetics;phospholipids;physicochemical;static charge;Animals;Cell Line, Tumor;Exosomes;Injections, Intravenous;Liposomes;Macrophages;Male;Mice;Mice, Inbred BALB C;Phosphatidylserines;Surface Properties;(3 iodobenzoyl)norbiotinamide i 125;liposome;luciferase lactadherin fusion protein;phosphatidylcholine;phosphatidylglycerol;phosphatidylserine;radiopharmaceutical agent;recombinant protein;streptavidin lactadherin fusion protein;unclassified drug;liposome;phosphatidylserine;adipose tissue;animal cell;animal experiment;animal tissue;area under the curve;Article;bladder tissue;blood;bone tissue;brain tissue;controlled study;drug accumulation;drug clearance;drug half life;drug uptake;exosome;heart tissue;in vitro study;in vivo study;intestine tissue;kidney tissue;liposomal delivery;liver tissue;lung parenchyma;male;mean residence time;melanoma cell line;mouse;nonhuman;particle size;peritoneum macrophage;physical chemistry;plasma clearance;quantitative analysis;spleen tissue;stomach tissue;surface charge;tissue distribution;zeta potential;animal;Bagg albino mouse;exosome;intravenous drug administration;macrophage;metabolism;surface property;tumor cell line |
| Proteomic profiling of NCI-60 extracellular vesicles uncovers common protein cargo and cancer type-specific biomarkers | Oncotarget; 2016 | Hurwitz, S.N. and Rider, M.A. and Bundy, J.L. and Liu, X. and Singh, R.K. and Meckes, D.G. | 10.18632/oncotarget.13569 | Biomarkers;Co-inertia;Exosomes;Microvesicles;Proteomics;Biomarkers, Tumor;Cell Line, Tumor;Extracellular Vesicles;Humans;Proteomics;adenosine diphosphate ribosylation factor 4;agrin;alix protein;biological marker;CD147 antigen;CD63 antigen;CD81 antigen;cellubrevin;flotillin 1;gelatinase A;glypican 1;heat shock cognate protein 70;integrin;intercellular adhesion molecule 3;lipocortin 2;premelanosome protein;proteome;Rab protein 10;Rab protein 11B;Rab protein 14;Rab protein 1A;Rab protein 2A;Rab protein 5C;Rab protein 6A;Rab protein 7A;Rab protein 8A;syntenin 1;tenascin XB;tumor susceptibility gene 101 protein;unclassified drug;unindexed drug;tumor marker;Article;breast cancer;cancer diagnosis;cancer prognosis;cell composition;central nervous system tumor;colon cancer;controlled study;exosome;human;human cell;kidney cancer;leukemia;lung cancer;melanoma;national health organization;ovary cancer;protein analysis;protein function;protein localization;proteomics;chemistry;exosome;procedures;proteomics;tumor cell line |
| Quantitative analysis of tissue distribution of the B16BL6-derived exosomes using a streptavidin-lactadherin fusion protein and Iodine-125-Labeled biotin derivative after intravenous injection in mice | Journal of Pharmaceutical Sciences; 2015 | Morishita, M. and Takahashi, Y. and Nishikawa, M. and Sano, K. and Kato, K. and Yamashita, T. and Imai, T. and Saji, H. and Takakura, Y. | 10.1002/jps.24251 | Biomaterials;Clearance;Exosome;Lactadherin;Nanoparticles;Pharmacokinetics;Phospholipids;Quantitative analysis;Radioisotope;Streptavidin;Animals;Biotin;Exosomes;Injections, Intravenous;Iodine Radioisotopes;Male;Melanoma, Experimental;Mice;Mice, Inbred BALB C;Protein Binding;Recombinant Fusion Proteins;Streptavidin;Tissue Distribution;biotin derivative;hybrid protein;iodine 125;lactadherin;luciferase;plasmid vector;streptavidin;biotin;hybrid protein;protein binding;radioactive iodine;streptavidin;animal cell;animal experiment;animal tissue;Article;bladder;circulation;controlled study;exosome;intestine;isotope labeling;liver;lung;male;melanoma cell;mouse;nonhuman;physical chemistry;quantitative analysis;radioactivity;spleen;tissue distribution;zeta potential;animal;Bagg albino mouse;drug effects;exosome;experimental melanoma;intravenous drug administration;metabolism;physiology;tissue distribution |
| Macrophage-dependent clearance of systemically administered B16BL6-derived exosomes from the blood circulation in mice | Journal of Extracellular Vesicles; 2015 | Imai, T. and Takahashi, Y. and Nishikawa, M. and Kato, K. and Morishita, M. and Yamashita, T. and Matsumoto, A. and Charoenviriyakul, C. and Takakura, Y. | 10.3402/jev.v4.26238 | Clearance;Exosome;Gaussia luciferase;Kupffer cell;Lactadherin;Splenic macrophage;Mus;clodronic acid;fluorescent dye;lactadherin;liposome;animal cell;area under the curve;Article;cell function;cell labeling;chemoluminescence;circulation;complement activation;complement classical pathway;controlled study;endothelium cell;exosome;immunoelectron microscopy;immunofluorescence test;Kupffer cell;liver;lung endothelium;macrophage;male;mean residence time;melanoma cell line;mouse;nonhuman;protein binding;spleen;Western blotting |
| Epigenetic transfer of metastatic activity by uptake of highly metastatic B16 melanoma cell-released exosomes | Experimental Oncology; 2006 | Hao, S. and Ye, Z. and Li, F. and Meng, Q. and Qureshi, M. and Yang, J. and Xiang, J. | | Exosome;Lung metastasis;Melanoma;Animals;Antigens, Neoplasm;Cytoplasmic Vesicles;Epigenesis, Genetic;Lung Neoplasms;Melanoma, Experimental;Mice;Mice, Inbred C57BL;Skin Neoplasms;antibody;cell surface marker;fluorescein isothiocyanate;formaldehyde;major histocompatibility antigen class 1;major histocompatibility antigen class 2;paraffin;ribonuclease;tumor antigen;tumor marker;animal cell;animal experiment;animal model;animal tissue;article;controlled study;epigenetics;female;flow cytometry;histopathology;lung metastasis;lung tumor;melanoma B16;membrane vesicle;metastasis;mouse;nonhuman;phenotype;tumor cell line |
| Proteomic analysis of melanoma-derived exosomes by two-dimensional polyacrylamide gel electrophoresis and mass spectrometry | Proteomics; 2005 | Mears, R. and Craven, R.A. and Hanrahan, S. and Totty, N. and Upton, C. and Young, S.L. and Patel, P. and Selby, P.J. and Banks, R.E. | 10.1002/pmic.200400876 | Exosomes;Mel-CAM;Melanoma;Two-dimensional polyacrylamide gel electrophoresis;Antigens, Neoplasm;Blotting, Western;Cell Line, Tumor;Cell Nucleus;Cytochromes c;Electrophoresis, Gel, Two-Dimensional;Electrophoresis, Polyacrylamide Gel;Gene Expression Regulation, Neoplastic;Humans;Immunotherapy;Lysosomes;Mass Spectrometry;Melanoma;Microscopy, Electron;Mitochondria;Nuclear Proteins;Proteomics;Murinae;calnexin;catenin;cell adhesion molecule;cytochrome c;immunoglobulin;major histocompatibility antigen class 1;mitochondrial protein;protein p120;radixin;tumor antigen;amino acid sequence;article;biogenesis;cancer immunotherapy;cell line;cell lysate;controlled study;electron microscopy;human;human cell;mass spectrometry;melanoma;membrane vesicle;nucleotide sequence;polyacrylamide gel electrophoresis;priority journal;protein analysis;protein isolation;protein purification;sucrose density gradient centrifugation;Western blotting |
| Malignant effusions and immunogenic tumour-derived exosomes | Lancet; 2005 | Andre, F. and Schartz, N.E.C. and Movassagh, M. and Flament, C. and Pautier, P. and Morice, P. and Pomel, C. and Lhomme, C. and Escudier, B. and Le Chevalier, T. and Tursz, T. and Amigorena, S. and Raposo, G. and Angevin, E. and Zitvogel, L. | 10.1016/S0140-6736(02)09552-1 | alpha interferon;carboplatin;carmustine;cyclophosphamide;dacarbazine;docetaxel;doxorubicin;fluorouracil;fotemustine;gamma interferon;gemcitabine;heat shock protein;interleukin 2;major histocompatibility antigen class 1;melphalan;navelbine;oxaliplatin;paclitaxel;sucrose;tetraspanin;thalidomide;tumor antigen;adenocarcinoma;adult;aged;article;ascites;blood cell;carcinomatosis;clinical article;controlled study;cytokine release;cytotoxic T lymphocyte;dendritic cell;female;human;immunoelectron microscopy;lymphocyte activation;male;melanoma;membrane vesicle;mesothelioma;monocyte;pleura effusion;priority journal;ultracentrifugation;Western blotting |
| Induction of lymphocyte apoptosis by tumor cell secretion of FasL-bearing microvesicles | Journal of Experimental Medicine; 2005 | Andreola, G. and Rivoltini, L. and Castelli, C. and Huber, V. and Perego, P. and Deho, P. and Squarcina, P. and Accornero, P. and Lozupone, F. and Lugini, L. and Stringaro, A. and Molinari, A. and Arancia, G. and Gentile, M. and Parmiani, G. and Fais, S. | 10.1084/jem.20011624 | Apoptosis;Melanoma;Melanosome;Microvesicles;T cells;Apoptosis;Blotting, Western;Culture Media, Conditioned;Exocytosis;Fas Ligand Protein;Humans;Immunohistochemistry;Intracellular Membranes;Jurkat Cells;Lymphocytes;Melanoma;Melanosomes;Membrane Glycoproteins;Microscopy, Electron;Secretory Vesicles;Tumor Cells, Cultured;CD63 antigen;FAS ligand;glycoprotein gp 100;antigen expression;apoptosis;article;cellular distribution;controlled study;cytofluorometry;cytoplasm;degranulation;human;human cell;leukemia cell line;lymphoid cell;melanoma;melanoma cell;melanosome;membrane vesicle;priority journal;protein expression;protein localization;T lymphocyte;tumor cell;Western blotting |
| The human melanoma cell line MelJuSo secretes bioactive FasL and APO2L/TRAIL on the surface of microvesicles. Possible contribution to tumor counterattack | Experimental Cell Research; 2005 | MartÃ­nez-Lorenzo, M.J. and Anel, A. and Alava, M.A. and PiÃ±eiro, A. and Naval, J. and Lasierra, P. and Larrad, L. | 10.1016/j.yexcr.2003.12.024 | 3,3â€²- dihexyloxacarbocyanine iodide;APO2 ligand;APO2L;Apoptosis;DiOC<sub>6</sub>(3);Fas ligand;FasL;Human;IEM;Mitochondrial membrane potential;Tumor immunity;Î±-melanocyte stimulating hormone;Î±-MSH;Î”Î¨<sub>m</sub>;alpha intermedin;Fas ligand;phytohemagglutinin;tumor necrosis factor related apoptosis inducing ligand;actin filament;article;blast cell;controlled study;human;human cell;melanoma;melanoma cell;microtubule;priority journal;protein expression;protein secretion;T lymphocyte;tumor immunity |
| Signaling of Tumor-Derived sEV Impacts Melanoma Progression | Int J Mol Sci; 2005 | Zebrowska A, Widlak P, Whiteside T, Pietrowska M. | 10.3390/ijms21145066 | |
